# Prebiotic synthesis of noncanonical nucleobases under plausible alkaline hydrothermal conditions

**DOI:** 10.1038/s41598-022-19474-0

**Published:** 2022-09-07

**Authors:** Cristina Pérez-Fernández, Jorge Vega, Pedro Rayo-Pizarroso, Eva Mateo-Marti, Marta Ruiz-Bermejo

**Affiliations:** Centro de Astrobiología (CAB) CSIC-INTA, Dpto. Evolución Molecular, Carretera de Ajalvir, km 4, Torrejón de Ardoz, 28850 Madrid, Spain

**Keywords:** Analytical chemistry, Chemical biology, Organic chemistry, Origin of life, Polymer chemistry

## Abstract

Herein, the potential of alkaline hydrothermal environments for the synthesis of possible ancestral pre-RNA nucleobases using cyanide as a primary source of carbon and nitrogen is described. Water cyanide polymerizations were assisted by microwave radiation to obtain high temperature and a relatively high pressure (MWR, 180 °C, 15 bar) and were also carried out using a conventional thermal system (CTS, 80 °C, 1 bar) to simulate subaerial and aerial hydrothermal conditions, respectively, on the early Earth. For these syntheses, the initial concentration of cyanide and the diffusion effects were studied. In addition, it is well known that hydrolysis conditions are directly related to the amount and diversity of organic molecules released from cyanide polymers. Thus, as a first step, we studied the effect of several hydrolysis procedures, generally used in prebiotic chemistry, on some of the potential pre-RNA nucleobases of interest, together with some of their isomers and/or deamination products, also presumably formed in these complex reactions. The results show that the alkaline hydrothermal scenarios with a relatively constant pH are good geological scenarios for the generation of noncanonical nucleobases using cyanide as a prebiotic precursor.

## Introduction

**Figure 1:**
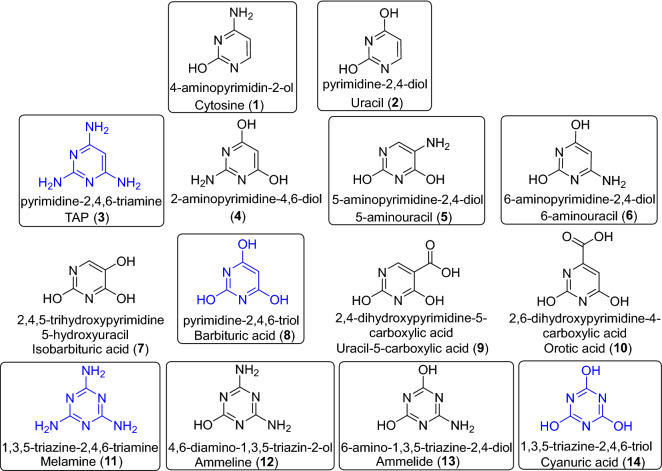
Pyrimidines and triazines taken into account in the present study. All these bases, except for compounds (**9**) and (**10**)**,** are part of a reasonable chemical space considered to contain rational eligible proto-nucleobases, with a total of 91 N-heterocycles^5^. The N-heterocycles in boxes may be forward compatible for pairing with an extant nucleobase in a Watson–Crick-like base pair. In addition, the compounds marked in blue can undergo assemblages with at least one other N-heterocycle of the aforementioned chemical space as monomers in water. Moreover, bases (**3**), (**8**) and (**11**) produce nucleosides with sugars^4,5^. Cytosine (**1**) and uracil (**2**) are extant RNA nucleobases. Compound (**7**) is a possible deamination product of (**5**), and (**9**) can be the deamination product of (**7**). In the same way, (**8**) is a possible deamination product of (**3**) and (**6**), and (**10**) can be the deamination product of (**8**)**.** Compound (**4**) is an isomer of both (**5**) and (**6**), and (**4**) can also be a deamination product of (**3**). In this scheme, only the tautomeric forms identified by GC–MS after derivatization with BSTFA are shown to obtain their corresponding TMS derivatives (for details, please see Supplementary Information, Figures S1–S14). The IUPAC nomenclature and the traditional and/or acronym names are shown.

The generally accepted *RNA world* hypothesis suggests that RNA was used by primordial life of Earth to carry out catalytic and informational functions prior to the current biochemistry based on protein enzymes and DNA^1^. However, the spontaneous generation of RNA under prebiotic conditions has been largely questioned^2^. Thus, some authors have proposed the idea that ancestral polymers were the precursor of RNA^3–5^. A reasonable chemical space of a total of 91 N-heterocycles containing purines, pyrimidines and triazines has been suggested to be a practical candidate for nucleobases of these early proto-RNA polymers, but only 15 purines, 24 pyrimidines and 7 triazines of this chemical space have been considered to be forward compatible with an extant nucleobase in a Watson–Crick-like base pair^5^. In addition, some of these potential prebiological N-heterocycles, such as 2,4,6-triaminopyrimidine (**3**), barbituric acid (**8**), melamine (**11**) and cyanuric acid (**14**), have the capability to form ribonucleosides and supramolecular assemblies that are held by Watson–Crick-type hydrogen-bonded base pairs^6^ (Fig. 1). These candidate ancestral nucleobases spontaneously form glycosidic bonds with ribose and other sugars, and most importantly, functionalized forms of these heterocycles form supramolecular structures and covalent polymers^5^.

Taking into account these proposals for a chemical evolution of RNA, herein, we consider the possibility of the generation of ancestral nucleobases in plausible alkaline hydrothermal scenarios because these environments have been widely recognized as good niches for increasing molecular complexity and, by extension, chemical evolution and, eventually, for the rise of life^7–9^. Specifically, we focus on the pyrimidines and triazines shown in Fig. 1. All of these compounds were previously synthetized under plausible prebiotic conditions but not simultaneously and not under the same synthetic and analytical conditions^10–21^. Compounds (**2**), (**4**), (**5**), (**8**), (**9**), (**10**), (**13**) and (**14**) were identified from the microwave-driven polymerization of cyanide. This result provided promising information about the synthetic possibility of the production of a set of potential ancestral nucleobases under plausible alkaline hydrothermal conditions^20^. However, in this previous work, the GC–MS analytical method was valued for providing an overview of the polar analytes present in HCN polymers but was not specific for the identification of pyrimidines and triazines. For example, (**7**) and (**8**) presented the same retention time and very similar mass spectra. In addition, a substantial number of coelution peaks were found, and several molecules could not be properly identified because they could be assigned to different N-heterocyclic isomers. Additionally, some organics identified may be the decomposition and deamination products of other ones that may have been produced during the hydrolysis processes used in the work-up of the samples. All of these factors encouraged us to develop a specific methodology for the identification of candidates of ancestral nucleobases from complex mixtures formed under simulated alkaline hydrothermal conditions, considering the potential of these environments in the hypotheses regarding the origin of life, as mentioned above, and the more plausible conditions that might have led to an increase in molecular complexity, taking cyanide as a key prebiotic molecule.

**Figure 2:**
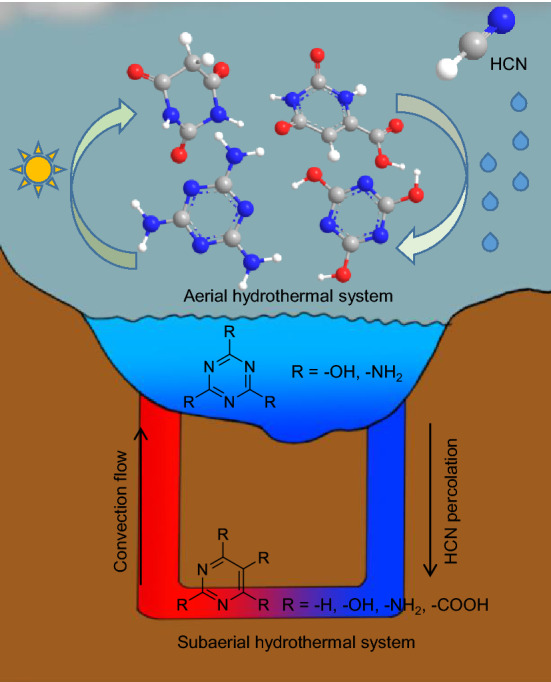
Plausible geological scenario for the synthesis of pyrimidines and triazines, showing aerial and subaerial alkaline hydrothermal conditions and the possible flow of HCN and organic compounds around the cycle. In this scenario, the atmospheric HCN, which can be produced through several pathways^27–30,32,37,38^, would rain fall over an alkaline aerial hydrothermal system. In this way, the dissolved atmospheric HCN would be concentrated tough wet-dry cycles and then driving the subsequent aqueous reactions to the preferential generation of triazines. Moreover, the concentrated cyanide might be percolated to the subaerial part of the hydrothermal system, leading to the preferential synthesis of pyrimidines. Again, thanks to the convection flow the pyrimidines would be in touch with triazines in the upper part of the hydrothermal system and the wet-dry cycles might favour the production of supramolecular assemblies such as melamine (**11**):cyanuric acid (**14**) and barbituric acid (**8**): melamine (**11**). Experimentally to simulate this plausible alkaline scenario, NH_4_Cl has been used to adjust the pH. This soluble salt could be formed and concentrated in analogous way to the HCN. Atmospheric NH_3_ is dissolved in the aqueous environment and concentrated by evaporation as NH_4_Cl, due to the general presence of chloride salts in the geochemical aqueous pools^39^.

HCN chemistry has attracted considerable attention in research on the origin of life, for example, in the experimental proposals made by Sutherland regarding the common origin of RNA, protein and lipid precursors in cyano-sulfidic proto-metabolism^22^. This work, together with the “glyoxilate scenario” suggested by Eschenmoser^23,24^, conveys that the reaction networks that describe plausible protometabolic systems are based on HCN homologation. In fact, aqueous HCN chemistry has recently been considered because computational analyses suggest that simply HCN and water may be the precursors of RNA and proteins^25^. Moreover, aqueous cyanide polymerizations have traditionally been considered preferential routes for the prebiotic synthesis of purines and pyrimidines^14,21,26^. HCN has been proposed to have existed in the ancient Earth's atmosphere and in hydrothermal environments, both submarine and subaerial^8,27–31^. Moreover, recently, taking into account possible conditions of the early Earth´s atmosphere, it has been showed by a numerical model that the calculated HCN rain-out to surface warm little ponds could lead the subsequent aqueous reactions of HCN into important biomolecules such as purines and pyrimidines up to micromolar concentrations^32^. On the other hand, it is well known that HCN only efficiently oligomerizes/polymerizes in alkaline aqueous environments (pH range from 8 to 10)^33^ using concentrations generally greater than 0.01 M, because in more dilute solutions, hydrolysis is the dominant process^34^. Since the HCN is more volatile than water (bp ≈ 25 °C), HCN cannot be concentrated by evaporation if the pH is lower than its pK_a_ (9.2). However, the likely presence of alkaline warm little ponds or alkaline aerial hydrothermal systems and seasonal wet-dry cycles could favour the concentration of the atmospheric rain-out HCN as cyanide in these plausible water pools of the ancient Earth^32,35^. Moreover, the discovery of alkaline lakes on current Earth, with pH values between 9 and 12, present a truly possibility for the concentration of cyanide by evaporation in the primitive Earth^36^. Moreover, concentrated aqueous cyanide flows may be percolated through the mineral to the subaerial part of the hydrothermal system, as it is showed in the Fig. 2.

Thus, in this work, alkaline hydrothermal conditions were simulated using several initial concentrations of cyanide (0.05, 0.25 and 1 M) to explore the suitability of these environments for the production of pre-RNA nucleobases. Specifically, to simulate the aerial part of the alkaline hydrothermal system, polymerizations at 80 °C, at pH 9.2 and at atmospheric pressure using a conventional thermal system (CTS) were carried out. Moreover, to mimic the subaerial environment, cyanide microwave-driven polymerization at 180 °C, pH 9.2 and a maximum work pressure of 15 bar were driven. The pH was adjusted using equimolar concentrations of NH_4_Cl. This soluble salt has been revealed as an important reactant to the plausible prebiotic production of RNA precursors^39^. The mechanism of concentration of NH_4_Cl in the geological local scenario here proposed could be analogous to that proposed for the HCN (Fig. 2). Additionally, the diffusion effects were taken into consideration, since cyanide polymerizations are generally carried out without stirring and a full mixture of the final products must be considered in hydrothermal systems. To the best of our knowledge, this is the first time that the effects of diffusion have been taken into account in this type of one-pot prebiotic synthesis. The third factor to be considered was the dynamism of the current terrestrial hydrothermal systems, which represent a wide range of pH gradients (pH = 2–11), along the hydrothermal fields. The potential cyanide polymers formed in these environments on the early Earth were probably continuously exposed to thermolysis and hydrolysis reactions. Because hydrolysis conditions (i.e., heating and pH value) are directly related to the amount and diversity of organic molecules released from cyanide polymer synthesis, as a first step, we studied in detail the effect of the hydrolysis procedures using neutral, alkaline and acid conditions for each of our 14 compounds of interest and later over the synthetic complex mixtures from the cyanide polymerizations. In this way, it was possible to test the stability of the base pre-RNA candidates against pH gradients and to detect them in alkaline hydrothermal environments from cyanide polymerization. As expected, the diversity, types and yields of the N-heterocycles identified were directly dependent on the simulated synthetic environments and on the hydrolysis conditions used. Thus, herein, the potential of different alkaline hydrothermal environments for the generation of building blocks present in plausible protoinformational polymers using cyanide as a primary source of carbon and nitrogen is described.

## Materials and methods

### Preparation of standard solutions and authentic standards. Quantification by multiple-point external standardization

For each of the compounds considered here (Fig. 1), aqueous solutions of known concentrations were prepared using Milli-Q water at an initial concentration of 1000 ppm. In some cases, it was necessary to adjust the pH to achieve complete dissolution of the standards. All these stock solutions were in turn diluted 1:10. From these samples, 100 µL was frozen and freeze-dried and then derivatized with BSTFA with 1% TMCS (BSTFA = *N*,*O*-bis(trimethylsilyl)trifluoroacetamide, TMCS = trimethylchlorosilane, obtained from Thermo Scientific) to obtain their corresponding TMS derivatives, as explained below, and used for analysis by GC–MS, as indicated in the following section.

All the pyrimidines and triazines shown in Fig. 1 are available commercially and were supplied by Sigma–Aldrich, Merck, Fluka, or Panreac (for further details, see Supplementary Information).

In addition, barbituric acid (**8**), orotic acid (**10**), melamine (**11**) and cyanuric acid (**14**) were quantified by multiple-point external standardization method. Calibrate lines for each analyte were calculated using at least six standard solutions with concentration from 10 to 150 ppm. The standard solutions were injected × 3 in the GC–MS equipment to sure the reproducibility of the measurements.

### Hydrolysis conditions

To check the effect of the hydrolysis conditions on the authentic standards shown in Fig. 1, 100 µL of the 1:10 diluted stock solutions of each analyte was frozen and freeze-dried and then treated with 0.5 mL of the corresponding acidic, basic or neutral solution under the detailed conditions indicated below. Finally, the hydrolysed final products were again freeze-dried and then derivatized with BSTFA and used for GC–MS analysis.

The hydrolysis conditions were chosen based on previous works on prebiotic chemistry^20,40–42^: a) acid conditions: heating at 110 °C in 6 N HCl for 24 h; b) basic conditions: heating at 110 °C in 5 N NH_4_OH for 24 h; c) neutral conditions (slightly alkaline conditions): heating at 140 °C in a phosphate buffer solution (0.01 M, pH 8) for three days and taking into account the plausible pH gradient in hydrothermal systems or pH environmental variations.

These hydrolysis conditions were also used to treat the cyanide polymers and/or the fractions from the syntheses under plausible alkaline hydrothermal conditions. For each case, 2–30 mg of the sample (for details, please see the Supplementary Information) was added to 0.5 mL of the corresponding hydrolyser solution. Then, all the hydrolysed samples were freeze-dried.

### GC–MS analysis

Prior to injection on the GC–MS equipment, 100 µL of BSTFA with 1% TMCS was added to the freeze-dried standard solutions prepared as explained above, to their corresponding hydrolysed products and to the unhydrolysed/hydrolysed synthetic alkaline hydrothermal samples. The final solutions/suspensions obtained were heated at 80 °C for 3 h to obtain the respective trimethylsilyl derivatives.

The GC–MS analysis was performed in full-scan mode using a mass range of 60–550 uma on a 6850 GC chromatograph coupled to a 5975 VL MSD triple-axis detector in electron impact mode (EI) at 70 eV (Agilent) using an HP-5 MS column (30 m × 0.25 mm × 0.25 µm thick) and helium (He) as the carrier gas. The following temperature ramp was used: 80 °C (initial temperature), with a hold of 1.5 min, heated to 230 °C at 5 °C min^-1^, with a hold time of 5 min and heated to 300 °C at 25 °C min^-1^ with a final hold of 10 min. Two microlitres of each sample was injected. The temperature of the injector was 300 °C, and the injections were performed in splitless mode. The detector temperature was 300 °C. The flow rate was 0.9 mL·min^-1^. The temperature ramp and the flow rate were systematically modified to obtain an adequate separation and identification of the chromatographic peaks.

### Cyanide polymerizations as a model of alkaline hydrothermal environmental prebiotic chemistry

To simulate subaerial alkaline environments, NH_4_CN polymerization reactions were carried out in aqueous solution using equimolar amounts of NaCN and NH_4_Cl at different concentrations (1 M, 0.25 M, 0.05 M, final volume 11 mL and initial pH 9.2) with a Biotage Initiator^+^ microwave reactor (Biotage, Sweden) and 20 mL capacity vials using the same heating ramp described in a previous study^20^. The final temperature was 180 °C, and the reaction time in all cases was 20 min. The maximum pressure reached was 15 bar. The microwave reactor system allows to choose the working temperature and the pressure is automatically adjusted, with a maximum working pressure of 20 bar. All reactions were carried out under anoxic conditions using an inert atmosphere of nitrogen. The vials were purged several times with nitrogen, and the water solutions were also bubbled with nitrogen to ensure the lack of air in all the systems. After the reaction time, on the one hand, the final suspensions were frozen and freeze-dried to constant weight, and on the other hand, for a more exhaustive analysis of the samples, the final suspensions were filtered and treated as described in ^20^, and gel and sol fractions were collected. The sol fractions were subsequently concentrated by ultrafiltration using centrifugal devices with a cut-off of 3 kDa. Additionally, note that all the samples analysed by GC–MS were previously freeze-dried.

To recreate aerial alkaline environments, in conditions analogous to the microwave-driven polymerization of cyanide, equimolar solutions of NaCN and NH_4_Cl of concentration 1 M were heated at 80 °C for 15 days under anoxic conditions. This reaction time was chosen based on the table of equivalences provided by the manufacturer of the microwave reactor. Twenty minutes at 180 °C using the microwave reactor is equivalent to 15 days at 80 °C using a CTS. The crude reactions were filtered and treated as described in^20^, and the gel and sol fractions were collected.

In the static experiments, the initial solutions of cyanide remained during the indicated reaction times. For the stirring experiments, the cyanide solutions were magnetically stirred at 300 rpm.

## Results

### Development of the analytical GC–MS method for pyrimidines and triazines

The previous analysis of some cyanide polymers synthetized using alkaline hydrothermal conditions suggested that these polymers can be precursors of the noncanonical nucleic bases mentioned above^20^. Thus, as a first step, herein, we generated a specific analytical method based on GC–MS as the main technique to create a library of chromatograms and mass spectra using standard solutions of the analytes shown in Fig. 1. To develop the analytical method, we took into consideration the previously reported methodology^17,43^. Using these initial analytical conditions, it was observed that the separation of the pyrimidines and triazines of interest was not optimal, so the parameters of the GC–MS were modified until the conditions described in Sect. “GC–MS analysis” were reached. For the analytical method, we used 100 ppm standard solutions of all the compounds presented in Fig. 1. Each solution was analysed individually, and in most cases, only a single chromatographic peak was observed, which appears to correspond to the derivative tri-substituted with trimethylsilyl groups (3 TMS). However, two chromatographic peaks were observed corresponding to the di-substituted derivatives (2 TMS) and the tri-substituted derivatives (3 TMS) for compounds (**4**) and (**11**), and two peaks corresponding to the tri-substituted derivative (3 TMS) and the tetra-substituted derivative (4 TMS) were detected for compound (**13**). In addition, in the particular case of cytosine (**1**) and uracil (**2**), only one chromatographic peak corresponding to the di-substituted derivative (2 TMS) was observed. All the chromatograms and their respective mass spectra for the authentic standards shown in Fig. 1 are reported in Figures S1-S14. The superposition of the fourteen chromatograms led to an acceptable separation of the chromatographic peaks that could be used satisfactorily for the identification of the noncanonical nucleobases of interest in our hydrothermal synthetic samples.

### Effects of the hydrolysis conditions

Variations in pH are expected in both natural subaerial hydrothermal systems and aerial systems due to environmental conditions. These pH variations could lead to the delivery of important bioorganics to cyanide polymers. Generally, these pH variations are interpreted experimentally in the laboratory as “hydrolysis conditions”, which, together with relatively high temperatures, speed up the natural hydrolysis processes^44^. It is well known that the use of these hydrolysis conditions increases the number and diversity of the analytes identified from HCN polymers and therefore significantly affects the overall results of the analyses^26,41,42^. However, the influence of the hydrolysis conditions is often considered with respect to the final products of the oligomerization/polymerization reactions. To our knowledge, only one work has studied the effect of hydrolysis conditions on compounds of interest obtained from cyanide polymerizations, in that case, pteridines^40^. Thus, the effect of three hydrolysis conditions, usually considered in prebiotic chemistry experiments^20,40–42^, on each of the molecules shown in Fig. 1 was studied since, as indicated above, these conditions can have a notable effect on the analytical results.

**Figure 3:**
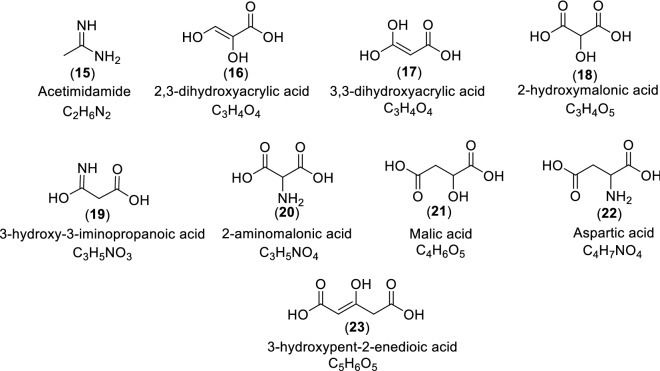
Decomposition products identified after hydrolysis of the pyrimidines and triazines shown in Fig. 1 and indicated in Table 1. All of these molecules were assigned based on their respective TMS derivatives after derivatization with BSTFA. When available, the identified compounds were confirmed against authentic standard mass spectra and retention times. In contrast, when not available, the polar organic compounds were identified by searching their mass spectra in the NIST database. For identification purposes, we considered only peaks with a signal-to-noise ratio over 5. Those peaks for which the match probability in the database was below 90% and/or tentatively or ambiguously identified were considered unidentified and are not discussed herein.

Semiquantitative results are shown in Table 1 regarding the hydrolysis processes influencing the pyrimidines and triazines of Fig. 1. In addition, all the chromatograms corresponding to these hydrolysis reactions are shown in the Supplementary Information (Figures S15–S28), and Fig. 3 shows some of the heterocyclic decomposition products of the target N-heterocycles shown in Fig. 1. In general, it can be observed for some molecules that the hydrolysis conditions have no effect; in other cases, total or partial deamination occurs, and in some cases, transposition processes seem to take place, and oxidative cleavages were also observed. In particular, cytosine (**1**), uracil (**2**), orotic acid (**10**) and cyanuric acid (**14**) were not affected under any of the hydrolysis conditions used, and no change was observed. In the case of TAP (**3**), it was observed that the acidic conditions led to a partial deamination, and the basic and neutral hydrolysis conditions led to a total decomposition of the molecule. For 2-aminopyrimidine-4,6-diol (**4**) subjected to acidic hydrolysis conditions, no change was observed. For basic hydrolysis conditions, we observed several transformations. On the one hand, barbituric acid (**8**) was identified, and on the other hand, there was a small contribution from degradation products such as 2,3-dihydroxyacrylic acid (**16**), 3-hydroxy-3-iminopropanoic acid (**19**), and 3-hydroxypent-2-enedioic acid (**23**) (Fig. 3). In the case of neutral hydrolysis, acetimidamide (**15**) was produced as a degradation product in a ≈ 1:2 ratio. 5-aminouracil (**5**) yielded isobarbituric acid (**7**) as a deamination product under acid hydrolysis conditions, while under basic conditions, the decomposition products were mainly identified, such as 2-aminomalonic acid (**20**) together with unreacted (**5**) and orotic acid (**10**). Neutral hydrolysis conditions did not lead to changes. The acid hydrolysis of 6-aminouracil (**6**) led mainly to barbituric acid (**8**), and in lower amounts to (**19**) and (**23**), while under basic and neutral hydrolysis, compound (**6**) did not undergo any change. Isobarbituric acid (**7**) did not present any change under acidic hydrolysis conditions. However, under basic conditions, we identified malic acid (**21**) and aspartic acid (**22**) as decomposition products, and orotic acid (**10**). In the case of neutral hydrolysis, orotic acid (**10**) and malic acid (**21**) were obtained. Barbituric acid (**8**) underwent partial decomposition under acidic conditions, obtaining compounds (**17**), (**19**) and (**23**). Under basic and neutral hydrolysis conditions, no transformation of the analyte was observed. For molecule (**9**), only the acidic hydrolysis conditions had a slight influence, leading to a minor partial decomposition. Acidic hydrolysis led to the total deamination of melamine (**11**), producing cyanuric acid (**14**); under basic conditions, no transformations were observed, while neutral hydrolysis conditions led to the partial deamination product (**13**) together with the total deamination product (**14**). Ammeline (**12**), under acidic conditions, underwent a total deamination to cyanuric acid (**14**); under basic conditions, it was partially deaminated, obtaining ammelide (**13**); and under neutral hydrolysis, partial (**13**) and total (**14**) deamination products were obtained. Ammelide (**13**) had transformations similar to those of melamine (**11**). Under acid hydrolysis, it was transformed to cyanuric acid (**14**); under basic conditions, no change was observed; and under neutral conditions, a transformation to cyanuric acid (**14**) in a ≈ 2:1 ratio was observed.

**Table 1:**
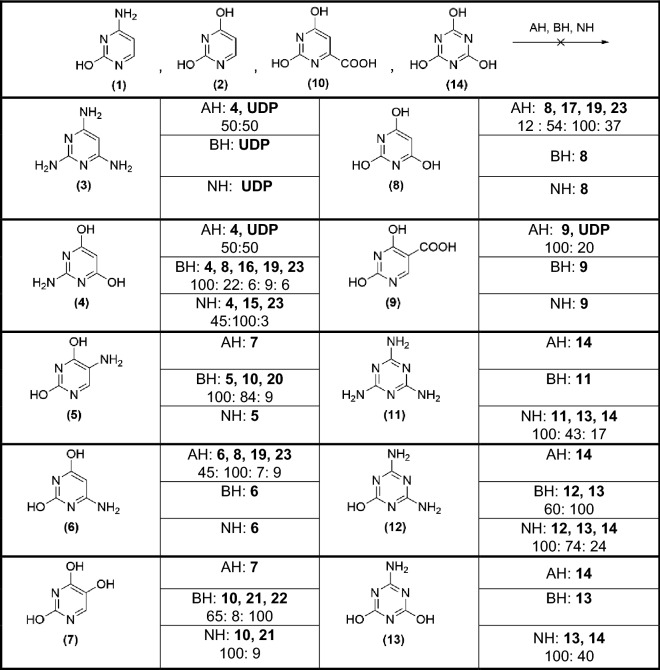
Semiquantitative analysis of the effect of the hydrolysis conditions in the set of pyrimidines and triazines considered in the present study (Fig. 1).

AH = acid hydrolysis conditions (HCl 6 N/110 °C/24 h); BH = basic hydrolysis conditions (NH_4_OH 5 N/110 °C/24 h); NH = neutral hydrolysis conditions (phosphate buffer pH = 8/140 °C/72 h). A value of 100 indicates the peak with the greatest area in each chromatogram, and the ratios of the other peaks are based on this value (Figures S15-S28). UDP = unknown decomposition product.

As important considerations of these hydrolysis assays and the recent interpretation of the GC–MS analytical results from the cyanide polymeric complex mixtures, one can say that (i) compound (**3**) could only be identified in nonhydrolysed samples; (ii) (**7**) could only be observed after acidic hydrolysation of the complex mixtures of the reaction or in nonhydrolysed samples; and (iii) analytes (**5**), (**11**), (**12**) and (**13**) could not be detected after acid hydrolysis of the samples. Therefore, in general, the group of pyrimidines examined in the present study would be influenced to a greater extent by the effect of the neutral and basic hydrolysis conditions, while the group of triazines would be influenced by the acidic hydrolysis conditions. These facts might have a direct influence on prebiotic generation in natural environments.

### Concentration and diffusion effects in the production of pre-RNA nucleobases

The polymerization of cyanide assisted by MWR was chosen as the first step to explore the potential of alkaline hydrothermal systems as niches for the generation of noncanonical nucleobases. Different concentrations of cyanide were selected since this factor is key in cyanide polymerization and because the likely mechanisms for the production of concentrated cyanide solutions (< 0.01 M) in natural prebiotic scenarios is not yet a conclusively resolved issue in prebiotic chemistry^22,36,45–48^, although as it was indicated in the Fig. 2, herein it has been proposed a concentration mechanism by evaporation in an alkaline scenario. Thus, the directly freeze-dried crude reactions, from hydrothermal syntheses at 180 °C using initial concentrations of 0.05, 0.25 and 1 M with and without stirring, were GC–MS analysed for pyrimidines and triazines before and after hydrolysis treatment, without any preconcentration or separation procedure before the derivatization of the samples with BSTFA to obtain the corresponding TMS derivatives.

**Figure 4:**
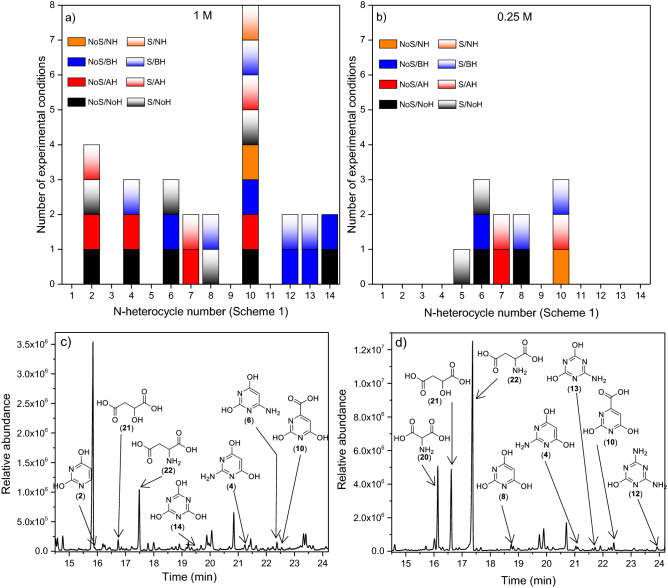
Summary of the qualitative GC–MS analytical results for pyrimidines and triazines from cyanide polymerization under subaerial alkaline hydrothermal conditions (MWR, 180 °C) using different initial concentrations: (**a**) 1 M and (**b**) 0.25 M. The numbers on the abscissa axis correspond to the enumeration of the N-heterocycles shown in Fig. 1. NoS = static experiments without stirring; S = stirring experiments; NoH = nonhydrolysed samples; AH = acid hydrolysed samples; BH = basic hydrolysed samples; NH = neutral hydrolysed samples. Representative chromatograms of the 1 M series: (**c**) nonhydrolysed sample from a static experiment and (**d**) basic hydrolysed sample from a stirring experiment.

The results of all these qualitative analyses are summarized in Fig. 4a–b, and representative chromatograms of this analytical study are shown in Fig. 4c–d (for the detailed results and chromatograms, please see Figures S29-S33). Note that only pre-RNA nucleobases were identified in the experiments using the higher concentrations of cyanide used here, 0.25 M and 1 M. We were not able to identify any compound using an initial concentration of 0.05 M (Figure S33). Therefore, it seems that high concentrations of cyanide are needed for the production of ancestral pre-RNA nucleobases. In fact, the diversity of N-heterocycles is greater in the syntheses carried out using the highest concentration of cyanide considered herein, 1 M, than in the experiments using an initial concentration of 0.25 (please compare Fig. 4a,b). On the other hand, diffusion effects through the stirring of the reaction mixtures in this type of hydrothermal synthesis seem to have no significant influence on the diversity of the N-heterocycles identified.

As shown in Fig. 4a, cytosine (**1**), TAP (**3**), 5-aminouracil (**5**) and melamine (**11**) were not detected under any of the conditions considered. Taking into account the results shown in Table 1, cytosine (**1**) could be identified using any of the hydrolysis conditions considered; TAP (**3**) could only be identified in unhydrolysed samples, and 5-aminouracil (**5**) and melamine (**11**) could be detected in unhydrolysed samples or using basic or neutral hydrolysed conditions. Considering that, generally, the hydrolysis processes increase the amount and diversity of the analytes identified in HCN oligomers/polymers, it seems that cytosine (**1**) and melamine (**11**) cannot be produced under plausible subaerial hydrothermal conditions using cyanide as a prebiotic source of carbon and nitrogen. However, the production of (**3**) and (**5**) is uncertain because the detection of (**4**) may be from the partial deamination of (**3**) under acidic hydrolysis conditions, and the same is possible for (**5**) leading to the identification of (**7**). In addition, orotic acid (**10**) was largely identified. Note that its detection under basic and neutral conditions could influence the additional contribution of the deamination of (**5**) and (**7**) compounds. Finally, basic conditions appear to assist the production of triazines (Fig. 4a).

### Generation of pre-RNA nucleobases under simulated subaerial and aerial hydrothermal conditions

Taking into account these preliminary results regarding the plausible prebiotic synthesis of noncanonical nucleobases, additional cyanide polymerizations were carried out at an initial concentration of 1 M. Thus, new syntheses were carried out using CTS at 80 °C to simulate aerial hydrothermal conditions. On the other hand, all crude reactions obtained under subaerial conditions as well as under aerial conditions were filtered to collect gel and sol fractions (black insoluble polymers and soluble fractions, respectively). Furthermore, the sol fractions were concentrated using ultracentrifugal devices with a cut off of 3 kDa to obtain light fractions (F < 3 kDa) and heavy fractions (F > 3 kDa). Table 2 shows all the reaction conditions considered and the yields of all fractions and subfractions collected (three independent reactions were carried out under each of the four experimental conditions considered, in order to check the reproducibility of the cyanide polymerization processes). All these fractions were analysed by GC–MS for pyrimidines and triazines in a manner analogous to that indicated above. However, only acid and basic hydrolysis conditions were considered since the neutral hydrolysis conditions do not seem to increase the diversity of the N-heterocycles identified (Fig. 4). Note that, generally, the separation of the crude reactions in several fractions leads to a better and easier identification of the targets^19^.

**Table 2:** Reactions were carried out to simulate alkaline hydrothermal systems under subaerial conditions (MWR, 180 °C, entries 1 and 2) and under aerial environments (CTS, 80 °C, entries 3 and 4).

Entry	T (°C)	c (M)^[a]^	Stirring	Reaction Time	α (%)^[b]^	% lost weight ^[c]^	F > 3 kDa (%)^[d]^	Acid hydrolysed gel fraction (%)^[e]^
1	180	1	−	20 min	15 ± 2	23 ± 5	30 ± 1	48
2	180	1	+	20 min	9 ± 1	27 ± 2	21 ± 1	46
3	80	1	−	15 days	35 ± 5	25 ± 3	27 ± 1	37
4	80	1	+	15 days	18 ± 4	21 ± 2	21 ± 1	37

All reactions were carried out under an inert nitrogen atmosphere.^[a]^ Initial concentrations of NaCN and NH_4_Cl (equimolar) in aqueous solutions.^[b]^ α (%) = conversion degree for the gel fractions [α (%) = [(mg of gel fractions)/(initial mg of CN^-^)] * 100]. Note the greater amount for the insoluble solids obtained from the no-stirring experiments. The values of α (%) obtained herein are in strong agreement with previous results^20,49^.^[c]^ % lost weight = [(initial mg of NaCN + mg NH_4_Cl)-(mg of freeze-dried sol fractions + mg gel fraction)/(initial mg of NaCN + mg NH_4_Cl)] *100. The lost weights (likely due the loss of NH_3_ and other volatiles during the polymerization processes) are similar in all cases.^[d]^ F > 3 kDa (%) = [(mg of F > 3 kDa)/(mg of freeze-dried sol fractions)] *100. The F > 3 kDa (%) is lower for the stirring experiments, as in the cases for the α (%), which seems to indicate that the stirring of the systems decreases the production of heavier macromolecular fractions.^[e]^ % of the gel fraction susceptible to acid hydrolysis. Acid hydrolysed gel fraction (%) = (mg of soluble supernatant after acid hydrolysis of the gel fraction/mg of gel fraction)*100. These percentages are in good agreement with those previously reported^20^. At least three independent experiments were carried out for each experimental condition to calculate the average values shown in this table and to check the reproducibility of the cyanide polymerization processes.

**Figure 5:**
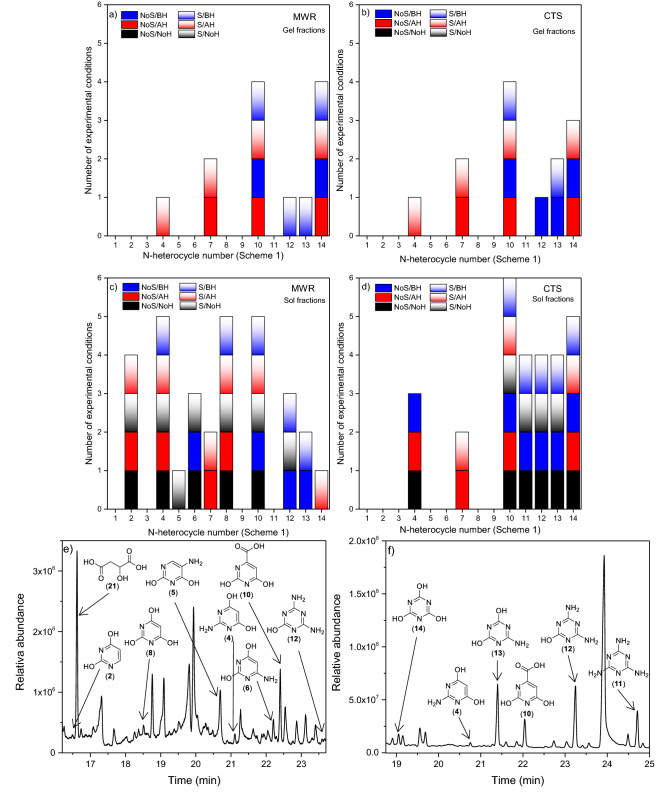
Summary of the qualitative GC–MS analysis for pyrimidines and triazines from alkaline hydrothermal cyanide polymerization under plausible subaerial conditions (MWR, 180 °C) and aerial conditions (CTS, 80 °C) of (**a**) and (**b**) gel fractions (insoluble polymers) and (**c**) and (**d**) sol fractions (soluble fractions). The numbers on the abscissa axis correspond to the enumeration of the N-heterocycles shown in Fig. 1. NoS = static experiments without stirring; S = stirring experiments. NoH = nonhydrolysed samples; AH = acid hydrolysed samples; BH = basic hydrolysed samples. Representative chromatograms of these types of analysis: (**e**) nonhydrolysed sol fraction from the assisted MW polymerization of cyanide in a stirring experiment; and (**f**) nonhydrolysed sol fraction from the cyanide polymerization under plausible aerial conditions in a static experiment. The scale of retention time in both chromatograms is different for better visualization of the analytes.

The qualitative GC–MS analytical results for the gel and sol fractions from the MWR and CTS experiments are represented in Fig. 5a–d, respectively. Representative chromatograms of this analytical series are shown in Fig. 5e–f (for details, please see Figures S34–S39. Additionally, the good reproducibility of the analytical results from GC–MS is exemplary showed in the Figures S40-S41). It is notable that N-heterocycles are only identified in all gel fractions after hydrolysis processes (Fig. 5a–b). Moreover, the identification of triazines is directly related to basic hydrolytic processes, both in MWR and CTS polymerizations. However, the number of pyrimidines and triazines identified in the gel fractions from MWR experiments is greater under stirring synthesis conditions, whereas this diversity in triazines is higher in the static experiments from the CTS polymerizations. On the other hand, it is remarkable that sol fractions are much richer in N-heterocycles than gel fractions since pyrimidines as well as triazines are detected before any hydrolysis treatment (Fig. 5c–5d). Interestingly, considering the qualitative analysis of the sol fractions, the simulated subaerial hydrothermal conditions seem to improve the production of pyrimidines, while the simulated aerial hydrothermal environments favour the generation of triazines, including melamine (**11**). As in the case of the gel fractions, greater diversity in N-heterocycles is observed in the stirring MWR polymerizations and in the static CTS experiments.

Based on these results, it seems that cytosine (**1**) and TAP (**3**) cannot be synthetized from cyanide under hydrothermal synthetic conditions, at least using the conditions simulated in the present study. However, uracil (**2**), 5-aminouracil (**5**), 6-aminouracil (**6**), barbituric acid (**8**), melamine (**11**), ammeline (**12**), ammelide (**13**) and cyanuric acid (**14**), which may be forward compatible for pairing with an extant nucleobase in a Watson–Crick-like base pair, could be identified under several conditions. To deepen this result, a semiquantitative analysis of these molecules was carried out together with orotic acid (**10**) due to the potential role of (**10**) in chemical pathways to RNA^50–52^*.*

**Figure 6:**
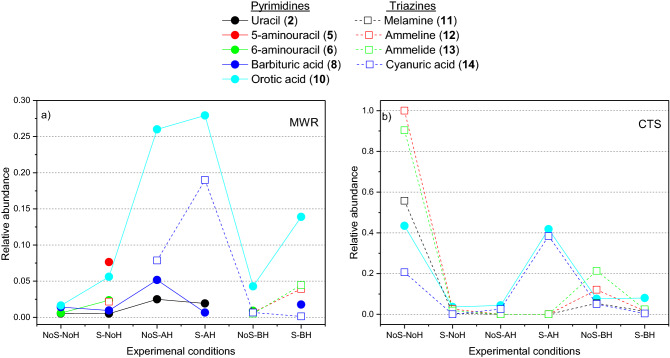
Results of the semiquantitative GC–MS analysis for pyrimidines and triazines from the cyanide polymerizations under plausible alkaline hydrothermal conditions, simulating (**a**) subaerial environments (MWR, 180 °C) and (**b**) aerial environments (CTS, 80 °C). For each analyte, the contributions from the gel and sol fraction chromatographic peaks are considered. The abscissa axis indicates the experimental conditions used in each case: NoS = static experiments without stirring; S = stirring experiments. NoH = nonhydrolysed samples; AH = acid hydrolysed samples; BH = basic hydrolysed samples. The value 1 corresponds to the addition of the area of the chromatographic peak from the gel fraction plus the area of the chromatographic peak from the sol fraction of the ammeline (**12**) from the conventional heating polymerization of cyanide in a no stirring experiment (CTS, NoS-NoH). All areas were normalized, taking into account the amount of mg used for each TMS-derivatized sample.

**Figure 7:**
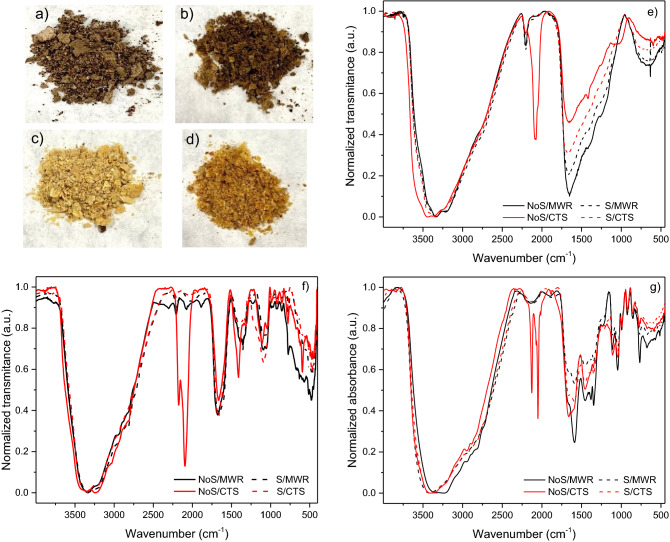
Images of the sol fractions: (**a**) from a static experiment under simulated subaerial conditions (MWR, 180 °C, NoS); (**b**) from a stirring experiment under simulated subaerial conditions (MWR, 180 °C, S); (**c**) from a static experiment under simulated aerial conditions (CTS, 80 °C, NoS); and (**d**) from a stirring experiment under simulated aerial conditions (CTS, 80 °C, S). Representative FT-IR spectra from (**e**) gel fractions; (**f**) heavy fractions (F > 3 kDa) and (**g**) light sol fractions (F < 3 kDa).

Thus, the semiquantitative analyses of compounds (**2**), (**5**), (**6**), (**8**), (**10**), (**11**), (**12**), (**13**) and (**14**) for the simulated subaerial systems and aerial environments are shown in Figs. 6a and b, respectively. Higher yields were obtained for triazines and orotic acid under aerial conditions in a static experiment (Fig. 6b). Notably, the yields for the analogous conditions with stirring were clearly lower. On the other hand, there is an unexpected decrease, or no effect, in the relative amounts of triazines identified after basic hydrolysis conditions (Fig. 6b) since these conditions have no effect on these compounds, as explained in the first part of this work. Nevertheless, these same basic hydrolysis conditions led to the identification of triazines in the MWR experiments (Fig. 6a). These apparent contradictory results may be due to the different natures of the gel and sol fractions obtained in both sets of experiments, MWR and CTS, as shown in Fig. 7. Generally, it is accepted that the MW radiation only reduces the reaction time in organic synthesis. However, in the present case, as it is a highly complex reaction as cyanide polymerization, MW radiation has a clear effect on the generation of interesting prebiotic N-heterocycles against CTS. If the effect of pressure and temperature produced by the MWR could be considered a good simulation of a hydrothermal subaerial environment, the cyanide chemistry in this type of scenario would be truly different from that in hydrothermal aerial environments. This fact is in strong agreement with previous results that tested the high sensitivity of cyanide polymerization to the experimental conditions^21^. Additionally, we carried out a quantification of the compounds (**8**), (**10**), (**11**) and (**14**), as main molecules found in this study, by the multiple-point external standardization method. For the experiments with the higher yields obtained, taking in consideration the semiquantitative results, the quantification was as follow: 0.07, 0.36 and 0.11 ppm/mg for (**8**), (**10**) and (**14**) were found, respectively, in the MWR-driven polymerization without stirring after acid hydrolysis (NoS-AH, Fig. 6a); for the conventional thermal heating polymerization without stirring (NoS-NoH, Figura 6b), 0.60, 0.77 and 0.29 ppm/mg were calculated for (**10**), (**11**) and (**14**), respectively. Moreover, quantitatively the effect of the stirring can be exemplified by the amount of melamine in the basic hydrolysis samples leading to 0.09 and 0.02 ppm/mg, in the no stirring and stirring experiments, respectively (NoS-BH and S-BH, Fig. 6b).

## Discussion

The results reported herein suggest that the subaerial environments would favour the generation of pyrimidines at high concentrations of cyanide, but in contrast, the aerial hydrothermal alkaline scenarios would notably improve the production of triazines. Moreover, it seems that an alkaline hydrothermal pool is a preferential scenario, initially, for the concentration of cyanide by evaporation and subsequently for the production of triazines. Additionally, it was suggested that these environments should favour the production of noncanonical ribonucleosides^35,53^. In addition, under such conditions, the simultaneous generation of cyanuric acid (**14**) and adenine from cyanide was shown^20^. This result is interesting since it has been revealed that compound (**14**) by itself can facilitate the self-assembly of adenine-based systems^54,55^, suggesting new possibilities at monomeric and oligomeric levels in a pre-RNA world^6^.

On the other hand, the formation of supramolecular aggregates of barbituric acid (**8**) and melamine (**11**) containing Watson–Crick-like base pairing has been shown^35^. Moreover, TAP (**3**), barbituric acid (**8**) and melamine (**11**) present the capacity to produce supramolecular assemblies, comprising (**8**):(**11**)^4^, (**3**):(**8**)^56^, (**11**):(**14**)^57,58^, and (**3**):(**14**) assemblies^59,60^. Taking into account the results shown in Figs. 5a-d and 6a-b, it is remarkable that TAP (**3**) cannot be formed under any of the conditions considered herein and that barbituric acid (**8**) is only formed under subaerial conditions. Thus, the only direct formation of a supramolecular assembly would be (**11**):(**14**) under wet–dry cycles in a plausible aerial hydrothermal system using cyanide as a prebiotic starting reactant. However, the plausible dynamics of the hydrothermal systems must be taken into consideration. In this way, it may be possible to drive the N-heterocycles from the aerial part of the system to the subaerial scenario and back to the aerial conditions, as proposed in Fig. 2. The relative constancy of an alkaline pH would be the only condition for the generation of supramolecular assemblies such as (**11**):(**14**) and (**8**):(**11**) under the alkaline hydrothermal scenario proposed here due to the possible degradation of (**8**) and (**11**) under acidic conditions.

Moreover, the formation of orotic acid (**10**) with relatively high yields under any of the experimental conditions assayed herein is interesting. Thus, it seems that the synthesis of this compound from cyanide is indeed favoured and robust under several possible prebiotic scenarios. Orotic acid (**10**) has the capability to form orotidine by reaction with a ribose–phosphate derivative^50^, and orotidine-5′-phosphate can be decarboxylated photochemically to uridine-5′-phosphate^61,62^, which is an extant nucleotide of the current RNA. Orotidine would likely be replaced by uridine and cytidine due to their higher functionality capacities^51^.

Therefore, the results discussed in this work increase our knowledge about the scarcely studied prebiotic hydrothermal cyanide chemistry^15,16,20,34,63^, further demonstrating its relevance in chemical evolution research in the context of the origins of life on the early Earth.

## Conclusions and outlook

Barbituric acid (**8**), orotic acid (**10**), melamine (**11**) and cyanuric acid (**14**) could have played important roles as noncanonical nucleobases in a scenario of chemical evolution towards the generation of the RNA world. The plausible prebiotic synthesis of these four specific N-heterocycles using cyanide as a prebiotic precursor was shown in a conceivable and reasonable geological hydrothermal environment, together with the production of other pyrimidines and triazines, such as 5-amino uracil (**5**), 6-amino uracil (**6**), ammeline (**12**), and ammelide (**13**), which are compatible with an extant nucleobase in a Watson–Crick-like base pair. The production of triazines would be preferential under aerial hydrothermal conditions, but subaerial environments would favour the generation of pyrimidines in the context of the early Earth. The percolation processes and the hydrothermal convective flows would bring into contact both sets of bases. The wet–dry cycles in the aerial part of the alkaline system would provide adequate conditions for the concentration of cyanide, from the atmosphere and from the subaerial part of the system, as well as the formation of supramolecular aggregates. The only constraint of this plausible prebiotic scenario is the relative constancy of the pH throughout the hydrothermal system because cyanide can only undergo polymerization at pH = 8–10 and can only be concentrated by evaporation at alkaline pH, and most of the N-heterocycles included in this study are sensitive to acidic hydrolysis conditions. In this way, the present work complements previous studies of the possible prebiotic synthesis of triazines from urea^10,35^ and from cyanamide ^64^, showing alternative abiotic pathways for their production.

Therefore, alkaline hydrothermal environments seem to be highly favourable niches for the generation of pre-RNA nucleobases. Taking into account the analytical results presented here, it is important to understand because the production of pyrimidines and triazines is highly determinated by to the experimental conditions and also the role of the minerals in these type of alkaline enviroments^61^. These factors provide interesting topics for future research.

## Supplementary Information

Supplementary Information.

## Data Availability

All data generated or analysed during this study are included in this published article and its supplementary information files. Request for more details to the corresponding author.
