# Supplementary material for: Prebiotic synthesis of noncanonical nucleobases under plausible alkaline hydrothermal conditions
Source: Sci Rep. 2022 Sep 7;12:15140. doi: 10.1038/s41598-022-19474-0 (PMC9452575; doi:10.1038/s41598-022-19474-0)
Supplement: Supplementary file 1 — Supplementary Information. [file 41598_2022_19474_MOESM1_ESM.pdf]

Supplementary Information

PREBIOTIC SYNTHESIS OF NONCANONICAL NUCLEOBASES UNDER PLAUSIBLE ALKALINE HYDROTHERMAL CONDITIONS

Cristina Pérez-Fernández, Jorge Vega, Pedro Rayo-Pizarroso, Eva Mateo-Martí, Marta Ruiz-Bermejo

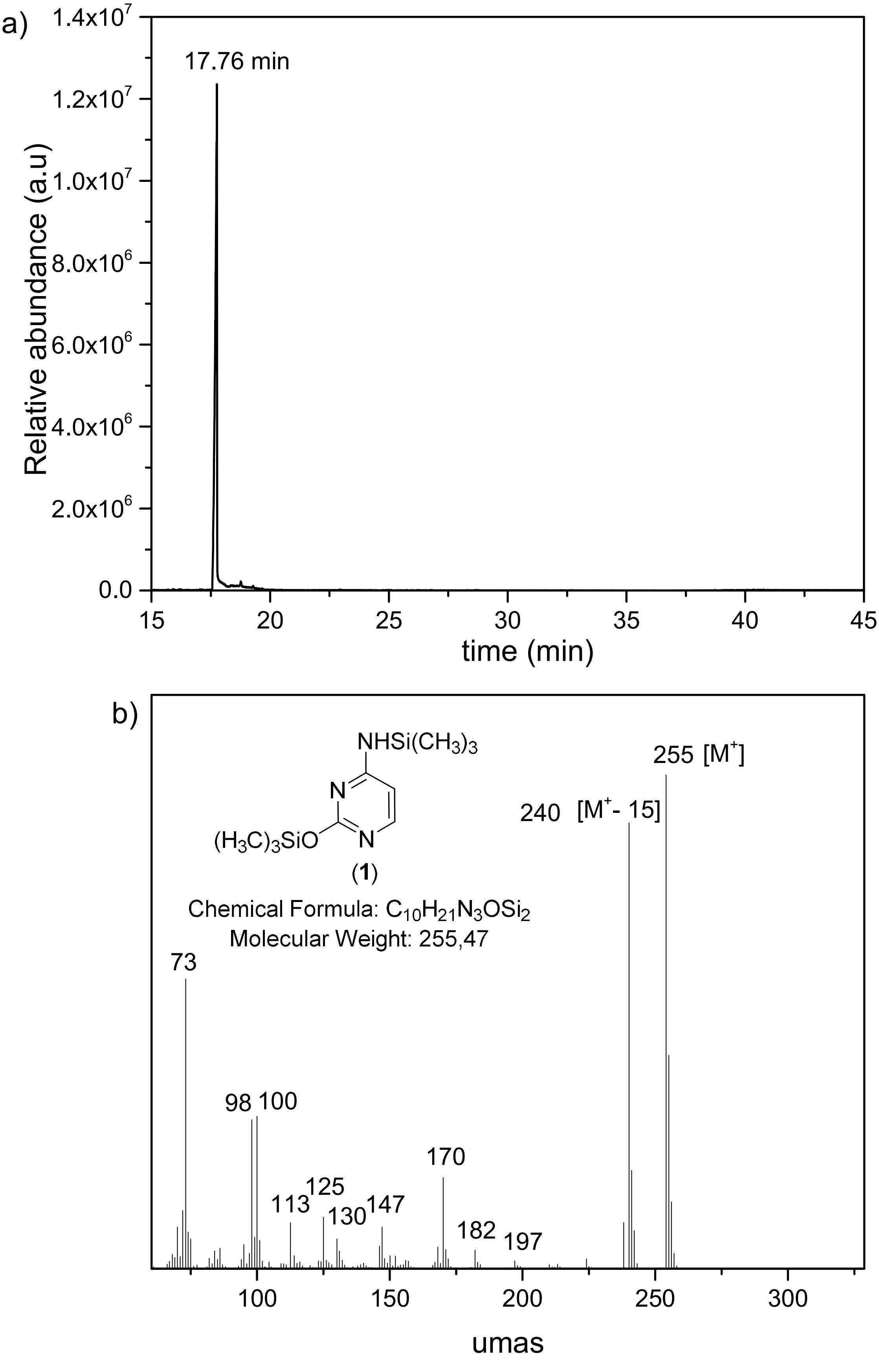

Figure S1. a) GC-MS chromatogram of a standard of cytosine (**1**) derivatized with BSTFA; b) Mass spectrum of the TMS-derivative of an autenthic standar of cystosine.
The standard of cytosine was purchased from Sigma-Aldrich. CAS number: 71-30-7

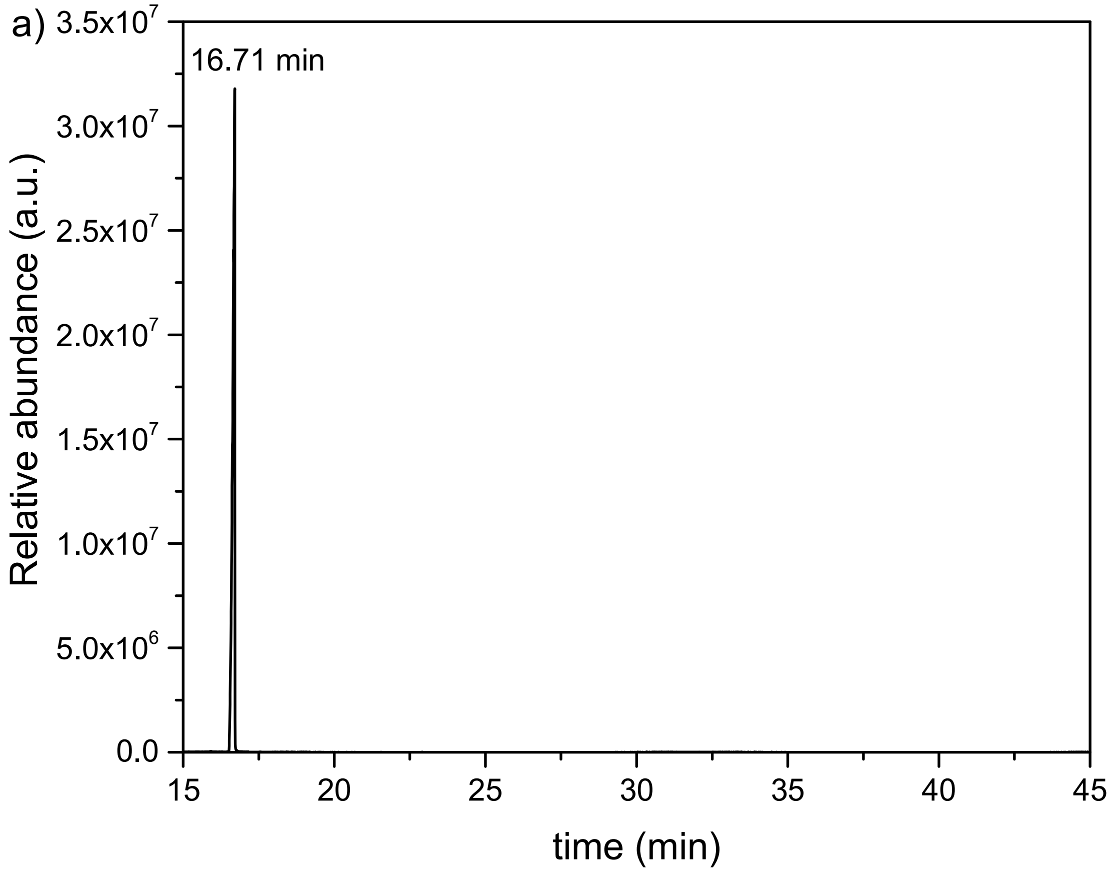

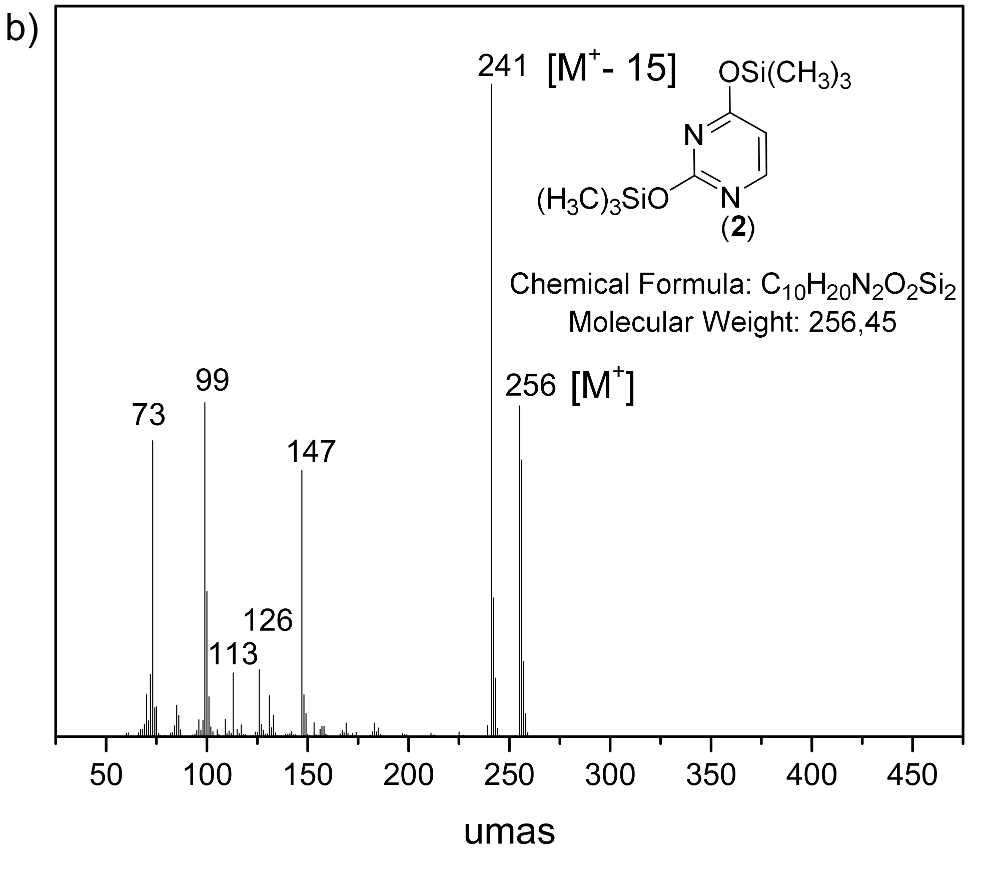

Figure S2. a) GC-MS chromatogram of a standard of uracil (**2**) derivatized with BSTFA; b) Mass spectrum of the TMS-derivative of an authentic standard of uracil.
The standard of uracil was purchased from Sigma-Aldrich. CAS number: 66-22-8

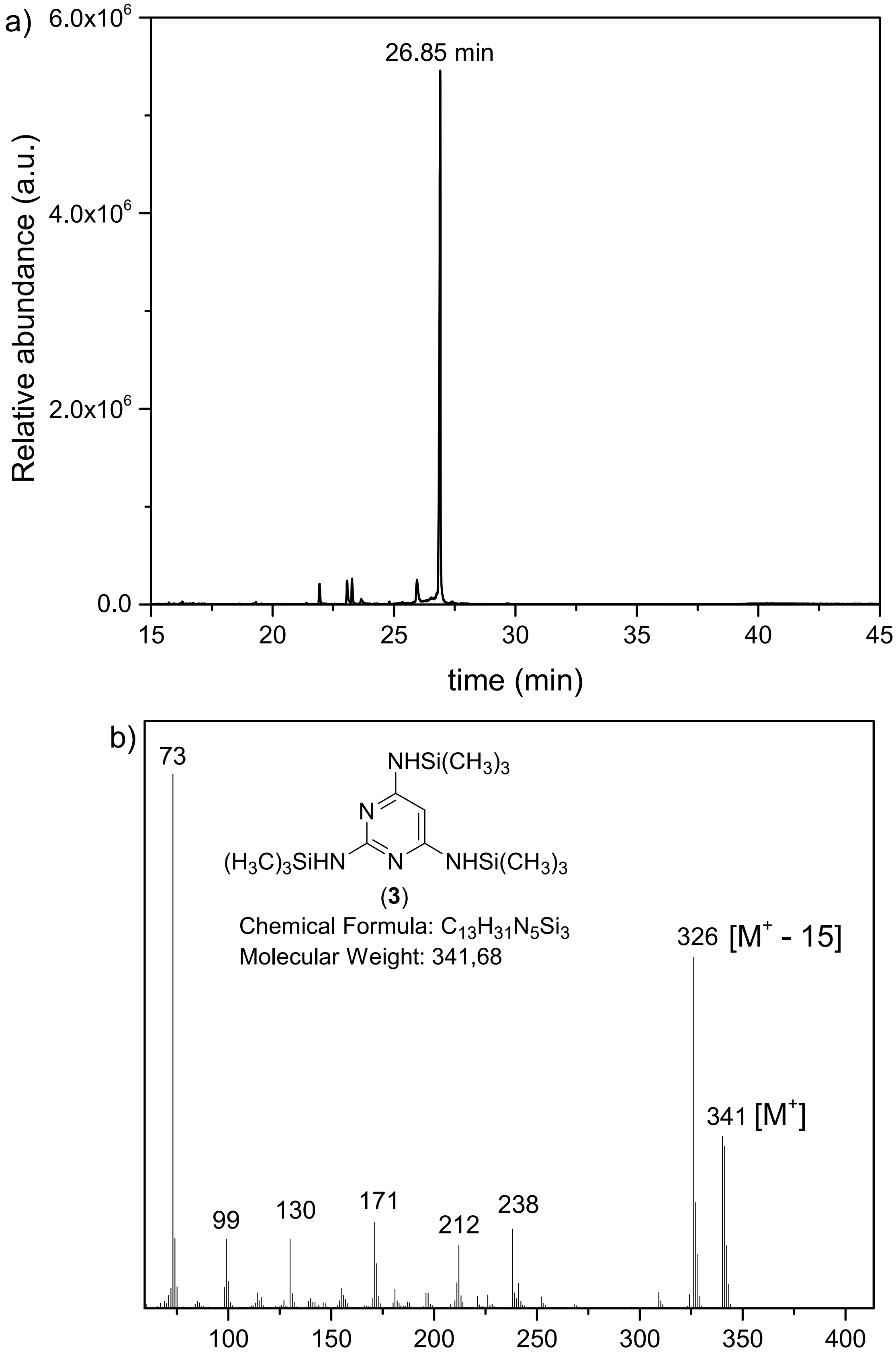

Figure S3. a) GC-MS chromatogram of a standard of pyrimidine-2,4,6-triamine (TAP) (**3**) derivatized with BSTFA; b) Mass spectrum of the TMS-derivative of an authentic standard of pyrimidine-2,4,6-triamine (TAP) (**3**).

The standard of pyrimidine-2,4,6-triamine (TAP) was purchased from Acros Organics. CAS number: 1004-38-2

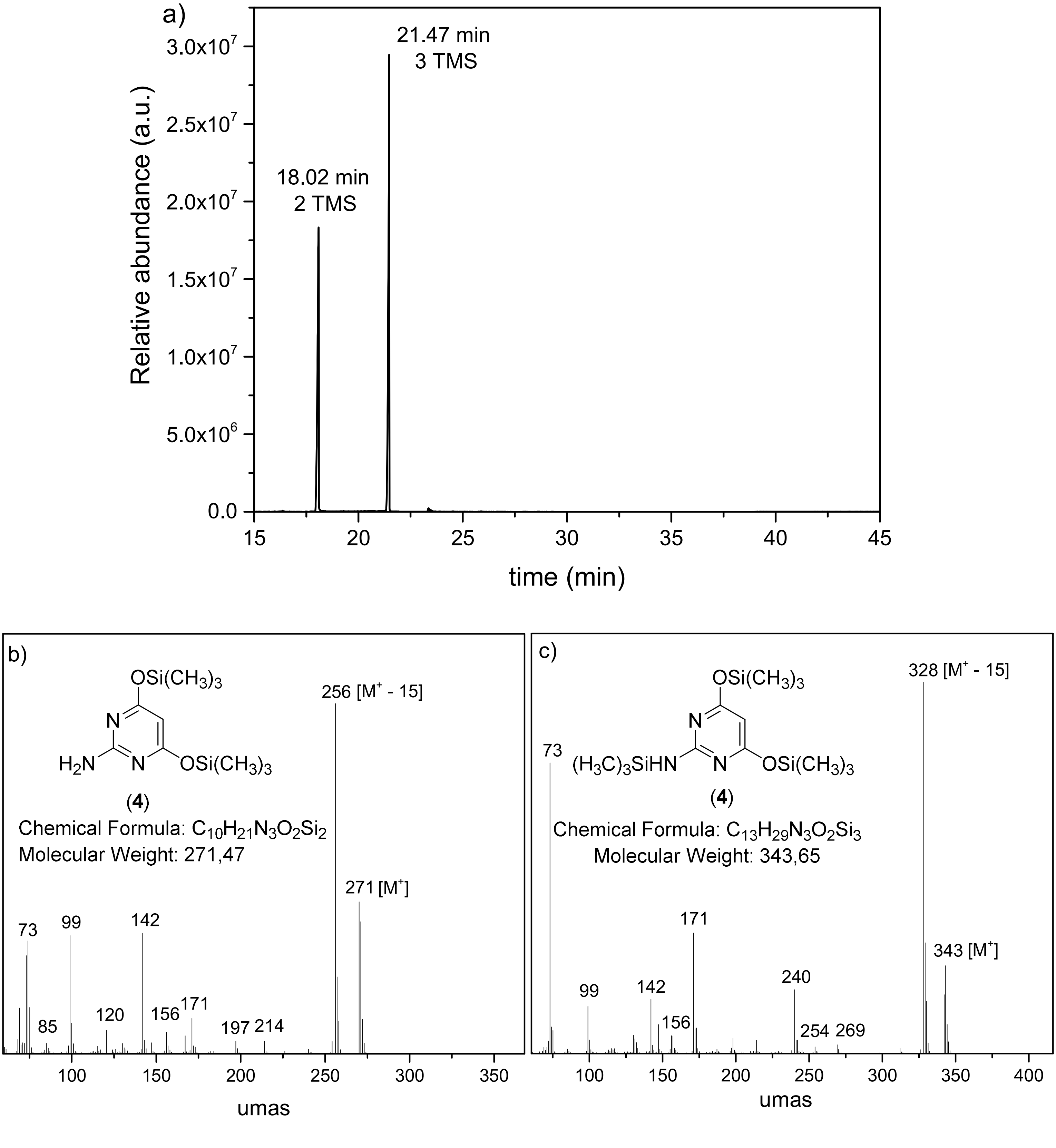

Figure S4. a) GC-MS chromatogram of a standard of 2-aminopyrimidine-4,6-diol (**4**) derivatized with BSTFA; b) and c) Mass spectra of the TMS-derivatives (2 TMS, 18.02 min and 3 TMS 21.47 min) of an authentic standard of 2-aminopyrimidine-4,6-diol (**4**).

The standard of 2-aminopyrimidine-4,6-diol was purchased from Alfa Aesar. CAS number: 56-09-7

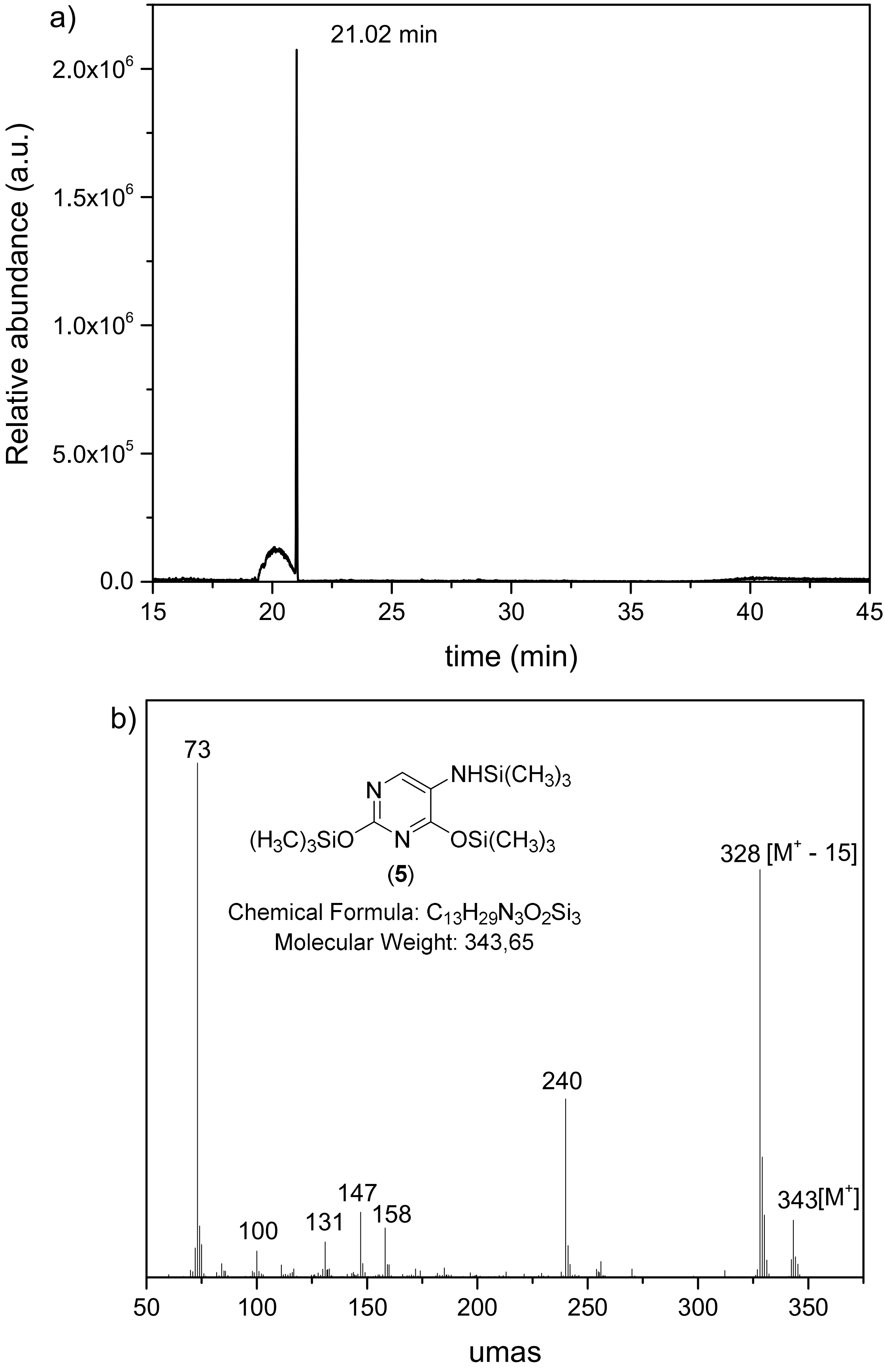

Figure S5. a) GC-MS chromatogram of a standard of 5-aminouracil (**5**) derivatized with BSTFA; b) Mass spectrum of the TMS-derivative of an authentic standard of 5-aminouracil (**5**).
The standard of 5-aminouracil was purchased from Sigma-Aldrich. CAS number: 932-52-5

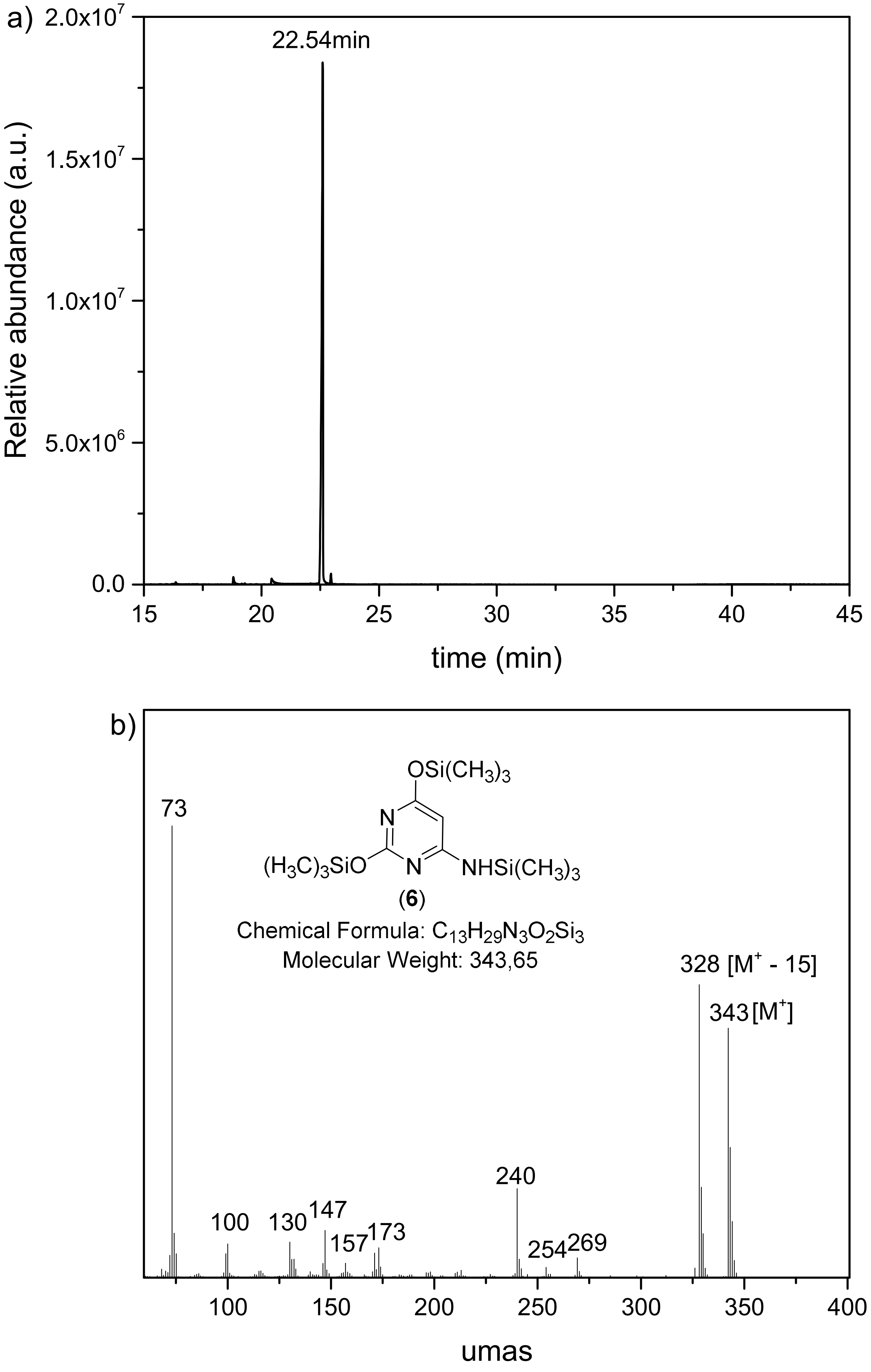

Figure S6. a) GC-MS chromatogram of a standard of 6-aminouracil (**6**) derivatized with BSTFA; b) Mass spectrum of the TMS-derivative of an authentic standard of 6-aminouracil (**6**).

The standard of 6-aminouracil was purchased from Alfa Aesar. CAS number: 873-83-6

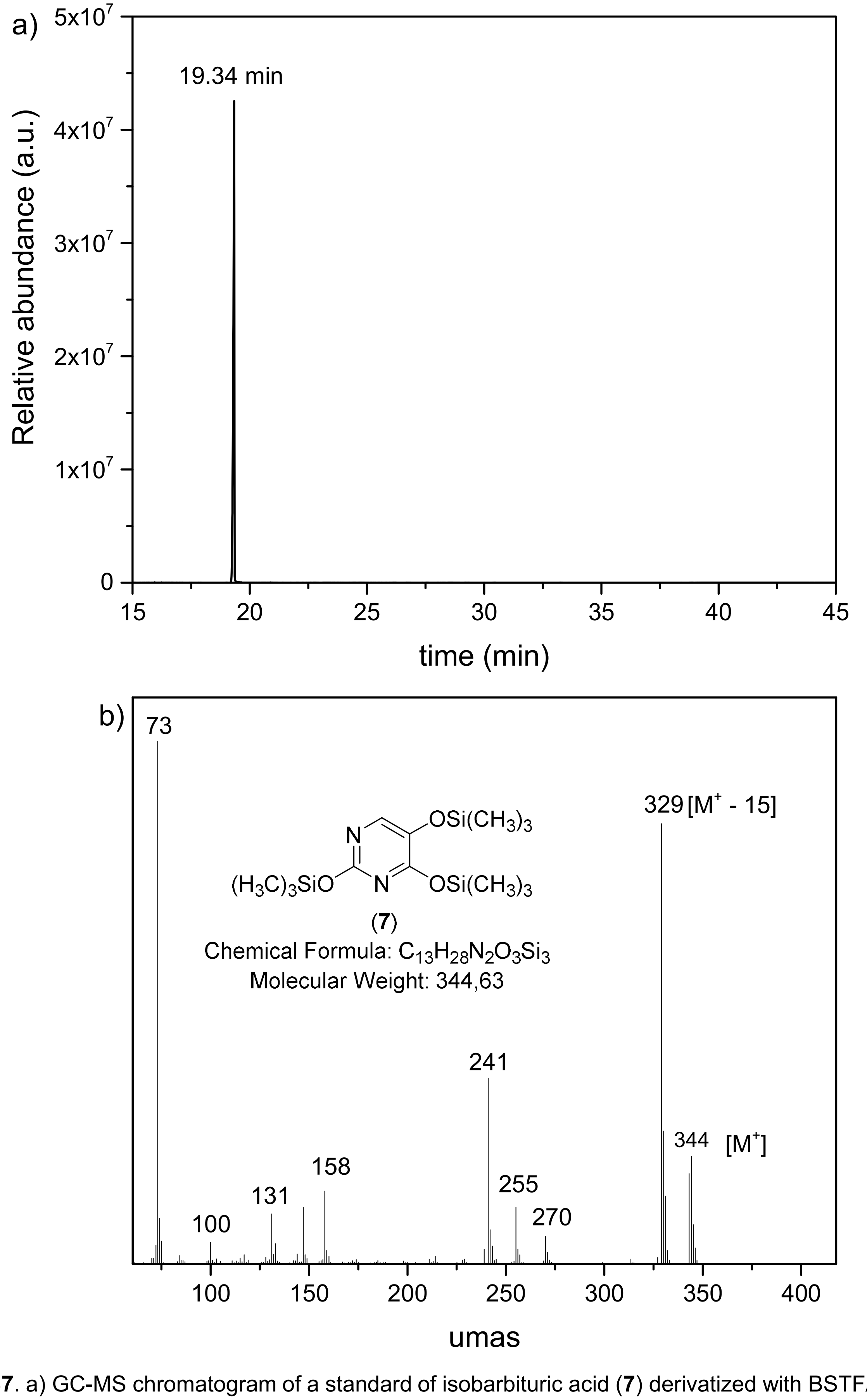

Figure S7. a) GC-MS chromatogram of a standard of isobarbituric acid (**7**) derivatized with BSTFA; b) Mass spectrum of the TMS-derivative of an authentic standar of isobarbituric acid (**7**).
The standard of isobarbituric acid was purchased from Sigma-Aldrich. CAS number: 496-76-4

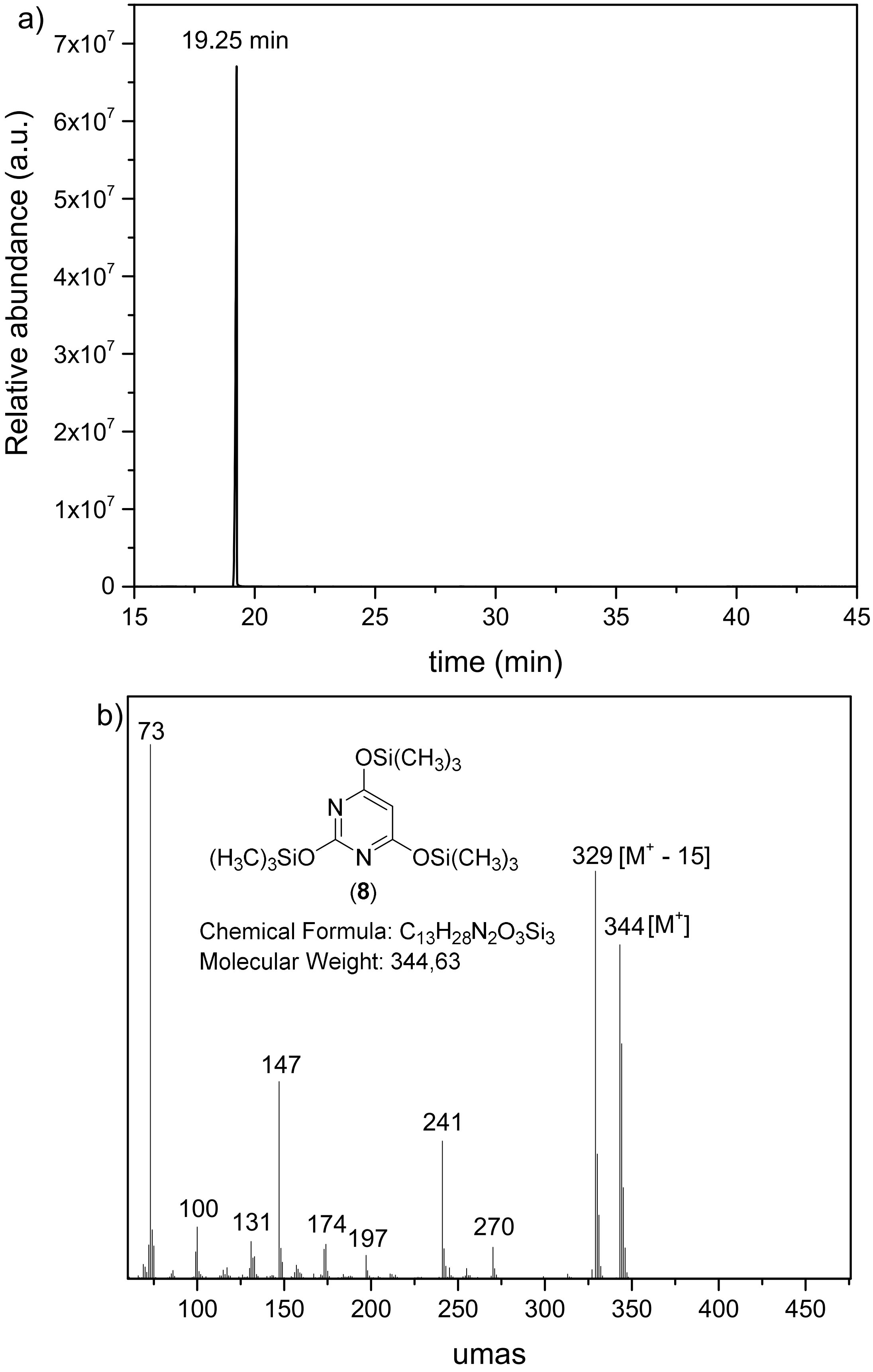

Figure S8. a) GC-MS chromatogram of a standard of barbituric acid (**8**) derivatized with BSTFA; b) Mass spectrum of the TMS-derivative of an authentic standard of barbituric acid (**8**).
The standard of barbituric acid was purchased from Alfa Aesar. CAS number: 67-52-7

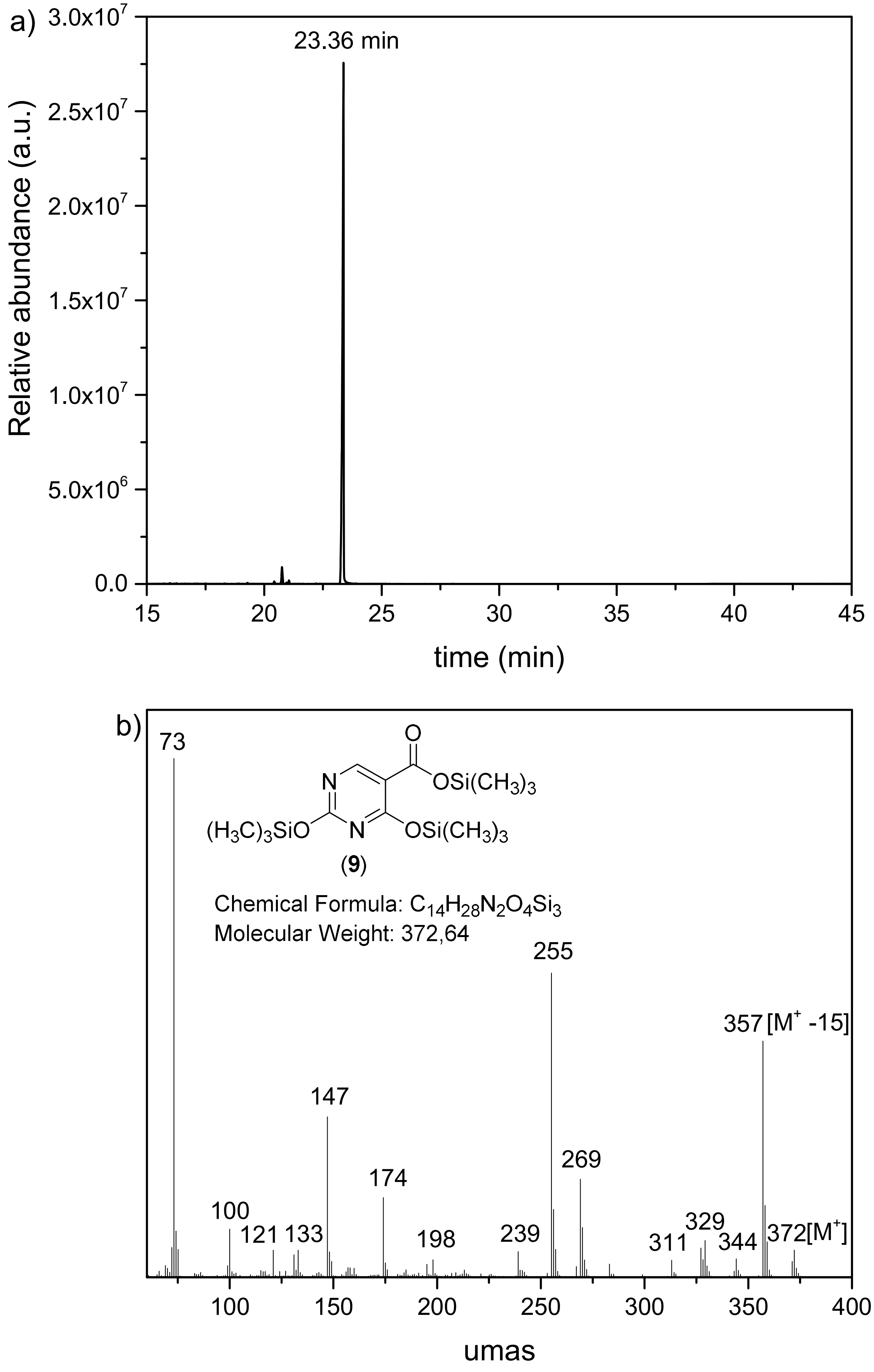

Figure S9. a) GC-MS chromatogram of a standadr of uracil-5-carboxylic acid (**9**) derivatized with BSTFA; b) Mass spectrum of the TMS-derivative of an authentic standard of uracil-5-carboxylic acid (**9**).
The standard of uracil-5-carboxylic acid was purchased from Acros Organics. CAS number: 23945-44-0

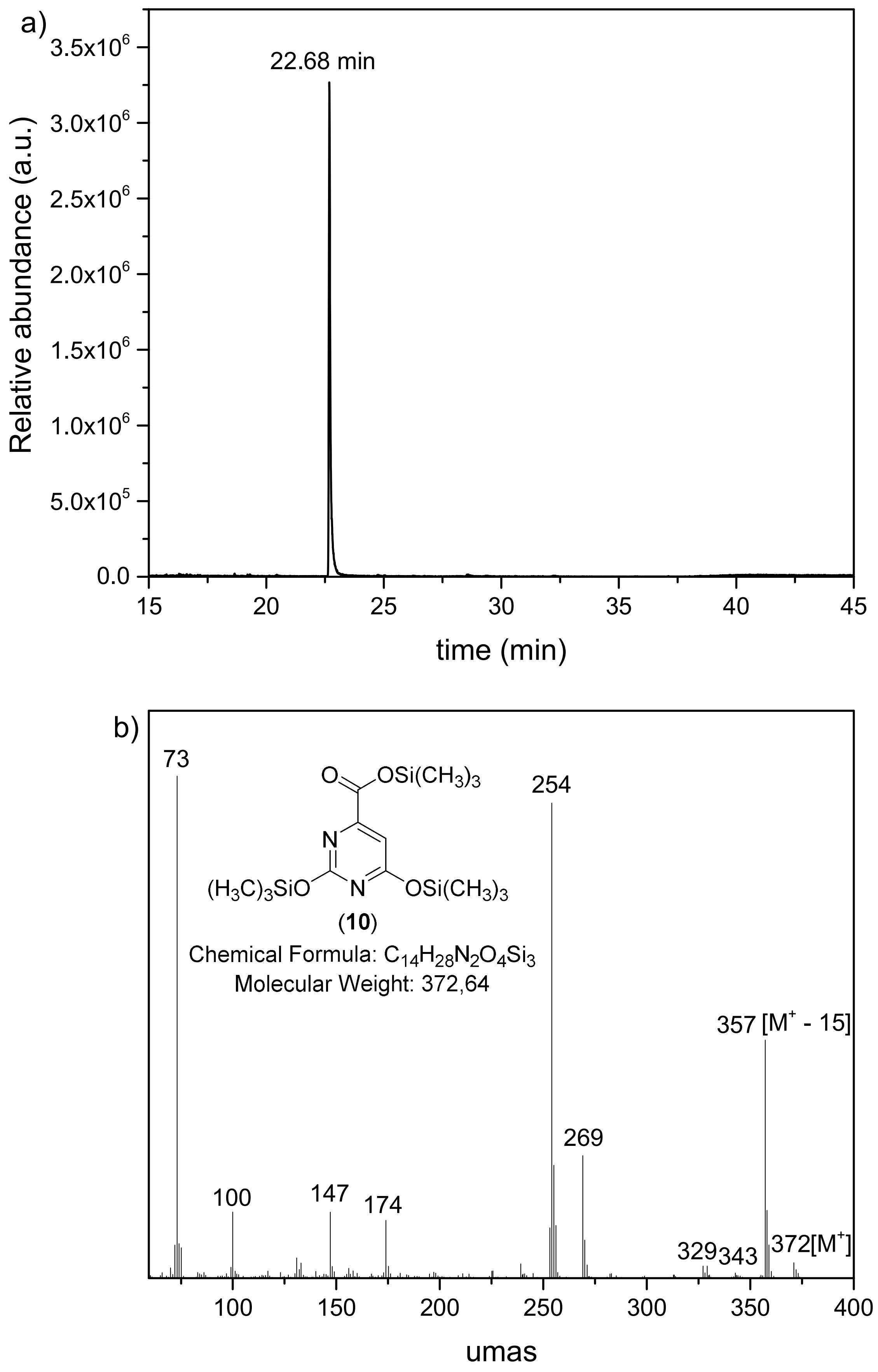

Figure S10. a) GC-MS chromatogram of a standadr of orotic acid (**10**) derivatized with BSTFA; b) Mass spectrum of the TMS-derivative of an authentic standard of orotic acid (**10**).
The standard of orotic acid was purchased from Sigma-Aldrich. CAS number: 50887-69-9

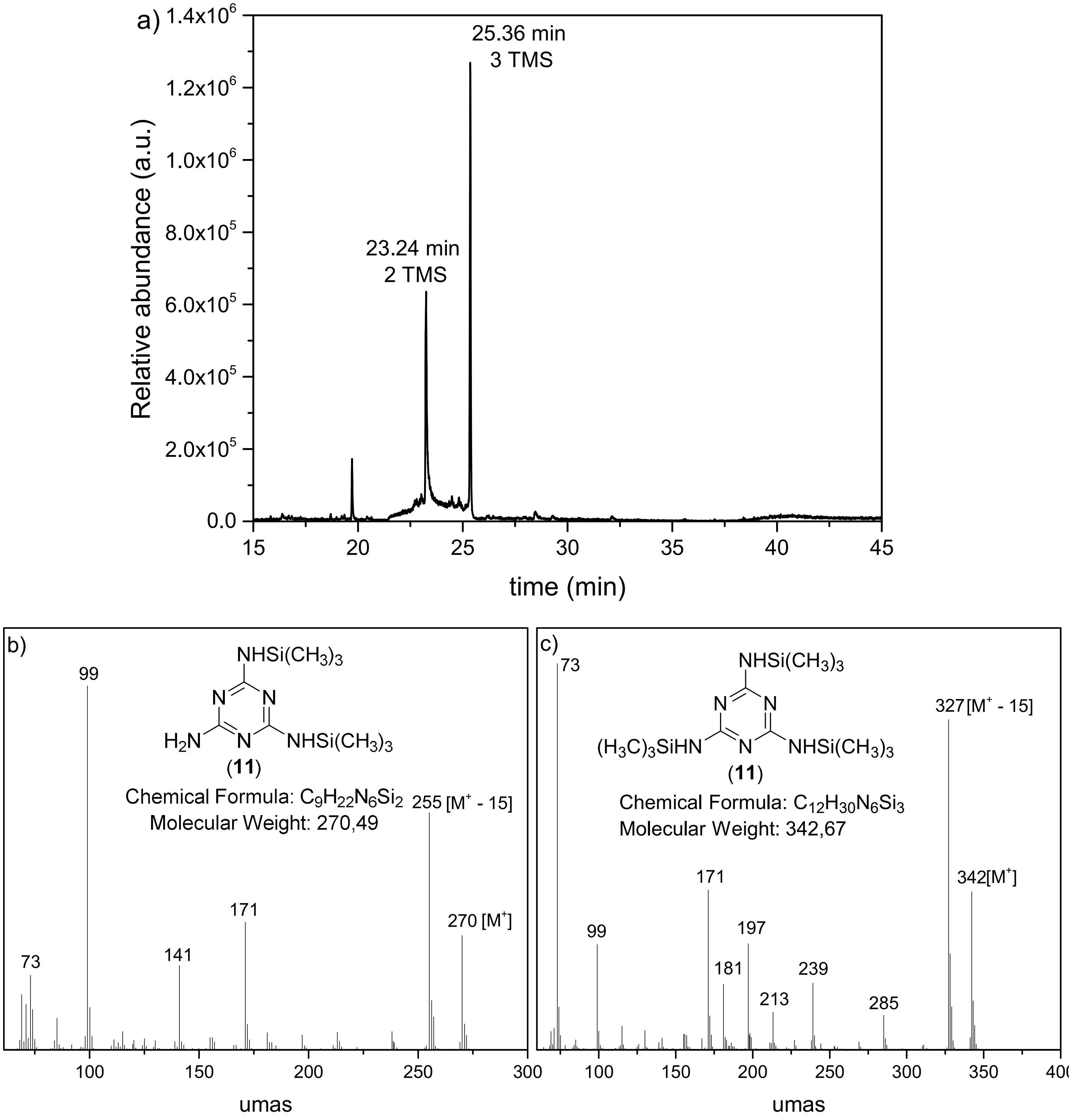

Figure S11. a) GC-MS chromatogram of a standard of melamine (**11**) derivatized with BSTFA; b) and c) Mass spectra of the TMS-derivatives (2 TMS, 23.24 min and 3 TMS 25.36 min) of an authentic standard of melamine (**11**).

The standard of melamine was purchased from Sigma-Aldrich. CAS number: 108-78-1

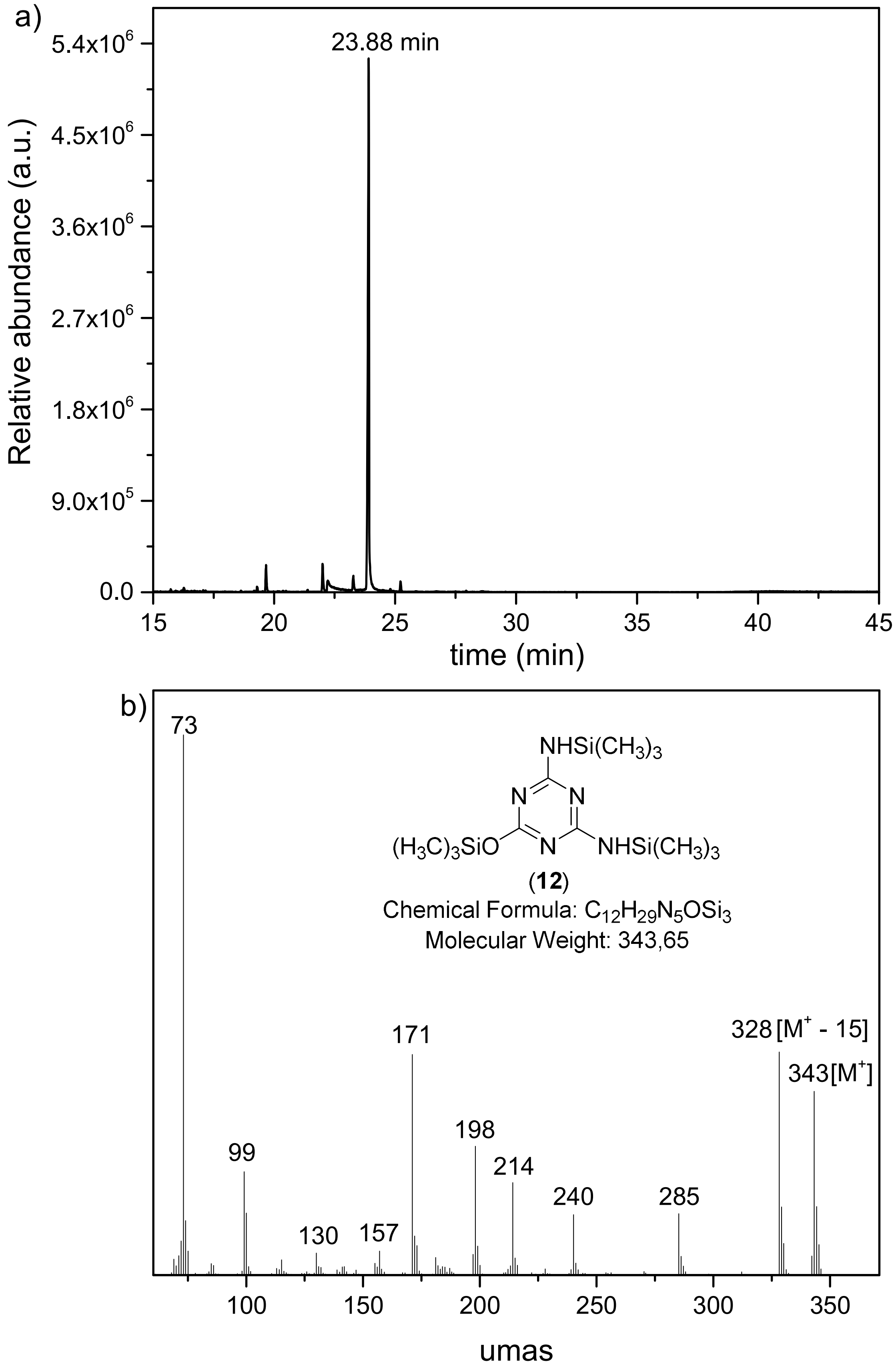

Figure S12. a) GC-MS chromatogram of a standar of ammeline (**12**) derivatized with BSTFA; b) Mass spectrum of the TMS-derivative of an autenthic standar of ammeline (**12**).
The standard of ammeline was purchased from Sigma-Aldrich. CAS number: 645-92-1

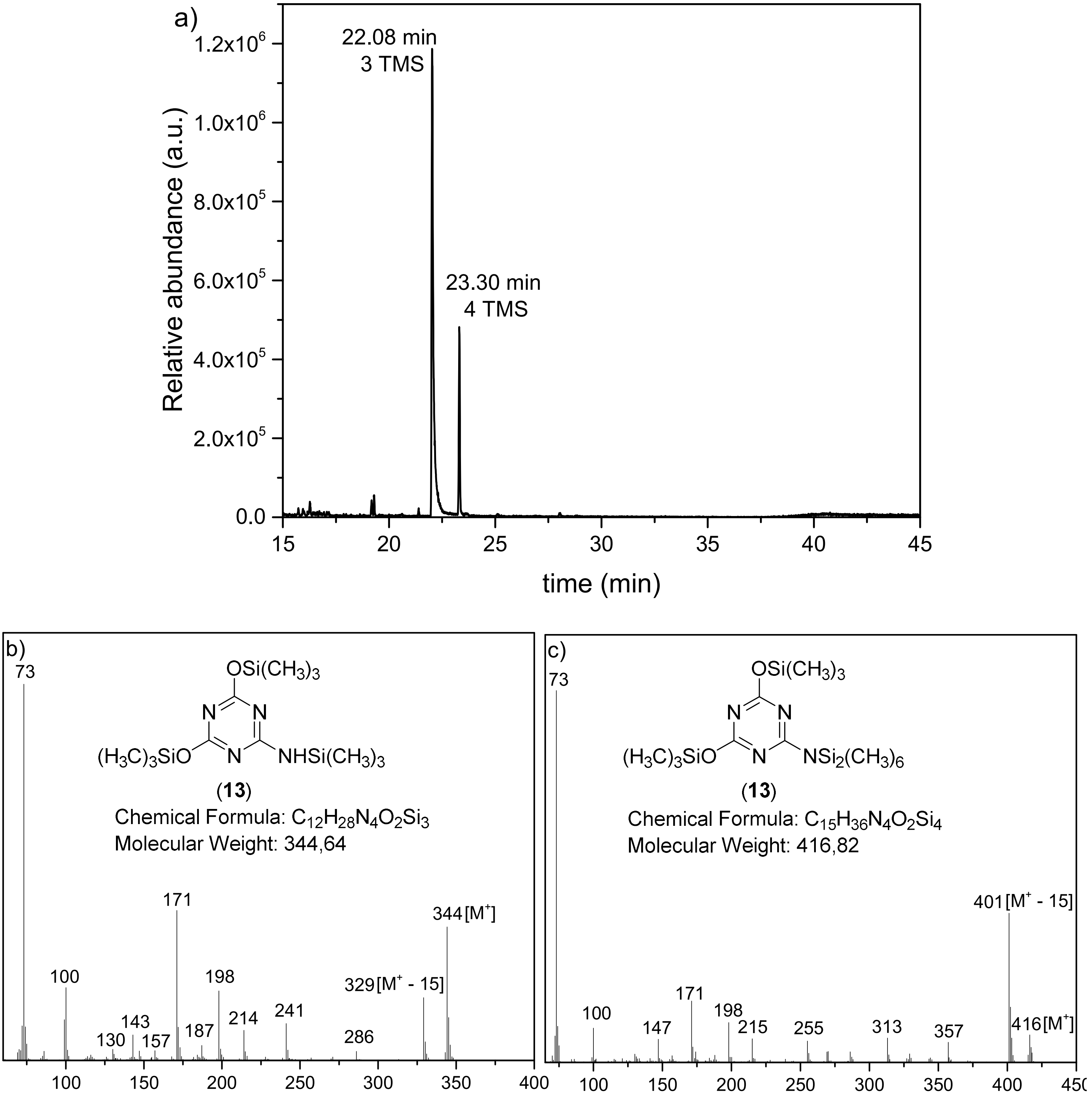

Figure S13. a) GC-MS chromatogram of a standard of ammelide (**13**) derivatized with BSTFA; b) and c) Mass spectra of the TMS-derivatives (3 TMS, 22.08 min and 3 TMS 23.30 min) of an authentic standard of ammelide (**13**).

The standard of ammelide was purchased from TCI. CAS number: 645-93-2

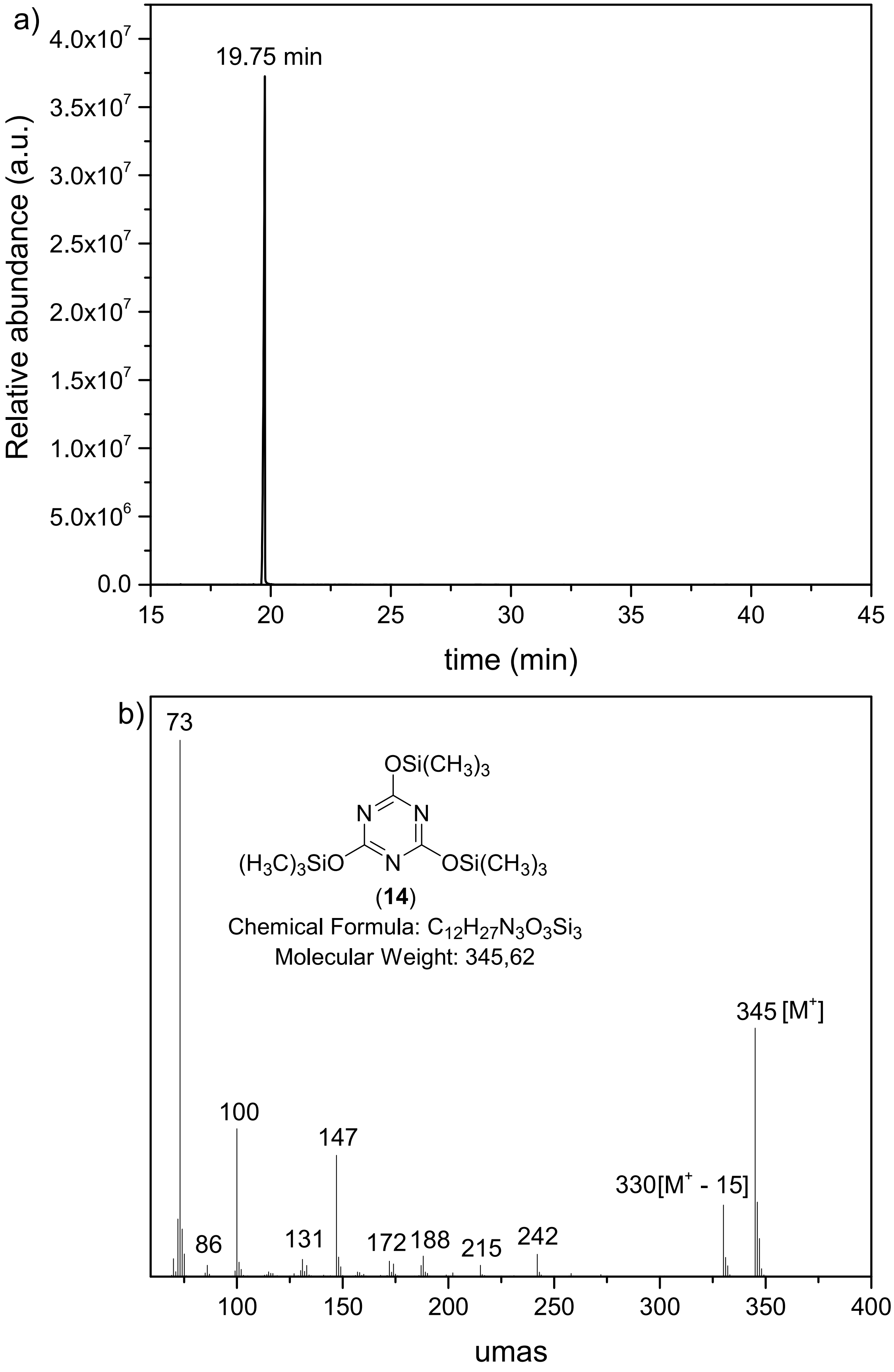

Figure S14. a) GC-MS chromatogram of a standar of cyanuric acid (**14**) derivatized with BSTFA; b) Mass spectrum of the TMS-derivative of an autenthic standar of cyanuric acid (**14**).
The standard of cyanuric acid was purchased from Sigma-Aldrich. CAS number: 108-80-5

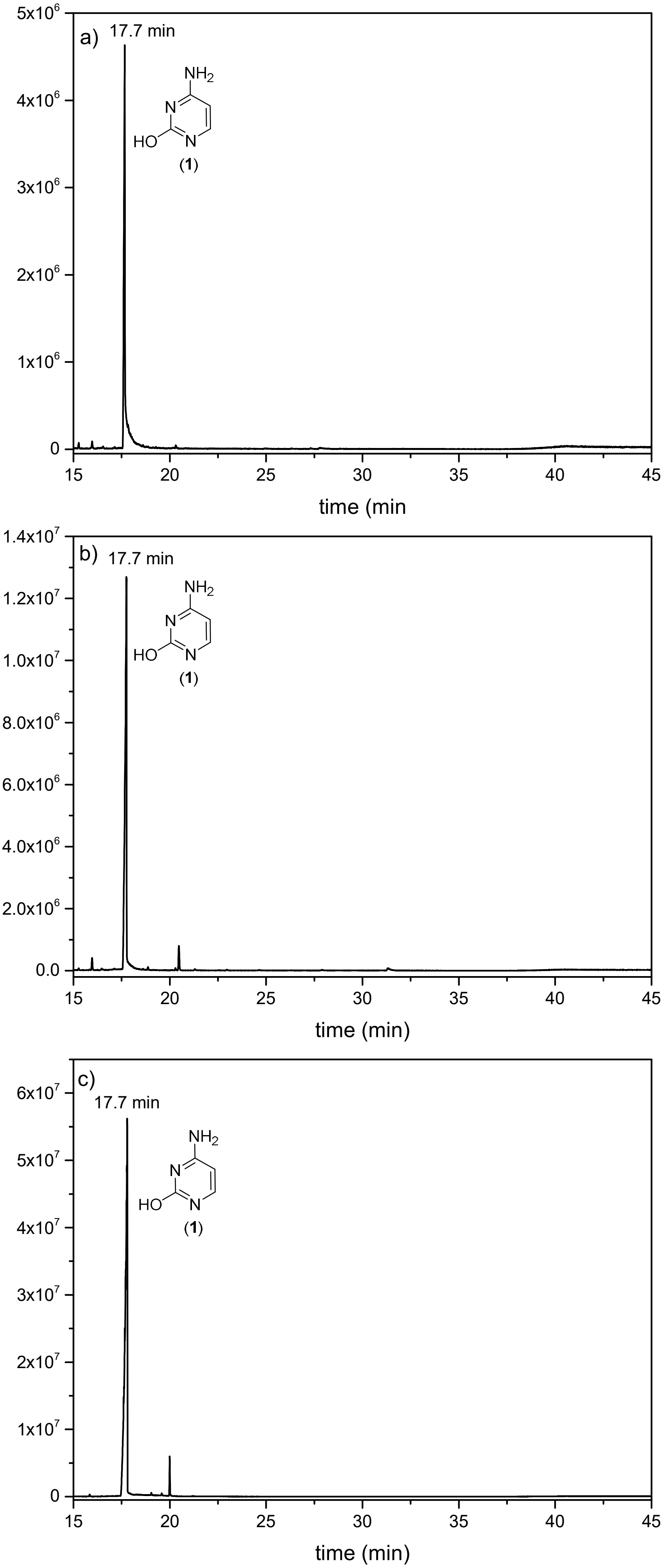

Figure S15. GC-MS chromatograms of a standar of cytosine (**1**) after: a) Acid hydrolysis, 6 N HCl, 110 ºC, 24 h; b) Basic hydrolysis, 5 N NaOH, 110 ºC, 24 h; Neutral hydrolysis, phosphate buffer solution (0,01 M, pH 8.5), 140 ºC, 72 h; and lately derivatization with BSTFA to obtain the corresponding TMS-derivative . From here to the Figure S28, for simplicity, the compounds drawed correspond to the respective underivatized molecule and the tautomer identified. In all cases, from Figure S15 to Figure S28 the hydrolysis conditions were the same.

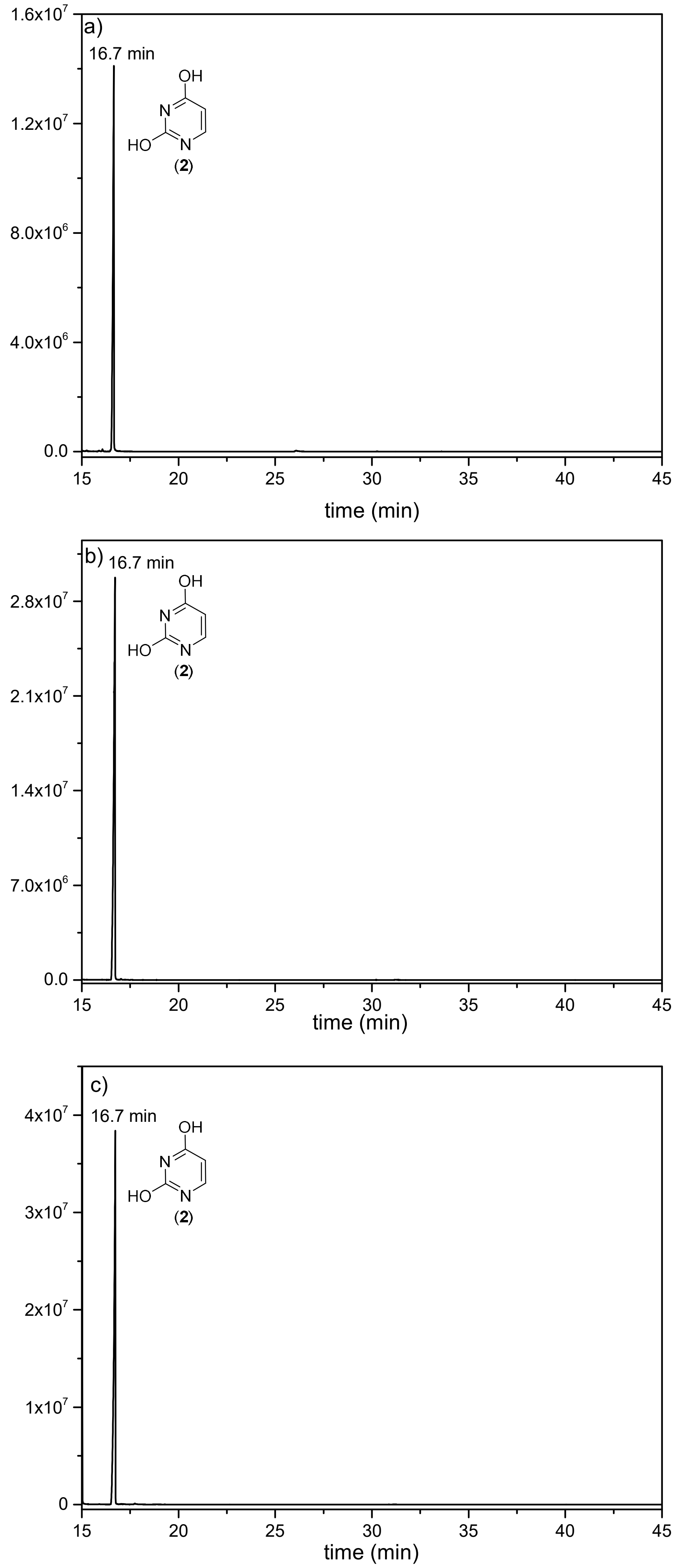

Figure S16. GC-MS chromatograms of a standar of uracil (**2**) after: a) Acid hydrolysis; b) Basic hydrolysis; and c) Neutral hydrolysis; and lately derivatization with BSTFA to obtain the corresponding TMS-derivative .

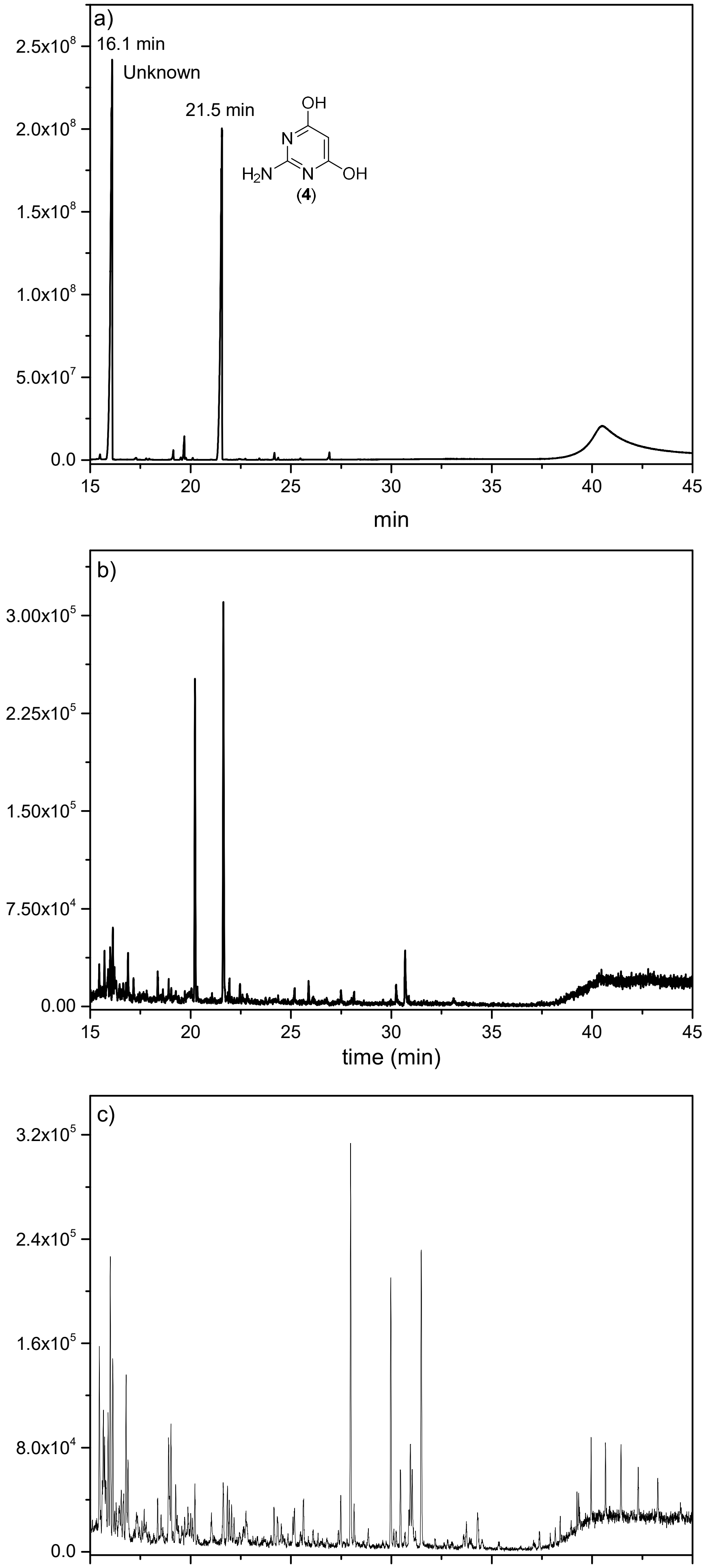

Figure S17. GC-MS chromatograms of a standar of TAP (**3**) after: a) Acid hydrolysis; b) Basic hydrolysis; and c) Neutral hydrolysis; and lately derivatization with BSTFA to obtain the corresponding TMS-derivative . The basic and neutral hydrolysis conditions lead to the total decomposition of TAP. No identified compounds were identified for both cases.

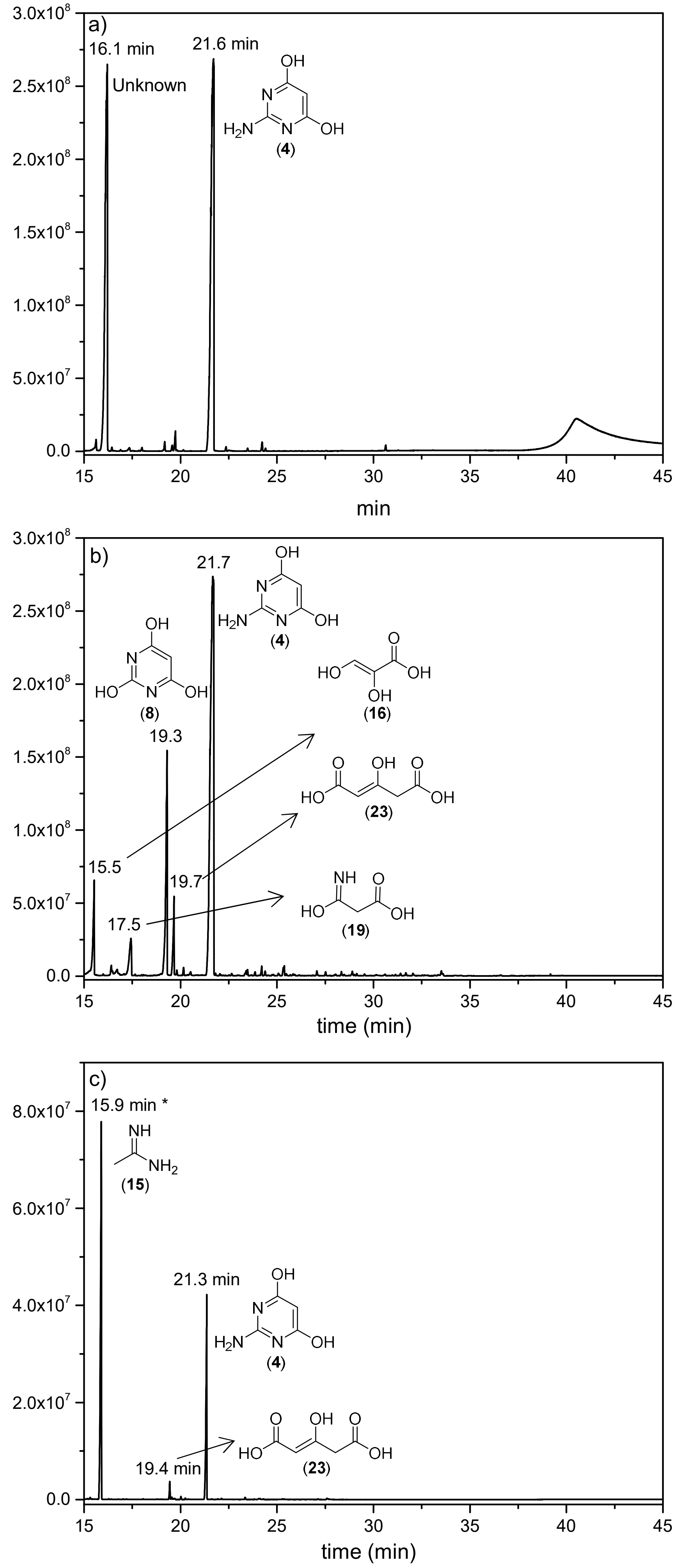

Figure S18. GC-MS chromatograms of a standar of 2-amino-4,6-dihydroxypyrimidine (**4**) after: a) Acid hydrolysis; b) Basic hydrolysis; and c) Neutral hydrolysis; and lately derivatization with BSTFA to obtain the corresponding TMS-derivatives. * Co-elution peak: acetedimidine + Unknown compound

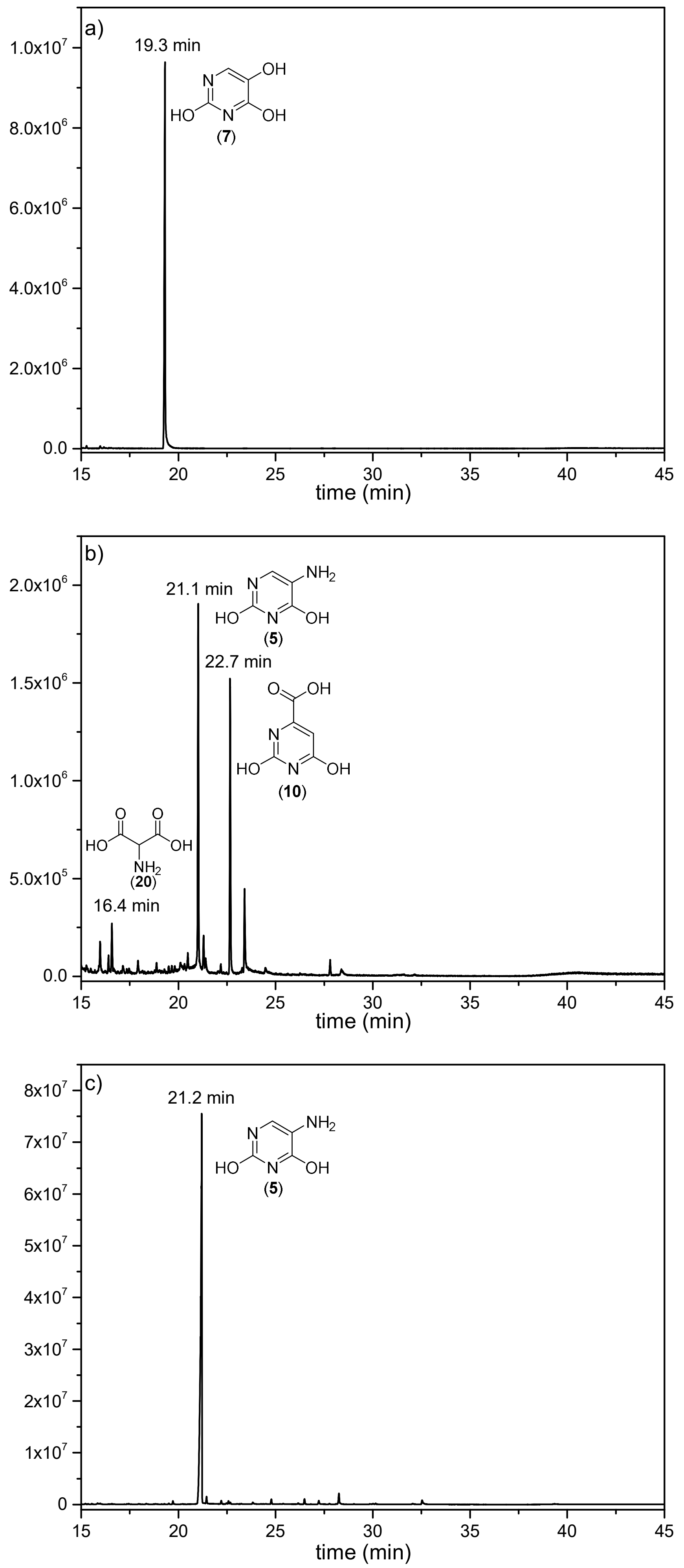

Figure S19. GC-MS chromatograms of a standar of 5-aminouracil (**5**) after: a) Acid hydrolysis; b) Basic hydrolysis; and c) Neutral hydrolysis; and lately derivatization with BSTFA to obtain the corresponding TMS-derivatives.

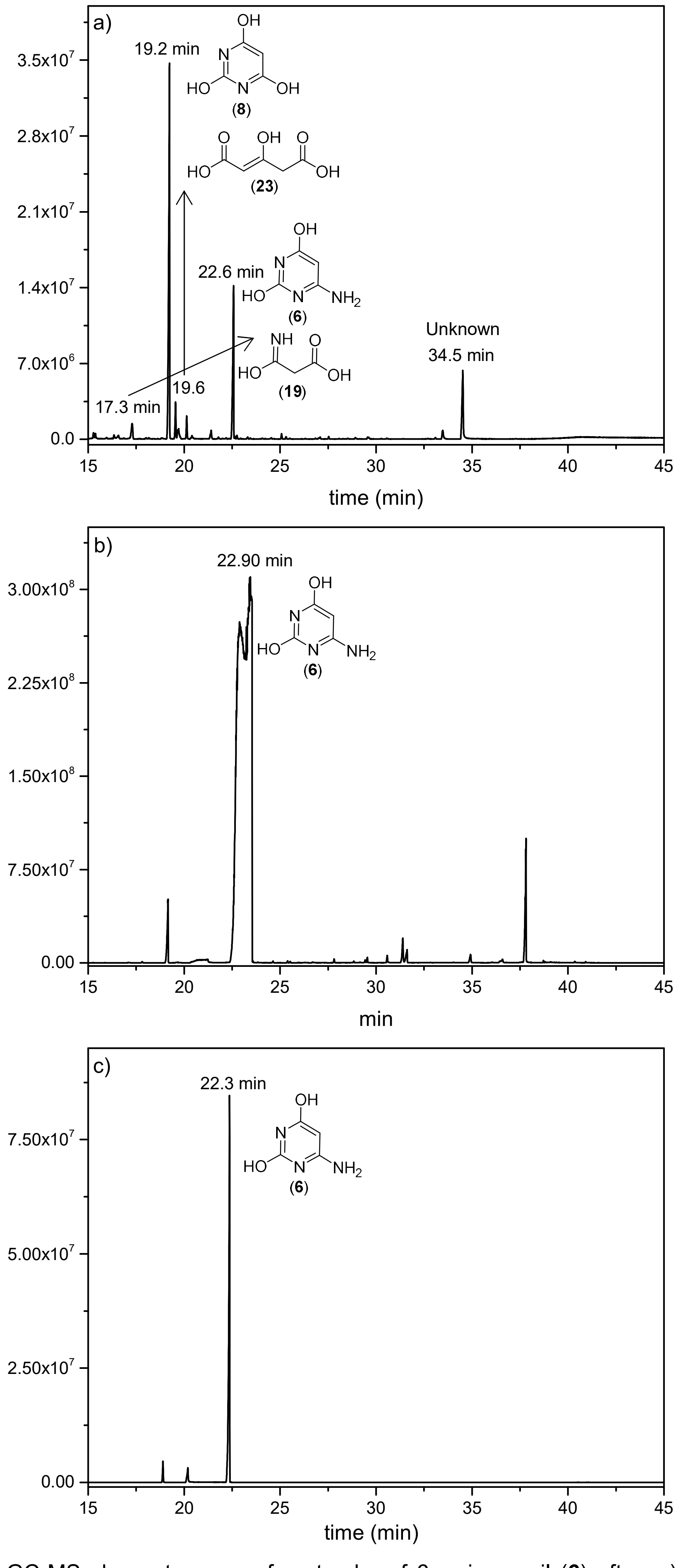

Figure S20. GC-MS chromatograms of a standar of 6-aminouracil (**6**) after: a) Acid hydrolysis; b) Basic hydrolysis; and c) Neutral hydrolysis; and lately derivatization with BSTFA to obtain the corresponding TMS-derivatives.

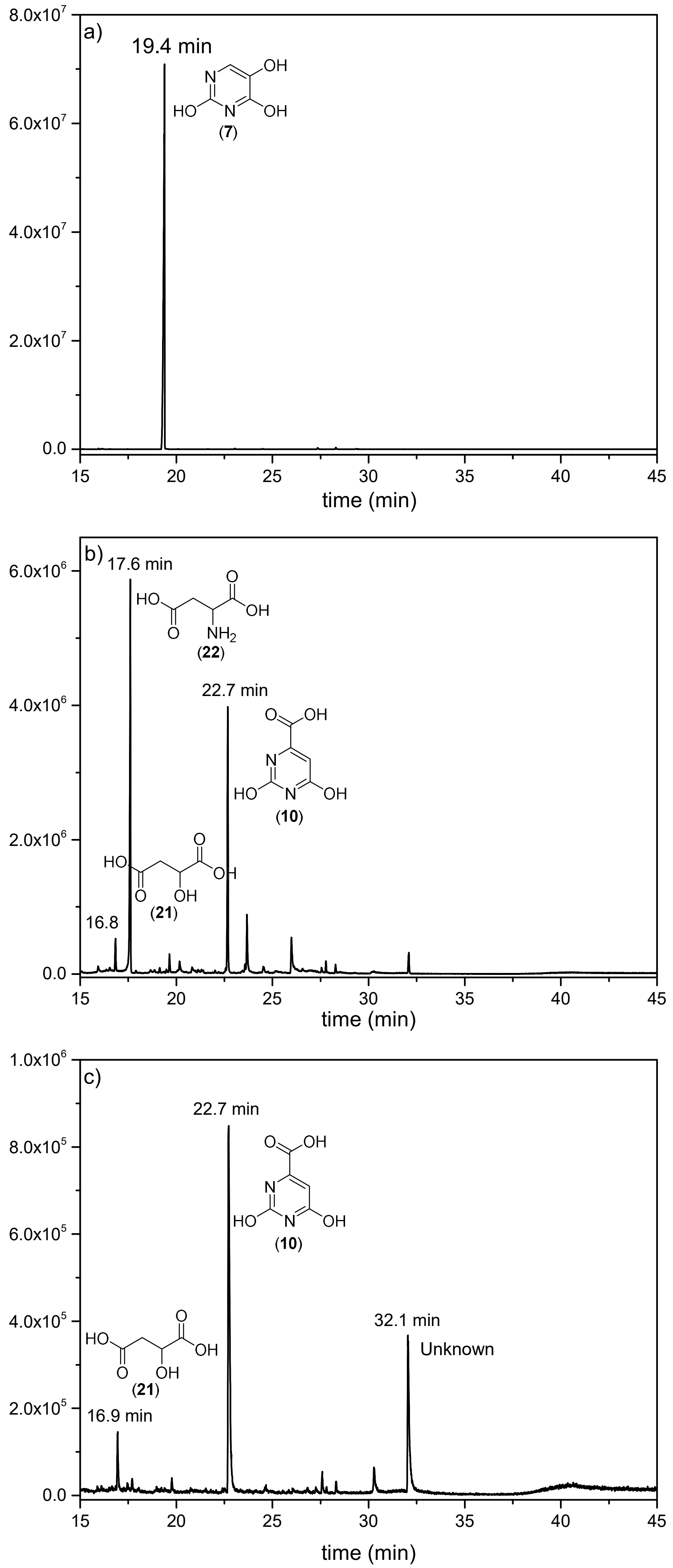

Figure S21. GC-MS chromatograms of a standar of isobarbituric acid (**7**) after: a) Acid hydrolysis; b) Basic hydrolysis; and c) Neutral hydrolysis; and lately derivatization with BSTFA to obtain the corresponding TMS-derivatives.

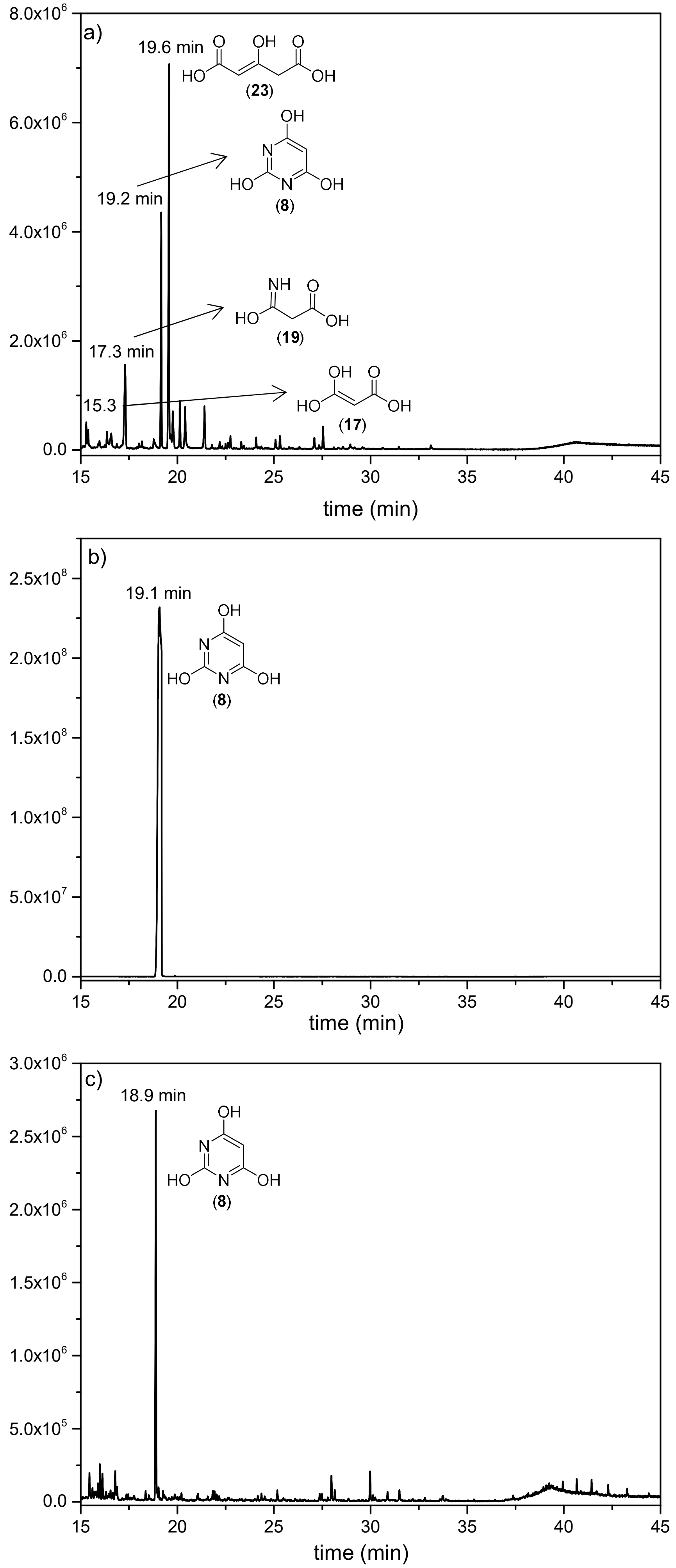

Figure S22. GC-MS chromatograms of a standar of barbituric acid (**8**) after: a) Acid hydrolysis; b) Basic hydrolysis; and c) Neutral hydrolysis; and lately derivatization with BSTFA to obtain the corresponding TMS-derivatives.

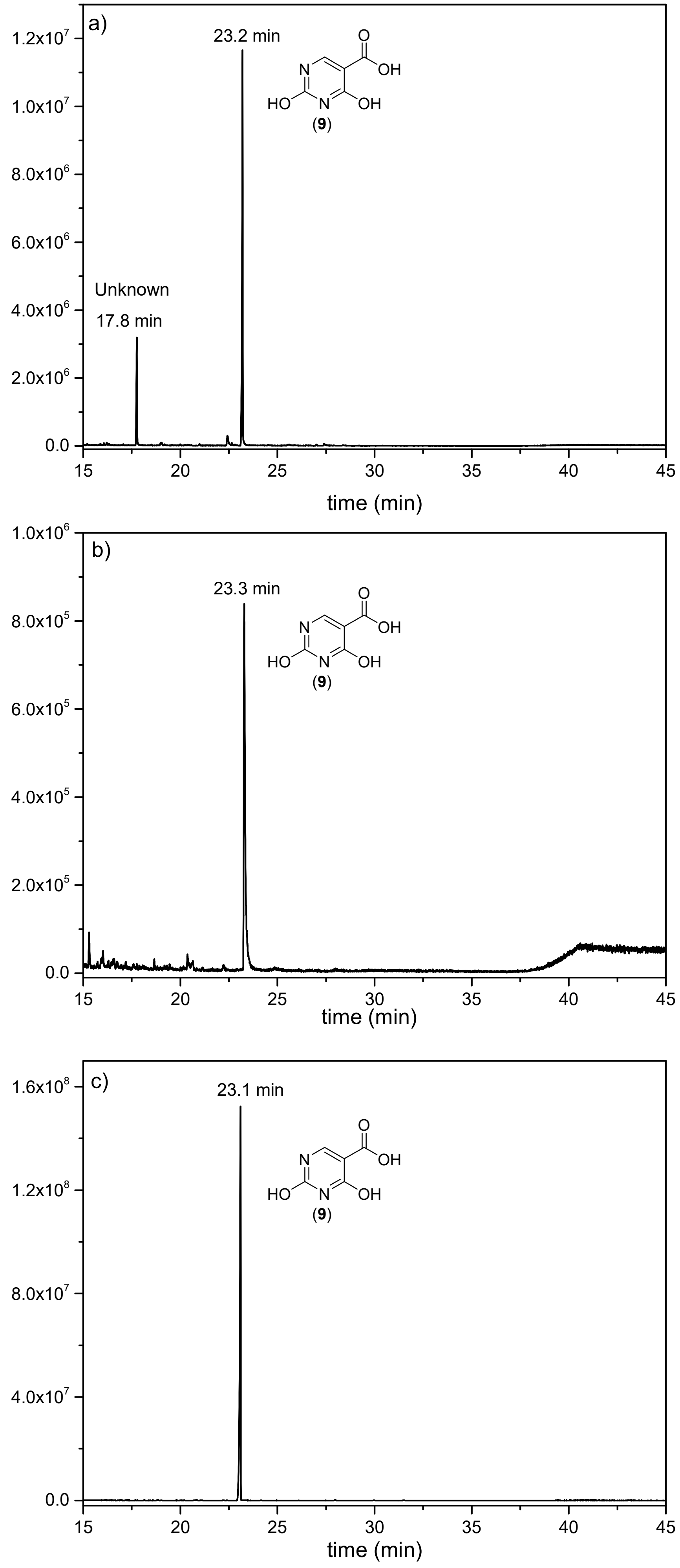

Figure S23. GC-MS chromatograms of a standar of uracil-5-carboxylic acid (**9**) after: a) Acid hydrolysis; b) Basic hydrolysis; and c) Neutral hydrolysis; and lately derivatization with BSTFA to obtain the corresponding TMS-derivatives.

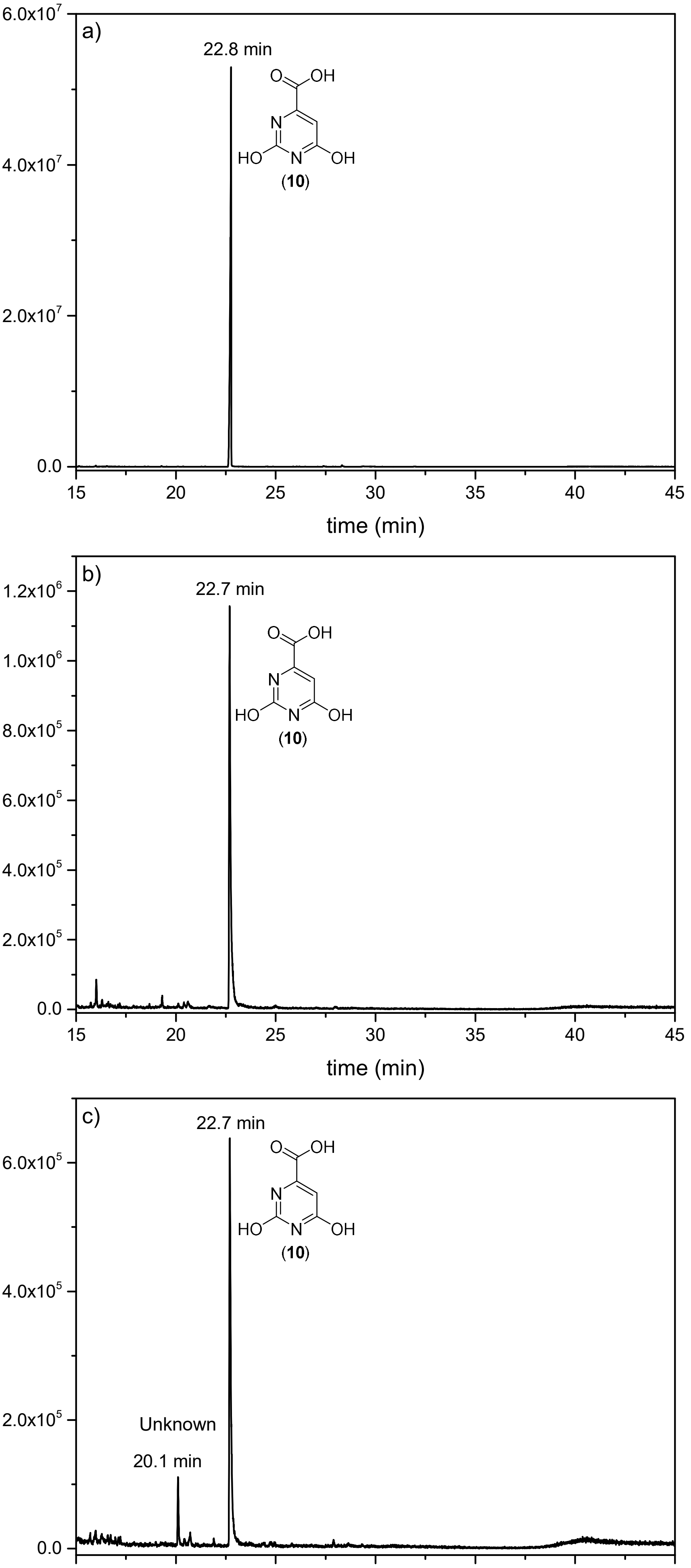

Figure S24. GC-MS chromatograms of a standar of orotic acid (**10**) after: a) Acid hydrolysis; b) Basic hydrolysis; and c) Neutral hydrolysis; and lately derivatization with BSTFA to obtain the corresponding TMS-derivatives.

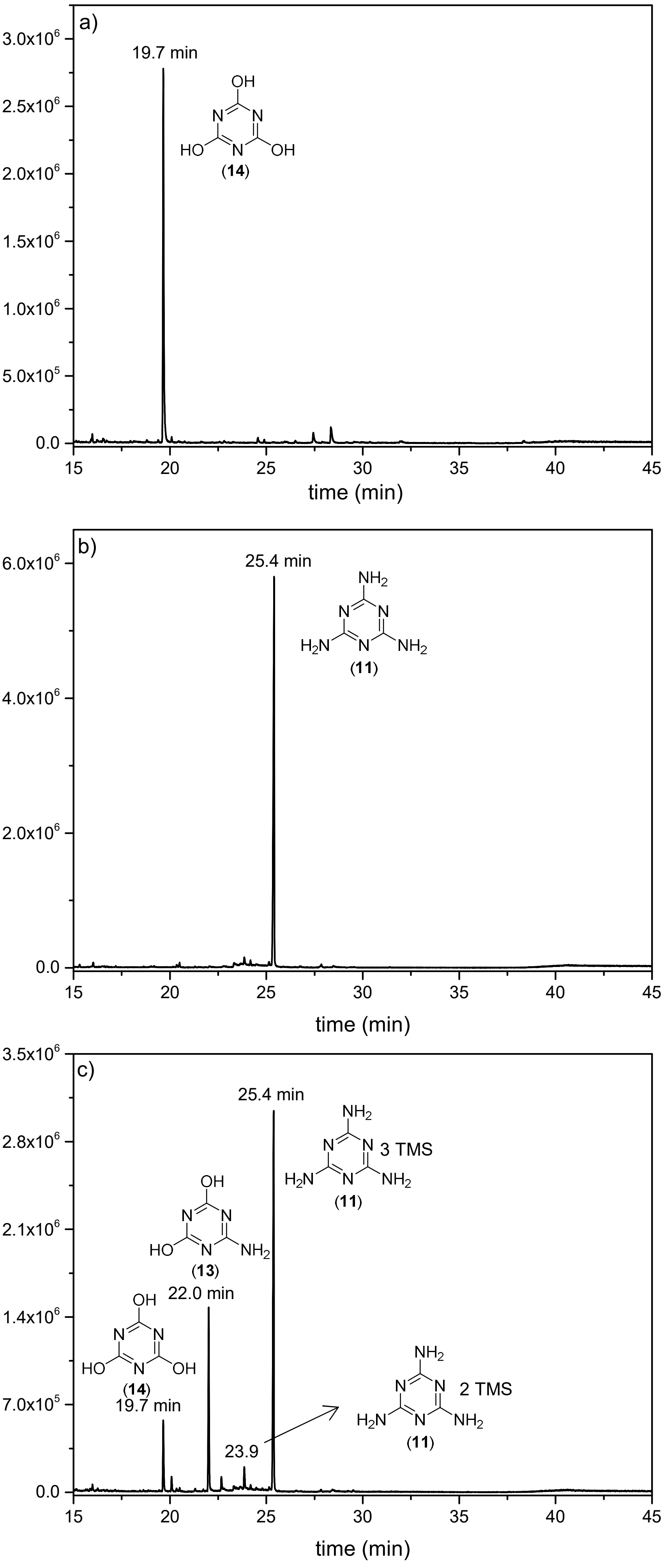

Figure S25. GC-MS chromatograms of a standar of melamine (**11**) after: a) Acid hydrolysis; b) Basic hydrolysis; and c) Neutral hydrolysis; and lately derivatization with BSTFA to obtain the corresponding TMS-derivatives.

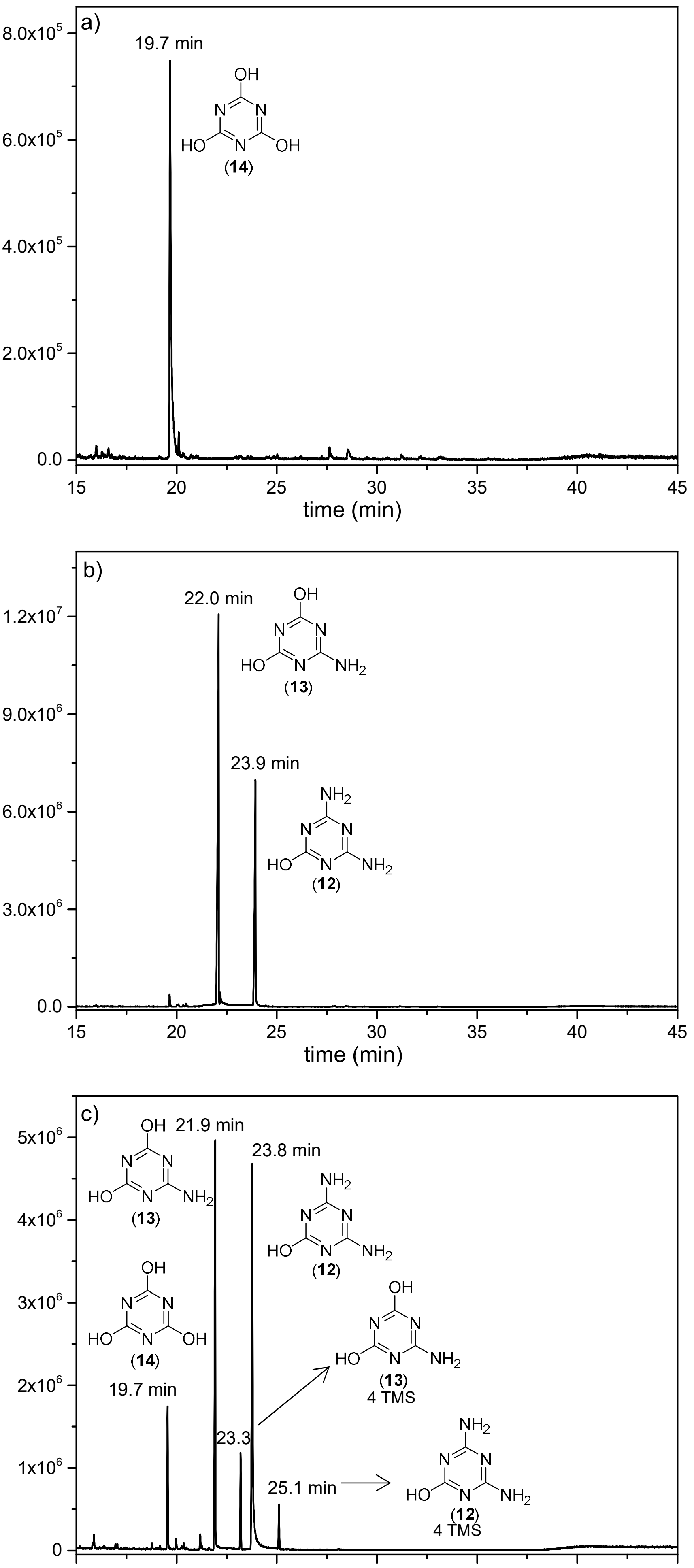

Figure S26. GC-MS chromatograms of a standar of ammeline (**12**) after: a) Acid hydrolysis; b) Basic hydrolysis; and c) Neutral hydrolysis; and lately derivatization with BSTFA to obtain the corresponding TMS-derivatives.

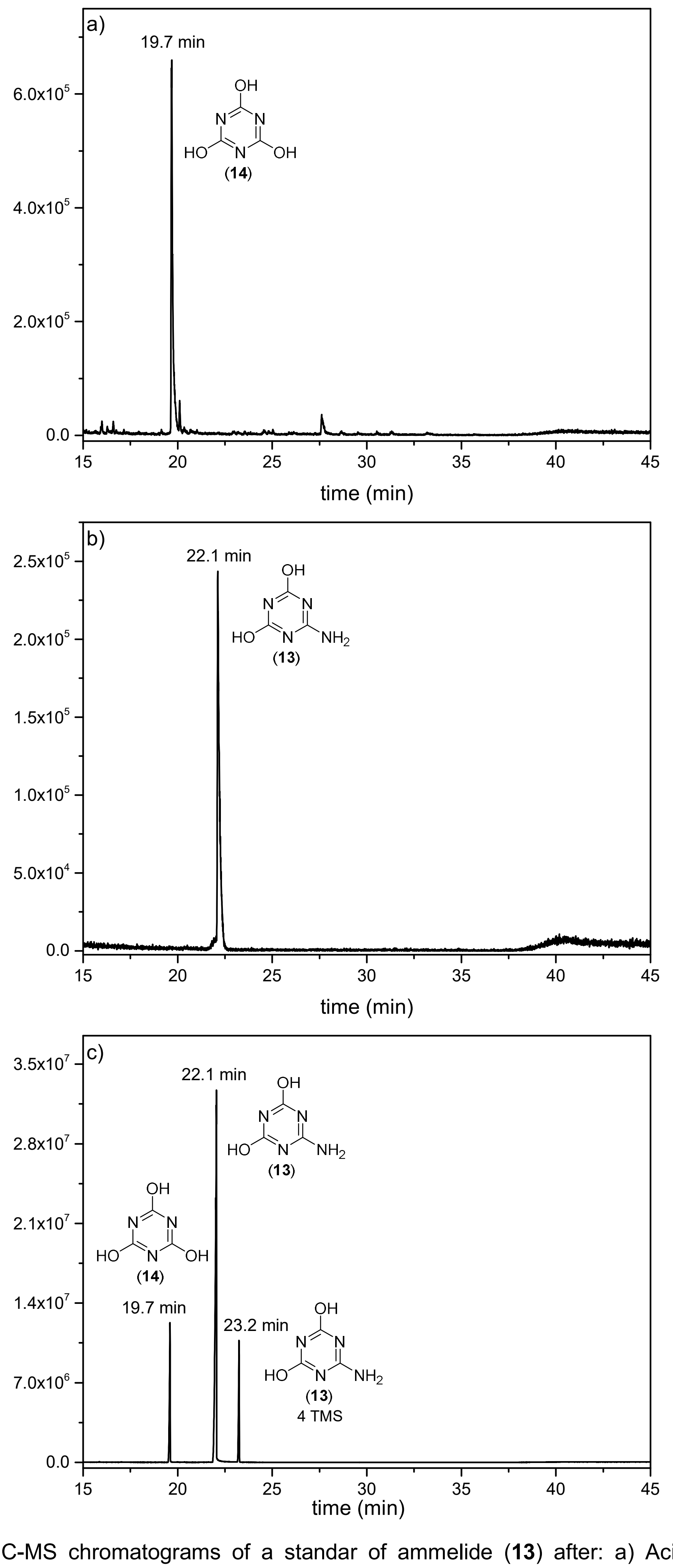

Figure S27. GC-MS chromatograms of a standar of ammelide (**13**) after: a) Acid hydrolysis; b) Basic hydrolysis; and c) Neutral hydrolysis; and lately derivatization with BSTFA to obtain the corresponding TMS-derivatives.

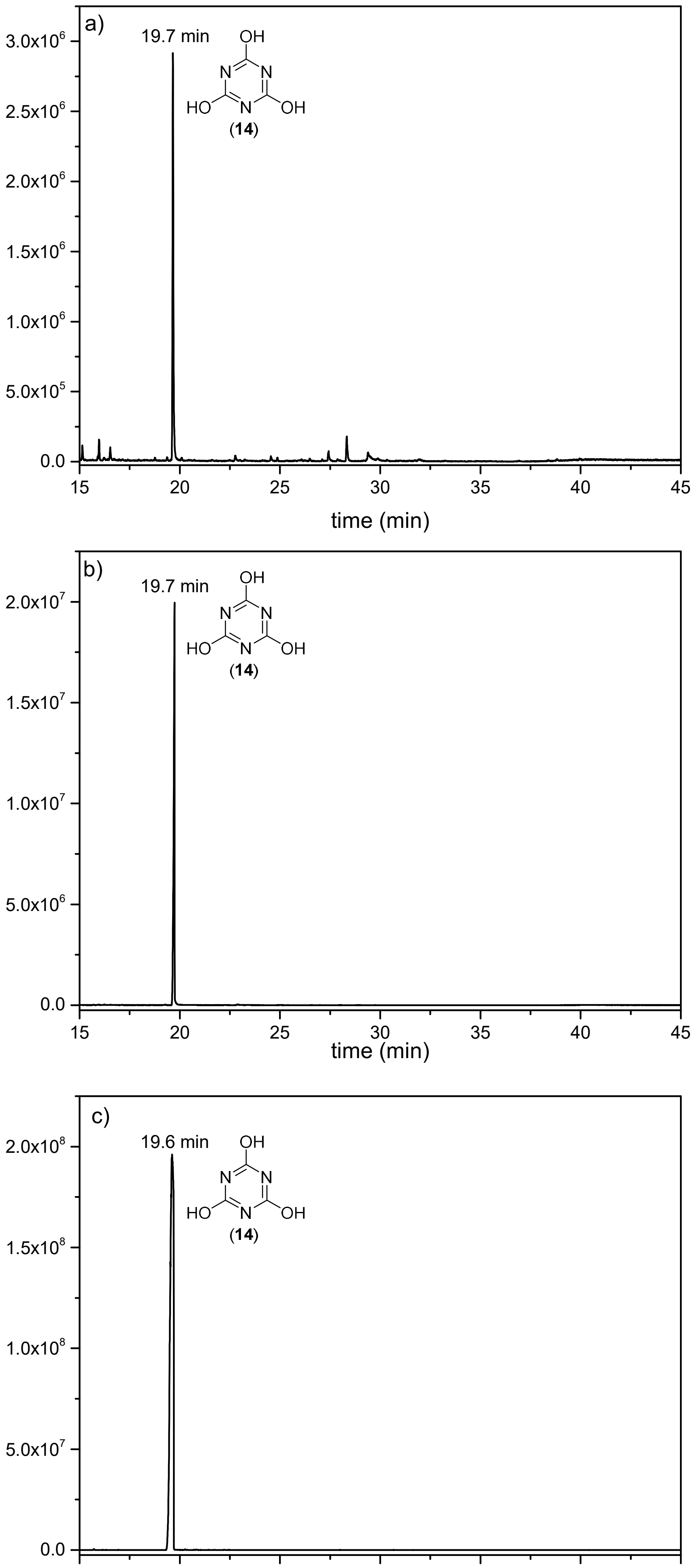

Figure S28. GC-MS chromatograms of a standar of cyanuric acid (**14**) after: a) Acid hydrolysis; b) Basic hydrolysis; and c) Neutral hydrolysis; and lately derivatization with BSTFA to obtain the corresponding TMS-derivatives.

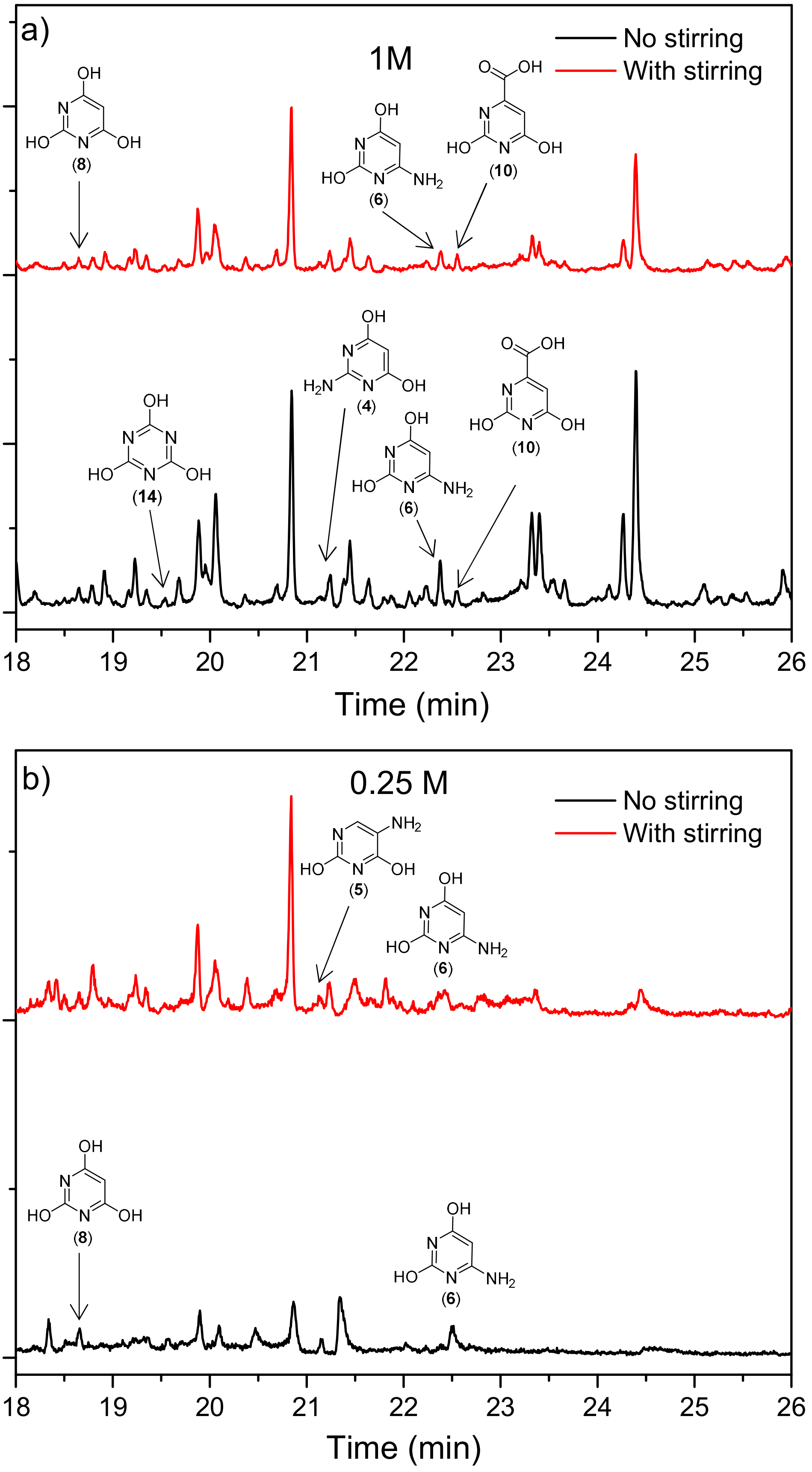

Figure S29. Representative GC-MS chromatograms from the freeze-dried crude reactions of the MW-driven polymerization of cyanide at 180 ºC using a reaction time of 20 min, in static (no stirring) or with stirring experiments at different cyanide concentrations, a) 1 M and b) 0.25 M, using the GC-MS ramp develop for the analysis of pyrimidines and triazines. In both chromatograms, using a concentration of 1 M, uracil (**2**) is detected at 15.90 min. ≈ 5 mg from the freeze-dried crudes reactions were derivatized in all the cases. The analytes were identified as their TMS-derivatives. The tautomeric form identified is represented.

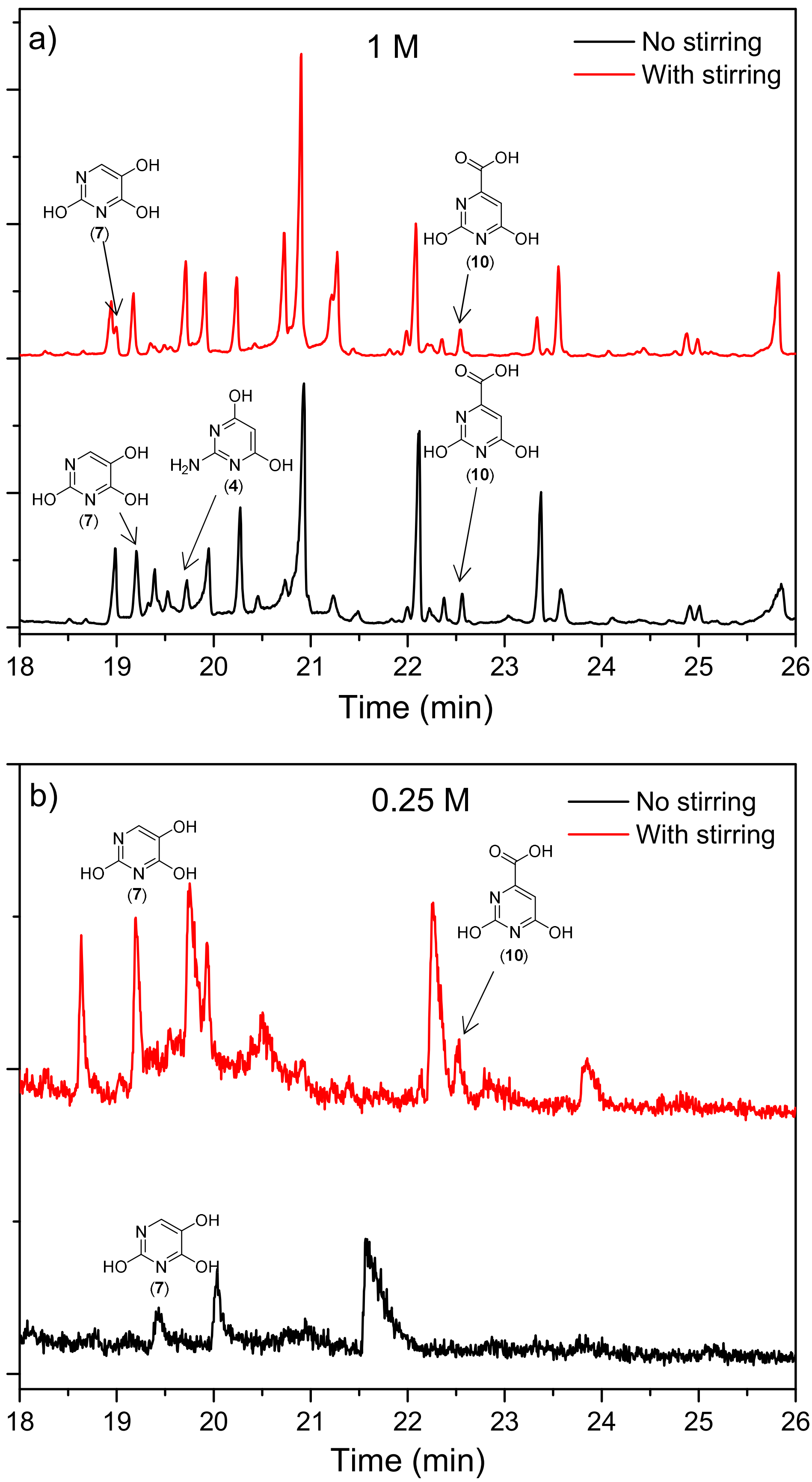

Figure S30. Representative GC-MS chromatograms from the freeze-dried crude reactions of the MW-driven polymerization of cyanide at 180 ºC using a reaction time of 20 min, after acid hydrolyis conditions (HCl 6N / 110 ºC / 24h) in static (no stirring) or with stirring experiments at different cyanide concentrations, a) 1M and b) 0.25 M, using the GC-MS ramp develop for the analysis of pyrimidines and triazines. In both chromatograms, using a concentration of 1 M, uracil (**2**) is detected at 15.90 min. ≈ 20 mg from the freeze-dried crudes reactions of sample were hydrolized in all the cases. The analytes were identified as their TMS-derivatives. The tautomeric form identified is represented.

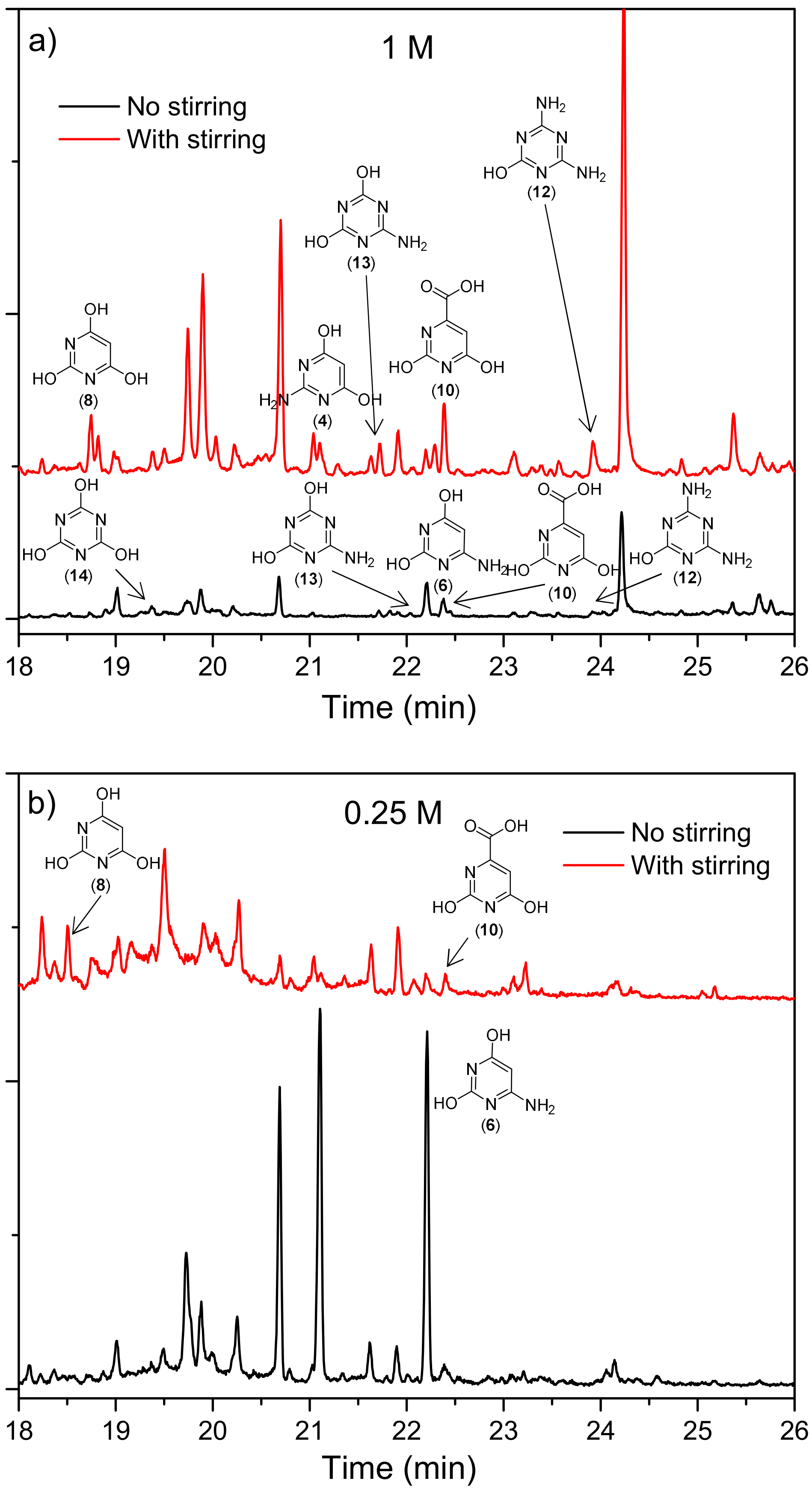

Figure S31. Representative GC-MS chromatograms from the freeze-dried crude reactions of the MW-driven polymerization of cyanide at 180 °C using a reaction time of 20 min, after basic hydrolyis conditions (NH_4OH 6N / 110 °C / 24h) in static (no stirring) or with stirring experiments at different cyanide concentrations, a) 1M and b) 0.25 M, using the GC-MS ramp develop for the analysis of pyrimidines and triazines. ≈ 20 mg of sample were hydrolized in all the cases. The analytes were identified as their TMS-derivatives. The tautomeric form identified is represented.

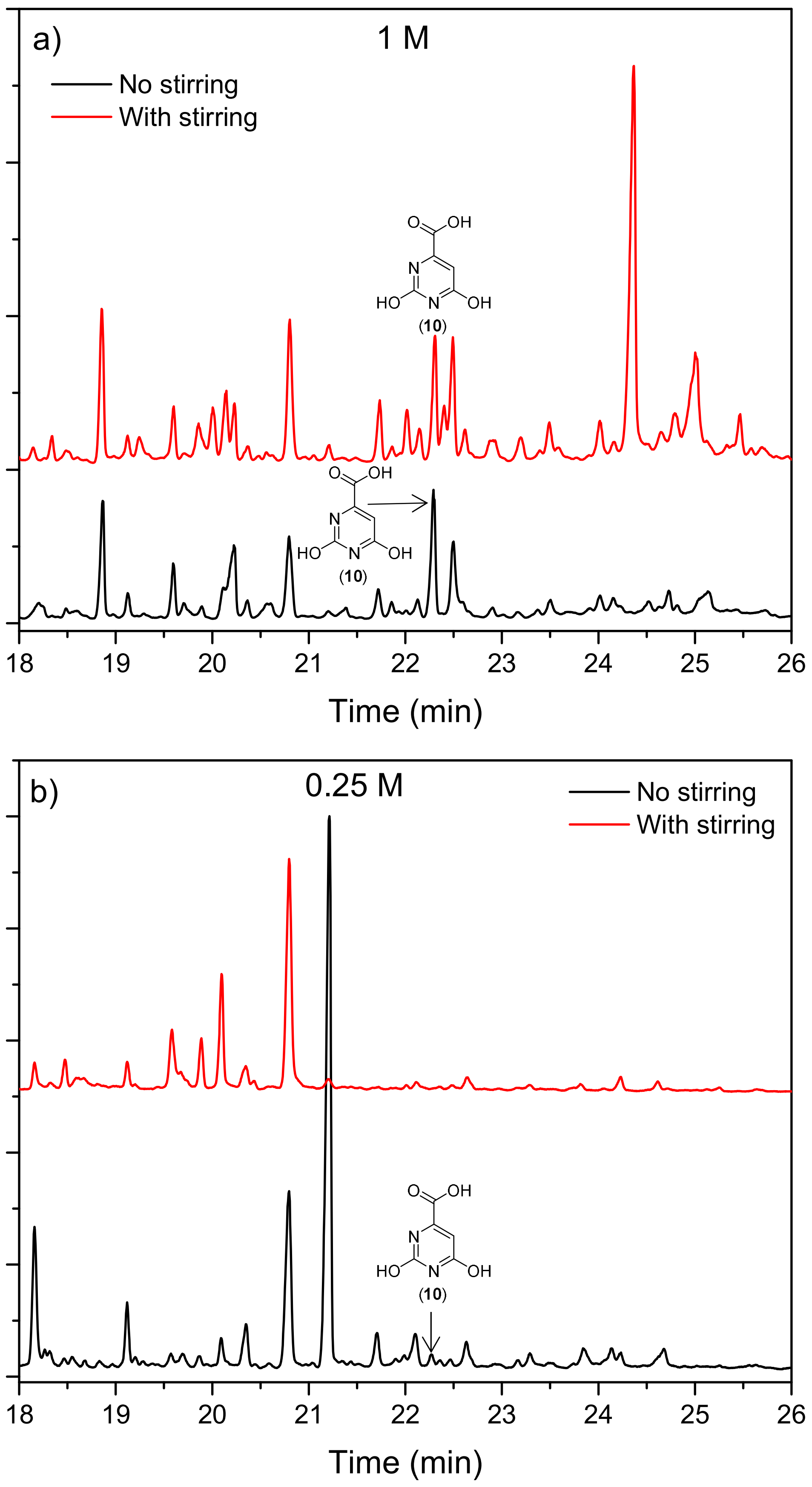

Figure S32. Representative GC-MS chromatograms from the freeze-dried crude reactions of the MW-driven polymerization of cyanide at 180 ºC using a reaction time of 20 min, after neutral hydrolyis conditions (0.01 M PO_4^{-3}, pH 8 / 140 ºC / 72 h) in static (no stirring) or with stirring experiments at different cyanide concentrations, a) 1M and b) 0.25 M, using the GC-MS ramp develop for the analysis of pyrimidines and triazines. ≈ 30 mg of sample were hydrolized in all the cases. The analytes were identified as their TMS-derivatives. The tautomeric form identified is represented.

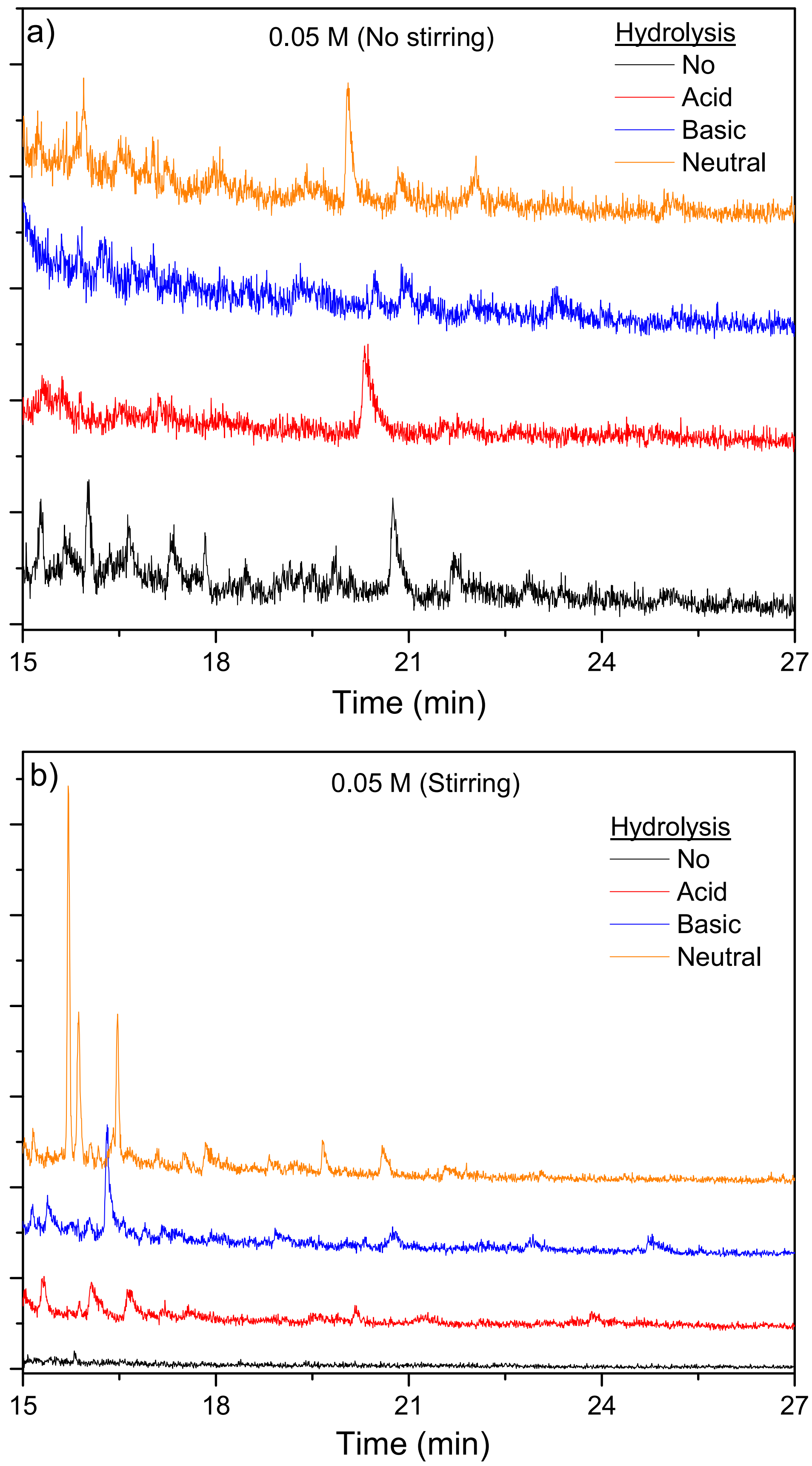

Figure S33. Representative GC-MS chromatograms from the freeze-dried crude reactions of the MW-driven polymerization of cyanide (initial concentration 0.05 M) at 180 ºC using a reaction time of 20 min, after severla hydrolysis conditions, in a) static experiments and b) experiments with stirring.

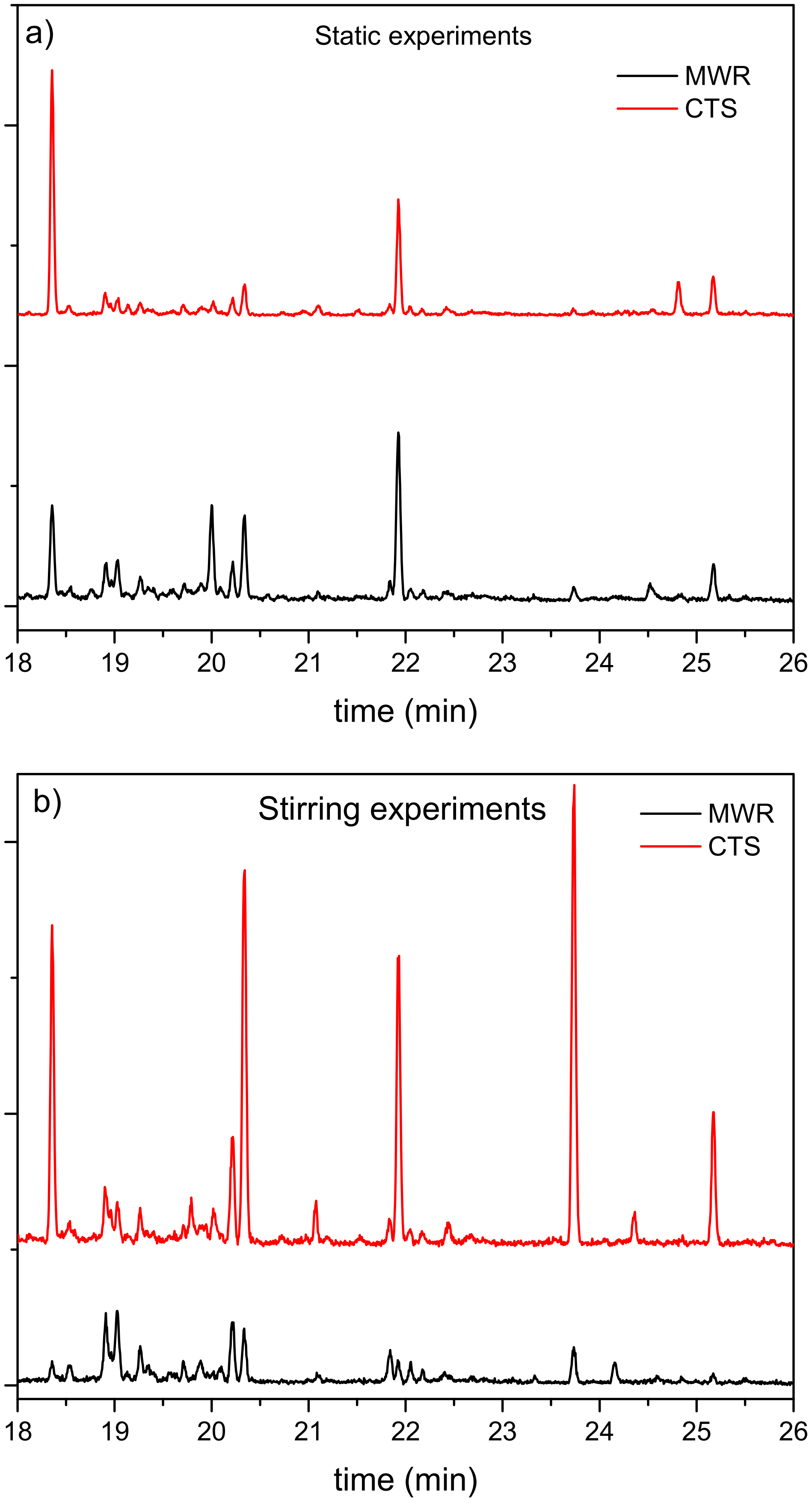

Figure S34. Representative GC-MS chromatograms from the gel fractions, collected by filtration and dried using reduce pressure, from the cyanide polymerization (1M) at 180 ºC (MWR = microwave radiation) or at 80 ºC (CTS = conventional thermal system) in: a) static experiments; b) stirring experiments. ≈ 5 mg were derivatized in all the cases. No N-heterocycles were identified in any of these samples.

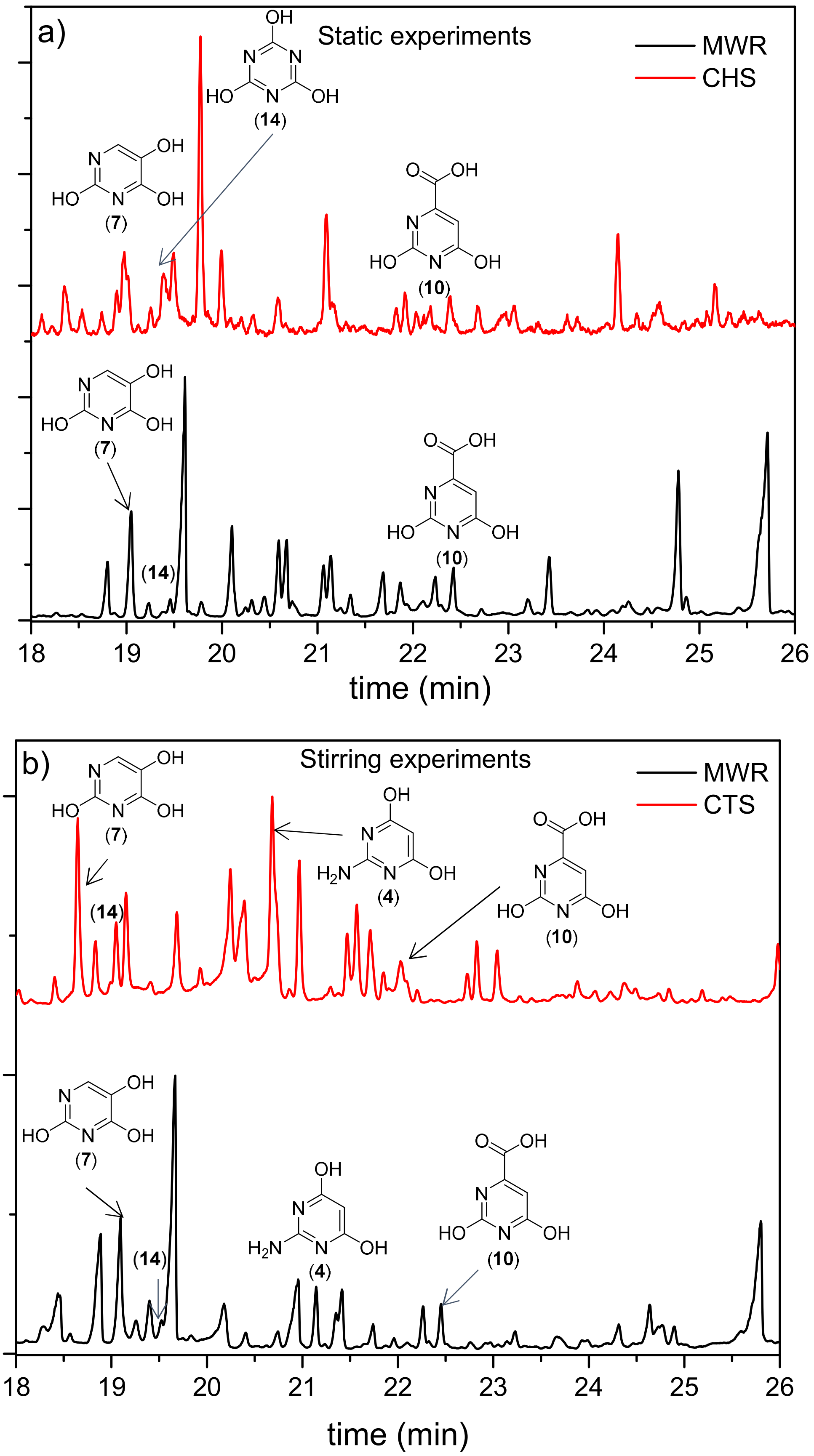

Figure S35. Representative GC-MS chromatograms from the gel fractions, collected by filtration and dried using reduce pressure, from the cyanide polymerization (1 M) at 180 ºC (MWR = microwave radiation) or at 80 ºC (CTS = conventional thermal system) in: a) static experiments; b) stirring experiments, after acid hydrolysis conditions and derivatization with BSTFA. ≈ 10 mg of sample were hydrolized in all the cases. The analytes were identified as their TMS-derivatives. The tautomeric form identified is represented.

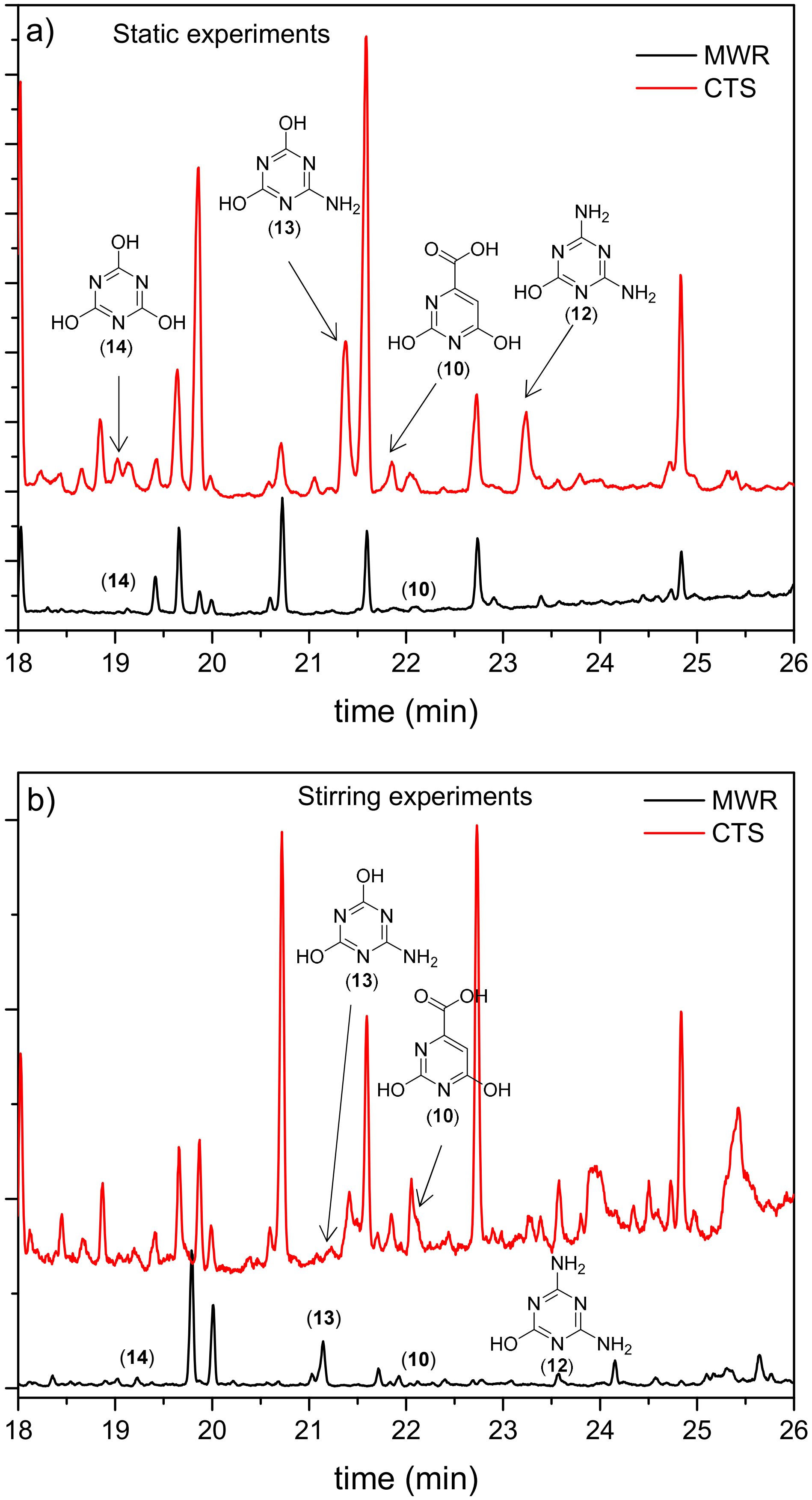

Figure S36. Representative GC-MS chromatograms from the gel fractions, collected by filtration and dried using reduce pressure, from the cyanide polymerization (1M) at 180 ºC (MWR = microwave radiation) or at 80 ºC (CTS = conventional thermal system) in: a) static experiments; b) stirring experiments, after basic hydrolysis conditions and derivatization with BSTFA. ≈ 10 mg of sample were hydrolized in all the cases. The analytes were identified as their TMS-derivatives. The tautomeric form identified is represented.

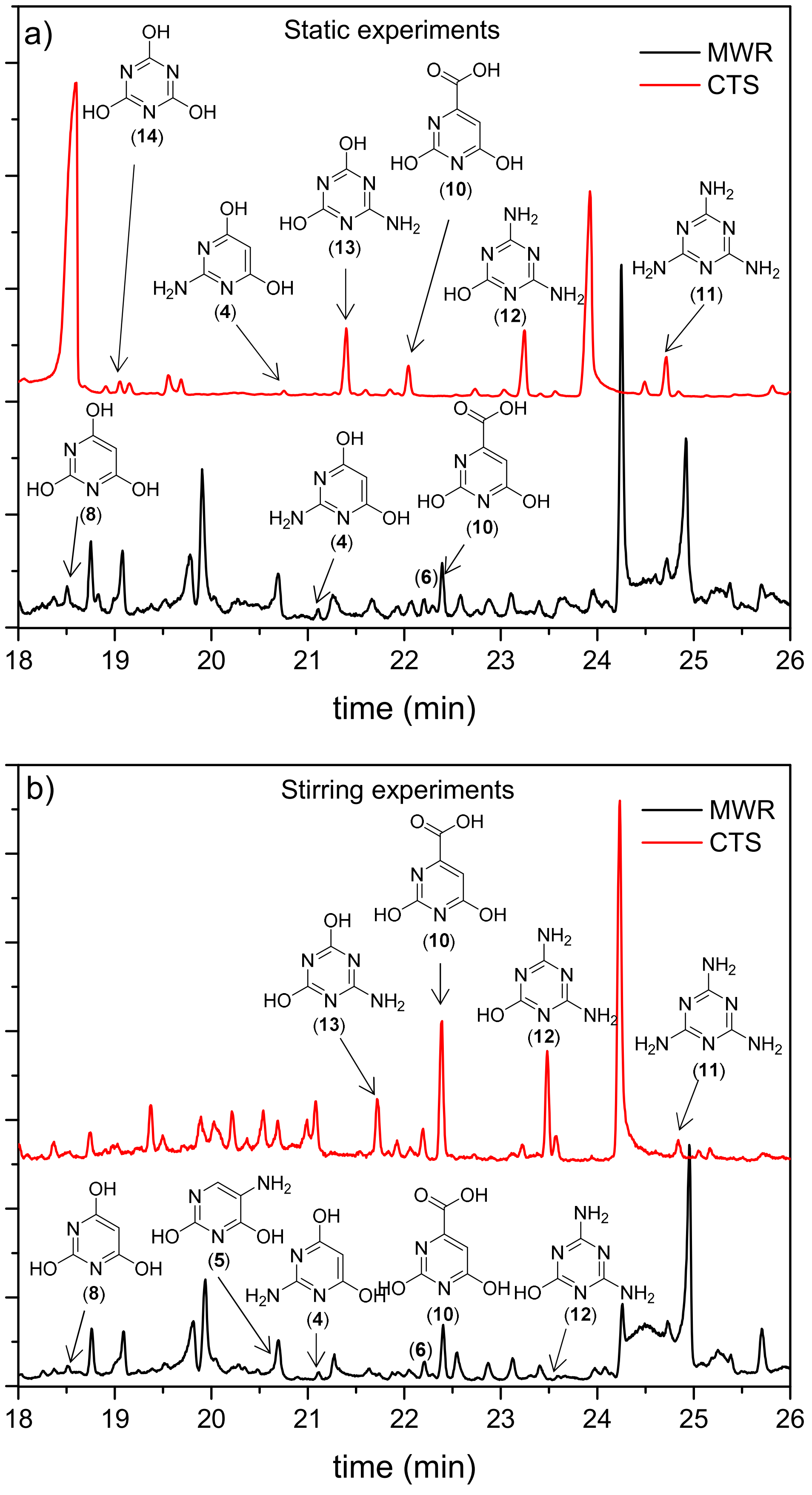

Figure S37. Representative GC-MS chromatograms from the sol fractions, collected after filtration and freeze-dried, from the cyanide polymerization (1M) at 180 ºC (MWR = microwave radiation) or at 80 ºC (CTS = conventional thermal system) in: a) static experiments; b) stirring experiments. ≈ 20 mg were derivatized with BSTFA in all the cases. The analytes were identified as their TMS-derivatives. The tautomeric form identified is represented.

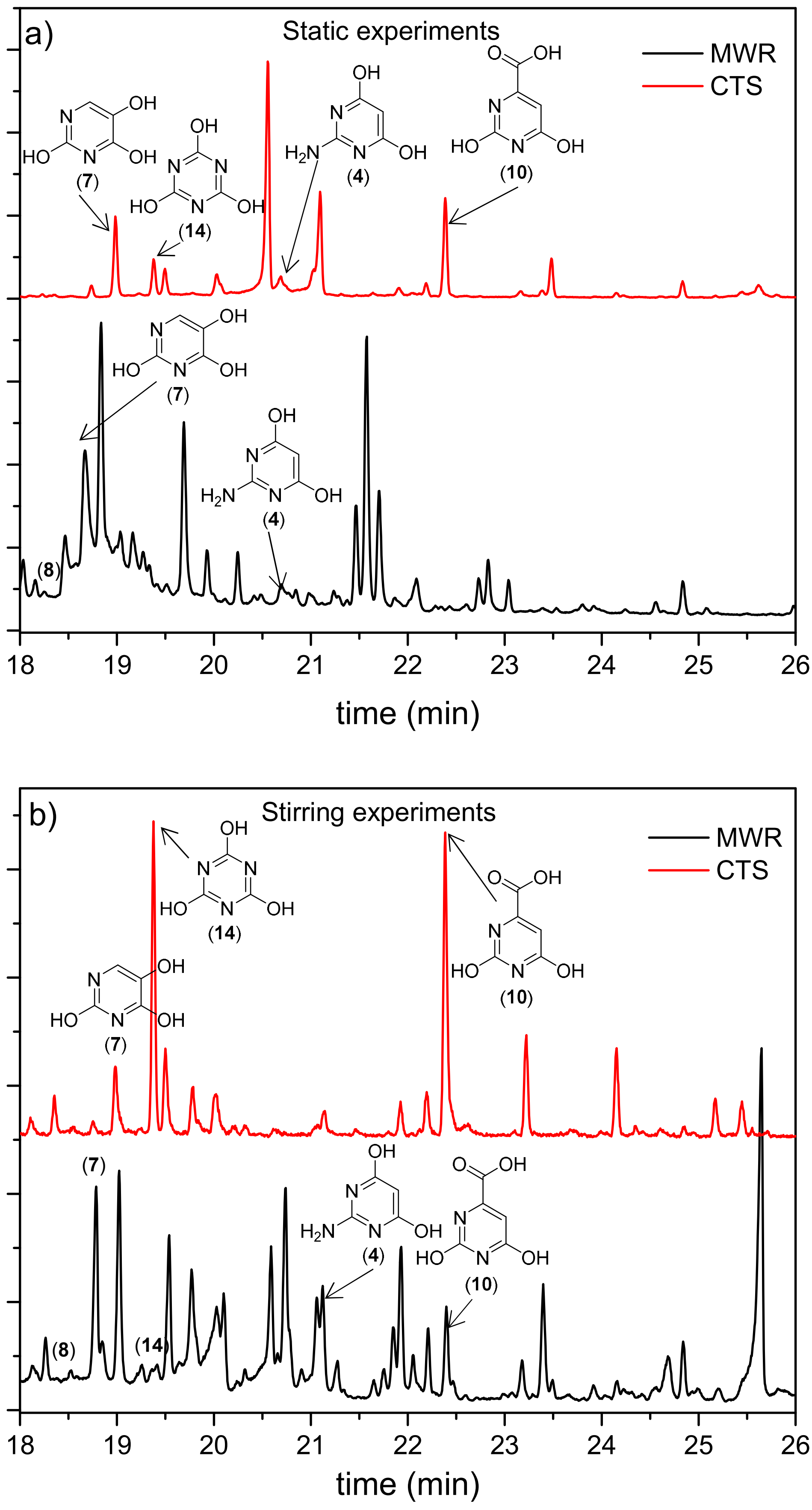

Figure S38. Representative GC-MS chromatograms from the sol fractions, collected after filtration and freeze-dried, from the cyanide polymerization (1M) at 180 ºC (MWR = microwave radiation) or at 80 ºC (CTS = conventional thermal system) in: a) static experiments; b) stirring experiments, after acid hydrolysis conditions and derivatization with BSTFA. ≈ 15 mg of sample were hydrolized in all the cases. The analytes were identified as their TMS-derivatives. The tautomeric form identified is represented.

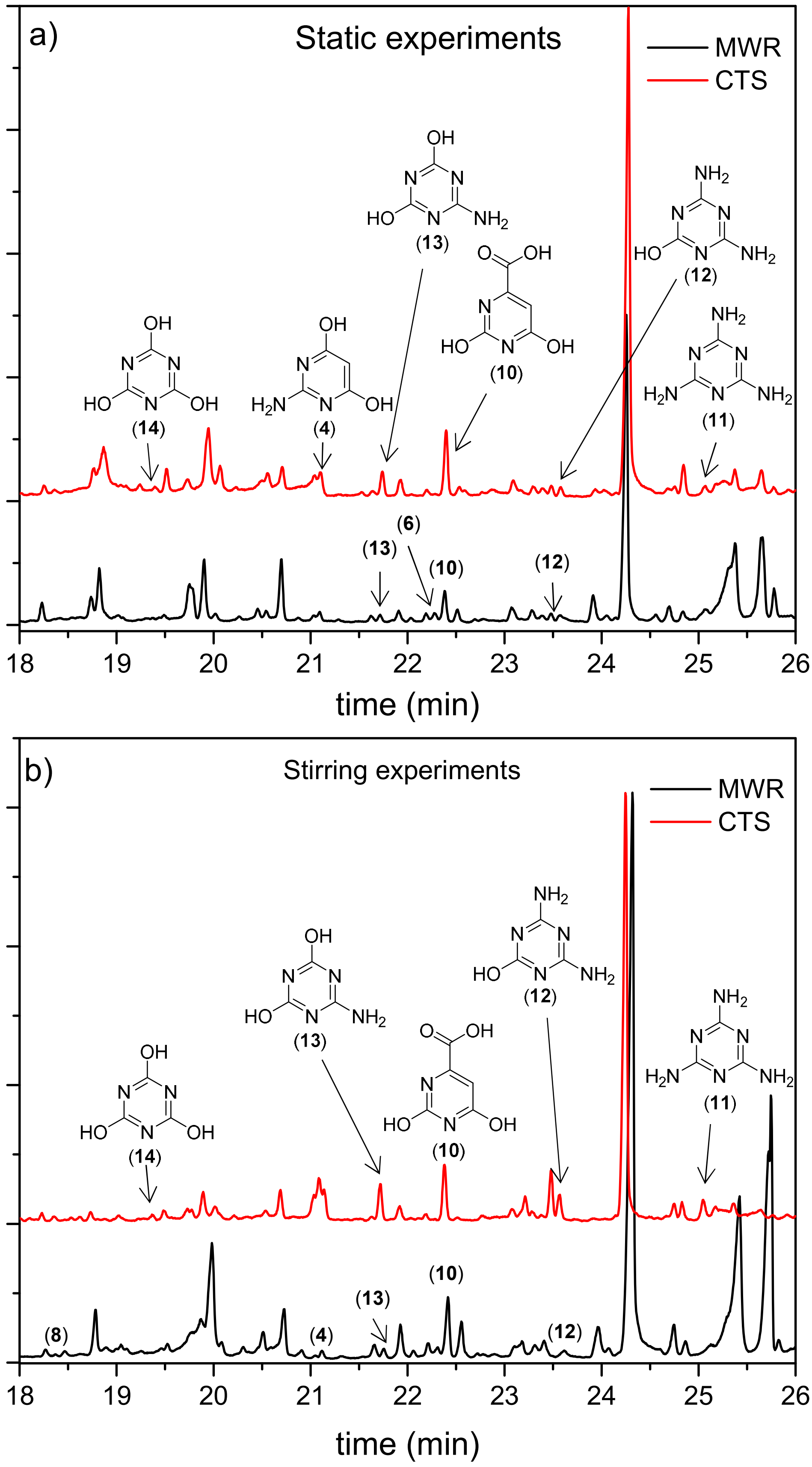

Figure S39. Representative GC-MS chromatograms from the sol fractions, collected after filtration and freeze-dried, from the cyanide polymerization (1M) at 180 ºC (MWR = microwave radiation) or at 80 ºC (CTS = conventional thermal system) in: a) static experiments; b) stirring experiments, after basic hydrolysis conditions and derivatization with BSTFA. ≈ 12 mg of sample were hydrolized in all the cases. The analytes were identified as their TMS-derivatives. The tautomeric form identified is represented.

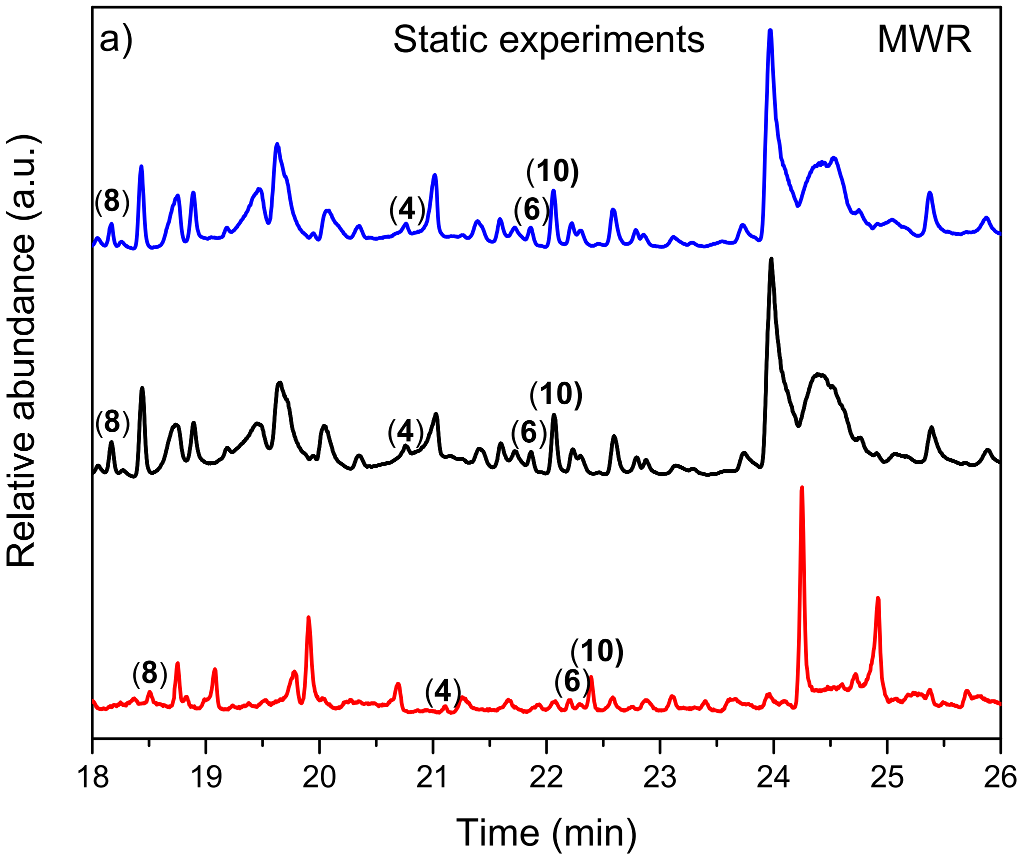

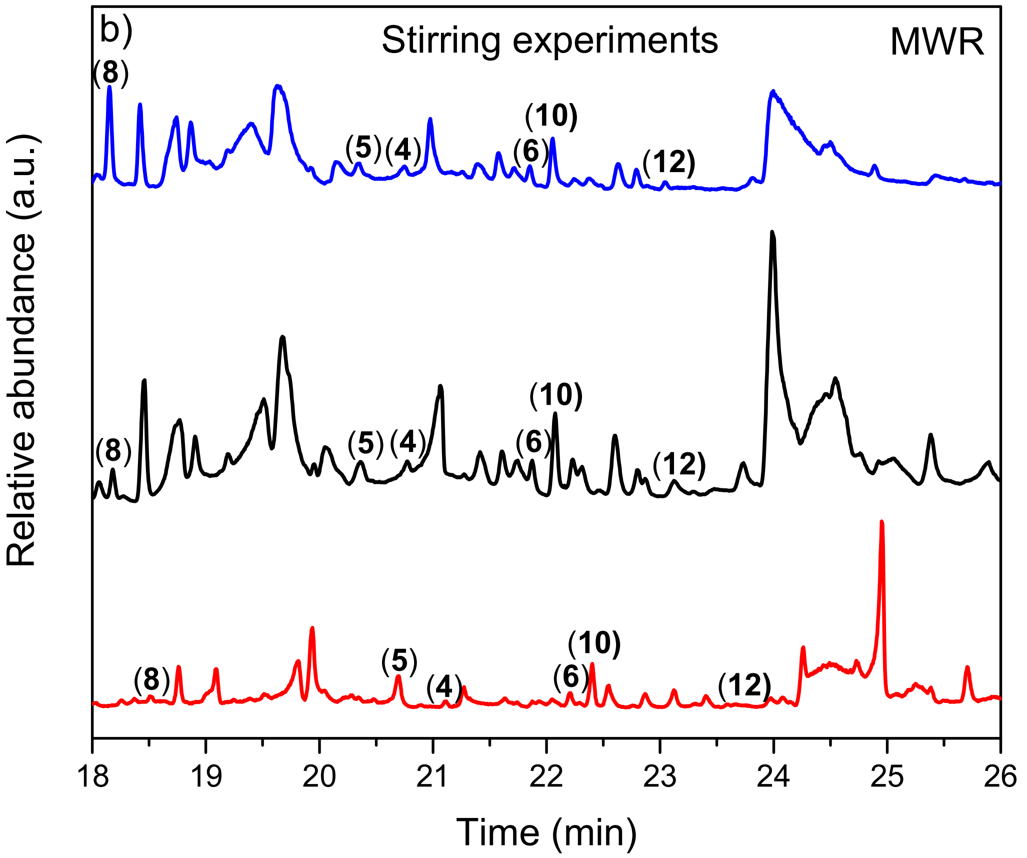

Figure S40. Representative GC-MS chromatograms from three independent cyanide polymerizations (1M) carried out at 180 ºC (MWR = microwave radiation), corrresponding with the experimental details showed in the Figure S37. a) polymerizations carried out without stirring (static experiments); b) polymerizations carried out with stirring (stirring experiments). The differences in the intensity of the chromatographic peaks are related with the use of different amounts of sample. The shift in the retention times are due to the change of the chromatographic column along the development of these analytical study.

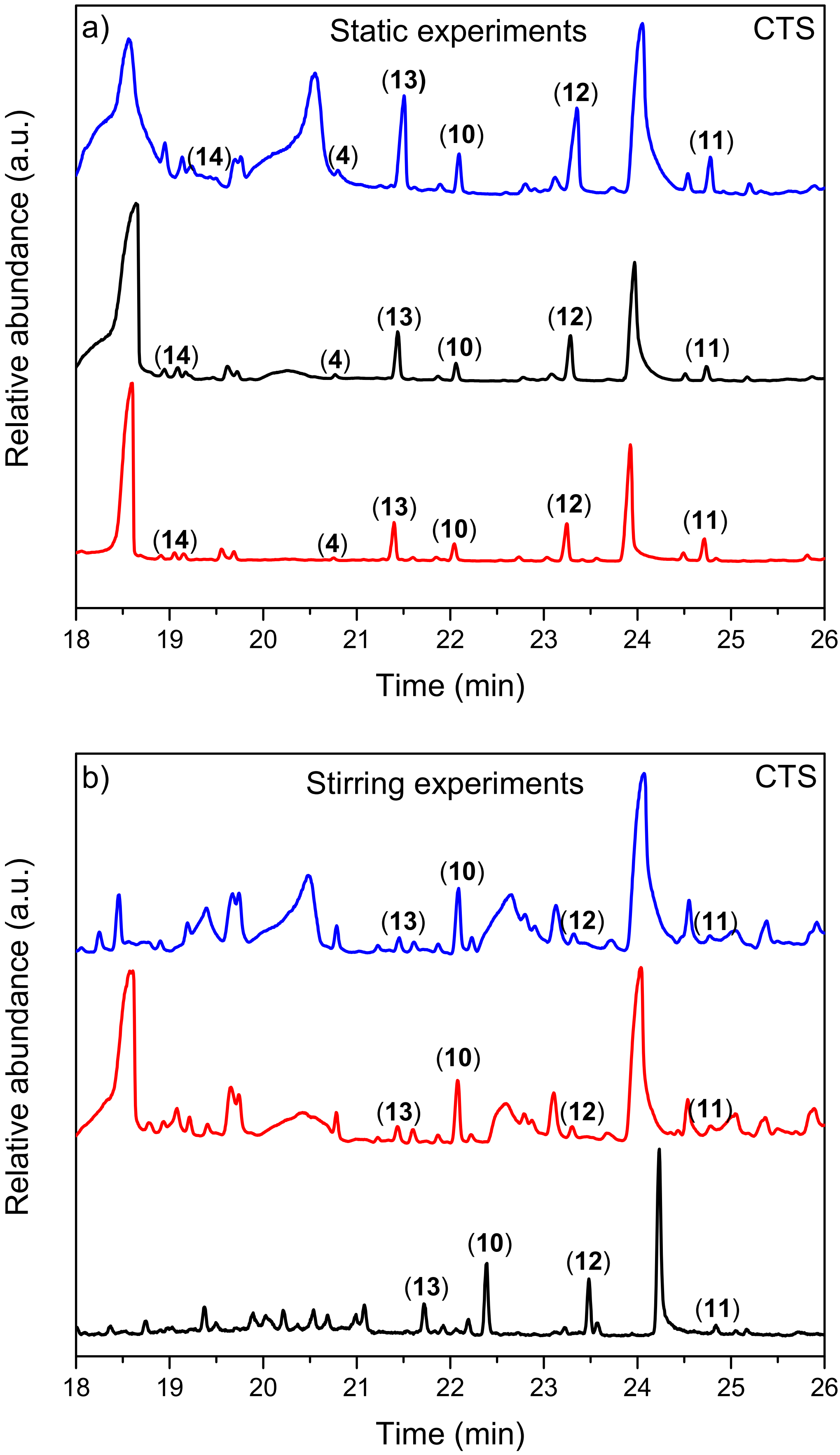

Figure S41. Representative GC-MS chromatograms from three independent cyanide polymerizations (1M) carried out at 80 ºC (CTS = convenctional termal system), corrresponding with the experimental details showed in the Figure S37. a) polymerizations carried out without stirring (static experiments); b) polymerizations carried out with stirring (stirring experiments). The differences in the intensity of the chromatographic peaks are related with the use of different amounts of sample. The shift in the retention times are due to the change of the chromatographic column along the development of these analytical study.
